# Cannabinoids in Integumentary Wound Care: A Systematic Review of Emerging Preclinical and Clinical Evidence

**DOI:** 10.3390/pharmaceutics16081081

**Published:** 2024-08-17

**Authors:** Dhakshila Niyangoda, Mohammed Muayad, Wubshet Tesfaye, Mary Bushell, Danish Ahmad, Indira Samarawickrema, Justin Sinclair, Shida Kebriti, Vincent Maida, Jackson Thomas

**Affiliations:** 1 Faculty of Health, University of Canberra, Canberra, ACT 2617, Australia; dhakshila.niyangoda@canberra.edu.au (D.N.); u3163495@uni.canberra.edu.au (M.M.); mary.bushell@canberra.edu.au (M.B.); 2 Department of Pharmacy, Faculty of Allied Health Sciences, University of Peradeniya, Peradeniya 20400, Sri Lanka; 3 School of Pharmacy, Faculty of Health and Behavioural Sciences, University of Queensland, Queensland, QLD 4072, Australia; w.tesfaye@uq.edu.au; 4 School of Medicine and Psychology, Australian National University, Canberra, ACT 2601, Australia; danish.ahmad@anu.edu.au; 5 Strategy Coaching and Research Consulting Pty Ltd., O’Malley, ACT 2606, Australia; info@indirasam.com; 6 Australian Natural Therapeutics Group, Byron Bay, NSW 2481, Australia; justin.sinclair@australiannatural.com; 7 NICM Health Research Institute, Western Sydney University, Westmead, NSW 2145, Australia; 8 Eczanes Pharmaceuticals, Rydalmere, NSW 2116, Australia; shida@eczanes.com.au; 9 Temerity Faculty of Medicine, University of Toronto, Toronto, ON M5S 1A8, Canada; vincent.maida@utoronto.ca; 10 Hospice Vaughan, Woodbridge, ON L4H 3G7, Canada

**Keywords:** wound healing, antibacterial, medicinal cannabis, cannabinoids, antimicrobial resistance

## Abstract

This systematic review critically evaluates preclinical and clinical data on the antibacterial and wound healing properties of cannabinoids in integument wounds. Comprehensive searches were conducted across multiple databases, including CINAHL, Cochrane library, Medline, Embase, PubMed, Web of Science, and LILACS, encompassing records up to May 22, 2024. Eighteen studies met the inclusion criteria. Eleven were animal studies, predominantly utilizing murine models (*n* = 10) and one equine model, involving 437 animals. The seven human studies ranged from case reports to randomized controlled trials, encompassing 92 participants aged six months to ninety years, with sample sizes varying from 1 to 69 patients. The studies examined the effects of various cannabinoid formulations, including combinations with other plant extracts, crude extracts, and purified and synthetic cannabis-based medications administered topically, intraperitoneally, orally, or sublingually. Four animal and three human studies reported complete wound closure. Hemp fruit oil extract, cannabidiol (CBD), and GP1a resulted in complete wound closure in twenty-three (range: 5–84) days with a healing rate of 66–86% within ten days in animal studies. One human study documented a wound healing rate of 3.3 cm^2^ over 30 days, while three studies on chronic, non-healing wounds reported an average healing time of 54 (21–150) days for 17 patients by oral oils with tetrahydrocannabinol (THC) and CBD and topical gels with THC, CBD, and terpenes. CBD and tetrahydrocannabidiol demonstrated significant potential in reducing bacterial loads in murine models. However, further high-quality research is imperative to fully elucidate the therapeutic potential of cannabinoids in the treatment of bacterial skin infections and wounds. Additionally, it is crucial to delineate the impact of medicinal cannabis on the various phases of wound healing. This study was registered in PROSPERO (CRD42021255413).

## 1. Introduction

Wound healing is a complex biological process involving four sequential and overlapping phases, haemostasis, inflammation, proliferation, and remodelling, which are mediated by various cellular and molecular events to restore tissue integrity [1,2,3]. The global prevalence of chronic wounds from 2000 to 2018 was approximately 2.21 per 1000 population [4]. Managing wounds imposes significant financial burdens on health systems worldwide, consuming 2–4% of national health expenditures and costing about USD 30,000 per episode in 2015 [5,6,7,8,9]. However, the clinical and financial burden of chronic wounds—including high disability-adjusted life years, reduced quality of life, and in severe cases, amputation and death—remains vastly underestimated [10,11,12].

Integument, or skin and mucous membranes, act as a physiological barrier between the inside and outside of organisms [13]. Acute dermal wounds also known as partial-thickness wounds, while they generally heal autonomously, may become complicated by infections and excessive scar formation, leading to symptoms such as wound pain and impaired healing. Wound healing in dermal and mucus membranes are mechanistically similar, although the scarring and time-to-heal outcomes are improved in mucosal healing [14,15,16,17,18]. Chronic wounds are particularly challenging to treat as they remain susceptible to pathogenic microbes, including antimicrobial-resistant strains, resulting in localized and potentially fatal systemic infections [2,19,20,21,22,23,24,25,26,27]. The rise in antimicrobial resistance exacerbates these challenges, leading to treatment failures, prolonged morbidity, and increased healthcare expenditures [28,29,30,31]. Despite this pressing need, the development of new antimicrobials remains slow [32,33,34,35]. Current treatments for chronic wounds include conventional antibiotic therapy, wound dressings, hyperbaric oxygen therapy, and negative-pressure wound therapy, alongside advanced methods such as skin grafting, growth factor supplementation, collagen sponges, and tissue-engineered products [20,23,36,37,38]. These methods are often labour-intensive, technologically sophisticated, and less accessible in resource-poor settings. Topical agents with antibacterial properties offer advantages in wound management, facilitating rapid healing without systemic effects and ensuring better compliance [39].

Medicinal cannabis (MC) also known as medicinal marijuana, cannabis-based medicine, or medical cannabis, is a diverse group of chemical compounds and *Cannabis sativa* plant extracts that can bind to receptors in the endocannabinoid system—mainly with cannabinoid receptor type 1 (CB1R) and cannabinoid receptor type 2 (CB2R)—and exert cannabinomimetic effects [40,41]. Cannabinoids are classified as endocannabinoids, phytocannabinoids, and synthetic cannabinoids [40,41,42]. Both *C. sativa* plant extracts and cannabinoids are utilized for therapeutic purposes and exhibit compelling antibacterial effects against a broad spectrum of pathogens, particularly Gram-positive bacteria, including methicillin-resistant *Staphylococcus aureus* (MRSA) [43,44,45,46,47,48,49,50,51,52,53,54,55,56,57,58,59]. MC has a unique advantage in wound healing through its action on the endocannabinoid system (ECS), the body’s most extensive neurotransmitter system [60]. The cutaneous ECS, or c(ut)annabinoid system, is crucial for maintaining skin homeostasis, including physiological wound healing [61,62,63]. Evidence suggests that cannabinoids modulate key molecular pathways of wound healing through CB1R agonism, CB2R agonism/antagonism, or by acting on receptors such as TRPA1, PPARγ, and GPR55 [63,64,65,66,67]. Cannabinoids promote chronic wound healing by regulating keratinocyte proliferation and differentiation at the wound edge, modulating matrix metalloproteinases (MMPs), reducing pro-inflammatory cytokines, shifting macrophage responses from a pro-inflammatory M1 to an anti-inflammatory M2 profile, and reducing nitric oxide signalling at the wound bed [65]. Activation of CB2R decreases the expression of collagen I and III, MMP-1, and MMP-3, while increasing the expression of the tissue inhibitor of metalloprotease-1 (TIMP-1), MMP-2, and MMP-9 [65,68,69,70,71]. CB1R and CB2R agonists also inhibit the release of sulfated glycosaminoglycans [72,73]. In acute wound healing, CB2R modulation accelerates wound closure and regulates fibrogenesis, potentially minimizing scarring [65,67,68,74,75]. Cannabinoids possess various therapeutic properties, including appetite stimulation, antioxidant, analgesic, angiogenic, anti-inflammatory, and skin moisturizing effects, all beneficial for wound healing [76,77,78,79,80]. Additionally, cannabinoids regulate the reactive oxygen species production during wound healing, with tetrahydrocannabinol (THC) and cannabidiol (CBD) exhibiting antioxidant activity comparable to that of vitamins C and E [3,76].

MC has been studied for its clinical benefits in treating pain, mental illnesses, neurological conditions, palliative care, and dermatological conditions such as acne vulgaris, dermatitis, eczematous dermatoses, hidradenitis suppurativa, Kaposi sarcoma, melanoma, pruritus, psoriasis, scleroderma, and wound healing [81,82,83,84,85,86,87,88,89,90,91,92,93,94]. Recent studies have explored its potential in pediatric dermatology [95]. While generally safe and well-tolerated in adults, oral and oro-muscular dosage forms of MC had shown mild to moderate adverse effects [96,97,98,99,100,101]. Topical CBD is non-sensitizing and nonirritating in healthy adults on short-term use [102], and both topical and transdermal MC, including CBD, typically cause no or minor adverse effects in humans in short-term use [102,103,104,105,106]. There is an increasing trend of patients requesting MC prescriptions worldwide [107,108].

Despite numerous narrative reviews highlighting the potential of cannabinoids in wound healing [61,62,64,65,109,110,111,112,113,114,115], specific details regarding types of integument wounds remain underexplored. Some reviews have summarised molecular mechanisms of wound healing, ex vivo, animal models, and human wound healing by cannabinoids with varying levels of detail [61,64,65,109,110,112]. Others have elaborated on the effects of CBD in wound healing [62,113,114]. Reviews summarising the antimicrobial potential of cannabinoids have focused on structure–activity relationships, in vitro studies, and in vivo studies, regardless of the affected organ or system [47,56,113,116,117]. Despite these insights, a systematic review explicitly focusing on the role of MC in promoting wound healing has not been conducted. This systematic review aims to evaluate methodically the current evidence on the wound healing and antibacterial properties of cannabinoids in treating integumentary wounds and infections, whether these compounds are used alone or in combination with other agents.

## 2. Materials and Methods

### 2.1. Search Strategy

This systematic review was conducted in accordance with the Preferred Reporting Items for Systematic Review and Meta-Analysis guidelines 2020 [118] (Tables S1 and S2) and is registered with PROSPERO (CRD42021255413) [119]. The search was conducted from inception to 22 May 2024. The initial search was performed using major electronic databases, including the Cumulative Index to Nursing and Allied Health Literature (CINAHL), Cochrane library, Medline, Embase via Scopus, PubMed, Web of Science, and the Latin America and Caribbean Health Sciences Literature (LILACS). Corresponding authors were contacted to retrieve articles that were not readily available. Searches were conducted without any restrictions on the date of publication or language. Variations in the following search strategy were used depending on the information source: (cannabis OR cannabinoid AND (wound OR (antibacterial AND skin)). The exact search strategy for each database is provided in Supplementary material S1. This includes the main database search, the grey literature search, and additional searches such as in selected journals, the bibliographies of related systematic reviews, and individual studies.

### 2.2. Eligibility Criteria

This review encompasses both animal and human studies investigating the potential of MC as an antibacterial and/or wound healing agent. The studies evaluated the use of extracts derived from *C. sativa* and/or cannabinoids as a treatment strategy for any type of integument wounds or bacterial infections, comparing the interventions with untreated controls, conventional treatments, placebos, or no treatment. Eligible study types included preclinical studies, randomized and non-randomized controlled trials, observational studies, case series, and case studies. Correspondences, editorials, letters to editors, the literature reviews, scoping reviews, systematic reviews, and meta-analyses were excluded. Chronic wounds were defined as those that had not healed after three weeks or longer, or those described as complex or hard to heal, while all other wounds were categorized as acute for this review [4]. Synthetic cannabinoids are compounds that act as agonists of either CB1R or CB2R, regardless of their structure [120,121,122]. For this review, treatments involving MC that result in outcomes related to integumentary wound management and infections were considered eligible. 

The main outcomes were wound healing rate, time to complete wound healing, bacterial clearance rate, and bacterial clearance time. Wound healing rate was defined as the percentage of the initial area healed per day, with the wound area serving as the respective measurement [123]. Time to complete wound healing was defined as the number of days required to achieve complete wound closure, with initial and final wound areas serving as the respective measurements. Complete wound healing, also known as epithelialisation time, referred to the complete epithelialisation of the wound without the need for cleaning or dressing [124,125]. Bacterial clearance time referred to the number of days between the initial treatment and the day on which the ulcer swab culture became negative [124]. Other outcomes related to wounds were also extracted. Adverse effects were documented and reported.

After completing the search, the retrieved articles were imported into EndNote 20.1 (Clarivate 2021, Philadelphia, US) and then into the Covidence systematic review software (2024 Covidence, Veritas Health Innovation, Melbourne, Australia) for screening. Ex vivo studies, although excluded from the systematic review, were screened, and summarised (Table S3) to provide a comprehensive understanding of the wound healing and antibacterial properties of *C. sativa* extracts and cannabinoids. Preclinical and clinical studies that reported wound healing properties but did not meet the inclusion criteria were summarised in Tables S4–S7. The major outcomes reported by the studies were categorized as preclinical and clinical studies and synthesized narratively, presented in a tabulated form.

### 2.3. Quality Assessment

The study selection, data extraction, and quality assessment were carried out independently by two members of the review team (DN, MM, WT, or MB), with any discrepancies resolved through discussion or consultation with a third researcher (JT). In vivo studies were evaluated for risk of bias (RoB) using the Toxicological Data Reliability Assessment Tool (ToxRTool 2009, European Commission’s Joint Research Centre, Ispra, Italy) [126]. Studies were included in our systematic review if they were classified as reliability category 1 (reliable without restrictions) or 2 (reliable with restrictions) but were excluded if assessed as reliability category 3 (not reliable).

The methodological quality of human studies was evaluated with the Joanna Briggs Institute (JBI) checklists for case reports (four or fewer individual cases), case series (grouped data of five or more patients), and randomized controlled trials [127,128,129,130]. All eligible human studies were included regardless of quality. Further details on these tools are provided in Supplementary material S2. Rejected articles are listed in Supplementary material S3, while the outcome measures of selected studies are provided in Table S8. Any changes made to the PROSPERO registration after the initial submission are outlined in [App app1-pharmaceutics-16-01081]Supplementary material S4.

## 3. Results

### 3.1. Search Results

**Figure 1:**
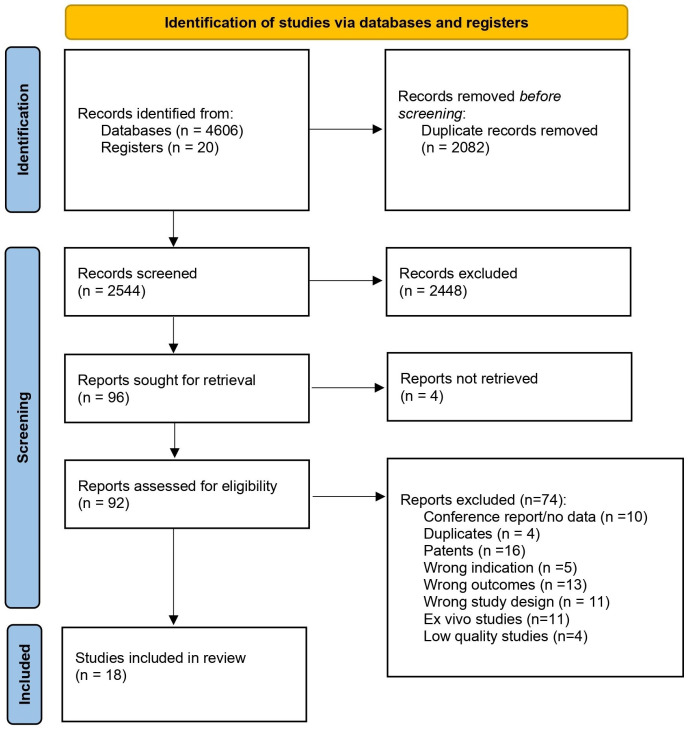
Study selection flow diagram.

Our initial search identified 4626 reports, which were then screened for duplicates resulting in the removal of 2082 reports. The remaining 2544 reports were assessed through title and abstract screening, and 2448 were subsequently excluded. A full-text review was conducted on 92 studies, of which 18 studies met the final inclusion criteria and were included in the systematic review (see Figure 1 for a detailed flowchart of the study selection).

### 3.2. General Characteristics of the Selected Studies

**Figure 2:**
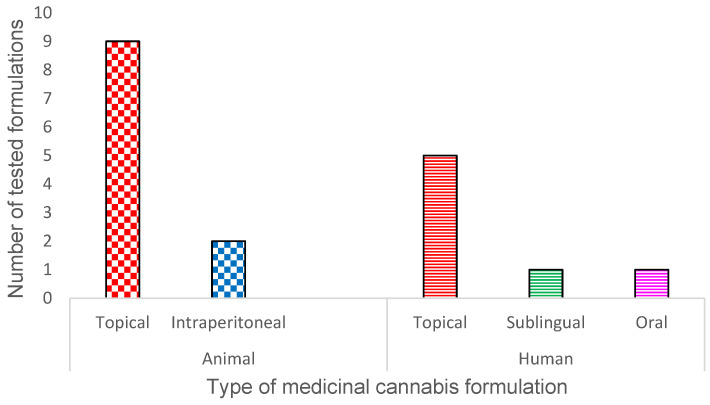
Different types of medicinal cannabis formulations tested in animal and human studies.

The included 18 studies were peer-reviewed and published between 2016 and 2024. Among these, eleven were animal studies [67,131,132,133,134,135,136,137,138,139,140] investigating the effects of *C. sativa* extracts and cannabinoids on wound healing and antibacterial activity. These studies utilized murine (*n* = 10) and equine (*n* = 1) models, encompassing 437 animals across various study groups. The remaining seven were human case studies, case series, open-label trials and randomized controlled trials [141,142,143,144,145,146,147], involving 92 participants from Canada, the United States, Thailand, and the Netherlands. The sample sizes ranged from 1 to 69 patients, with ages spanning from 6 months to 90 years. Chronic wound duration in human participants ranged from 6 months to 20 years, whereas recurrent aphthous ulcers had durations of 48 h or less. The routes of administration involved topical and intraperitoneal for animals and oral, sublingual, and topical for humans. Figure 2 summarises the different types of MC formulations tested in both animal and human studies.

### 3.3. Quality of the Selected Studies

All the in vivo studies (*n* = 11) [67,131,132,133,134,135,136,137,138,139,140] scored one on the RoB assessment, indicating a low risk of bias. These studies provided detailed descriptions of their design. In contrast, the human studies (*n* = 7) scored between 5 and 10 on the RoB assessment. Five of these studies were evaluated using the JBI Critical Appraisal Checklist for Case Reports [141,142,143,144,146], and all adequately described the clinical condition, diagnostic tests, and post-treatment clinical status [141,142,143,144,146]. The remaining studies were assessed using the JBI Critical Appraisal Checklist for Case Series [145] and JBI Critical Appraisal Tool for Assessment of Risk of Bias for Randomized Controlled Trials [147]. Due to the limited number of available studies, all human studies were included in the data analysis regardless of their quality scores to ensure a comprehensive evaluation of the existing evidence.

### 3.4. In Vivo Studies

The results of the animal studies are presented in Table 1 and Table 2. Table 1 summarises the descriptive characteristics of the in vivo studies, while Table 2 details the formulation characteristics of the interventions, including the routes of administration.

Among the eleven included animal studies, seven examined effects of MC on acute wounds [67,131,132,133,134,137,138], three on acute infections [135,136,139], one on chronic wound infection [139], and another study on cutaneous lupus erythematosus [140]. A topical formulation containing 12% *v/v* of fixed oil obtained from hemp (*C. sativa* L.) fruit incorporated with other fixed oils (sesame seed, wild pistachio fruit, and walnut seed) demonstrated significant wound healing when applied to third-degree burns in a murine model compared to both silver sulfadiazine and negative control (*p* < 0.001) [131]. Anandamide, an endocannabinoid, reduced lesions caused by cutaneous lupus erythematosus both prophylactically and as a treatment when applied topically in a murine model [140]. Although two studies [132,133] found no difference between CBD treatments and their respective vehicle controls, one study [139] showed a reduction in relative wound size with CBD compared to the vehicle control in animal wound healing models. When CBD was incorporated into nanoparticles for treating infected or uninfected acute wounds, wound size was significantly reduced compared to other groups [138,139]. Additionally, CBD incorporated into nanoparticles combined with laser therapy significantly reduced wound size in infected chronic wounds [139]. Synthetic cannabinoids (JWH133 and GP1a), acting as CB2R agonists, were tested in murine models of acute wounds either topically or intraperitoneally, resulting in faster re-epithelialisation (*p* < 0.05 compared to the vehicle control), though this was sometimes not statistically significant [67,134,137,148]. These findings suggest that endocannabinoids, phytocannabinoids, and synthetic cannabinoids possess varying degrees of wound healing ability. 

Topical CBD resulted in a significant reduction in *S. aureus* load compared to vehicle control after 48 h in acute skin infections in a murine model (*p* < 0.05) [135]. Furthermore, topical CBD reduced MRSA load compared to the control after 48 h in infections associated with both acute and chronic wounds in murine models [139]. CBD-loaded nanoparticles combined with laser treatment reduced MRSA load to minimum levels at 48 h in infections associated with both acute and chronic wounds in murine models compared to controls (*p* < 0.001) [139]. Tetrahydrocannabidiol, a semi-synthetic cannabinoid, reduced MRSA load in a murine skin infection model, with results comparable to 2% mupirocin by day 5 [136]. These findings confirm the role of cannabinoids in treating staphylococcal skin infections.

In summary, various MC products, such as hemp fruit oil extract, anandamide, CBD, GP1a, and JWH133 have shown promising effects on wound healing in animal models [67,131,134,137,138,139,140]. These studies reported faster re-epithelialisation, a reduction in wound size, accelerated wound healing rates (66% by day 10 and 99.5% by day 21, 0.1 cm^2^/day), and complete wound healing within 5–84 days [131,133,137,139]. In the inflammatory phase of wound healing, CBD demonstrated a significantly reduced inflammatory score compared to the vehicle control [132]. Additionally, treatment with MC prominently modulated various processes essential for effective wound repair, including angiogenesis, fibroplasia, granulation tissue formation, collagenization, hyperkeratinization, and hair follicle structure formation [67,131,132,137,138]. Notably, the rate of wound healing improved significantly in the middle stages of healing, particularly from day 3 to day 10 [67,131,138]. However, CBD did not show significant effects on the wound size or healing time in some test groups, yet, when incorporated into nanoparticles, CBD exhibited promising effects on wound healing. Topical CBD, either alone or as CBD-loaded nanoparticles and tetrahydrocannabidoil, reduced the bacterial load in skin infections [135,136,139]. Overall, no adverse reactions were reported in any animal studies except for the increase in white blood cell count by CBD on day 11 in a murine model [139].

### 3.5. Human Studies

The results of human studies are summarised in Table 3 and Table 4. Table 3 presents the descriptive characteristics of the included studies, while Table 4 details the formulation characteristics and routes of administration. 

MC was administered orally, sublingually, or applied directly to the wounds. The administration of MC, either alone or in combination was associated with decreased blistering, reduced ulcer size, shortened wound healing time, closure of non-healing wounds, and the alleviation of symptoms such as pain and pruritus [141,142,143,144,145,146,147]. However, two studies [143,144] did not report effects of MC on wound closure but noted symptom alleviation. Human studies revealed that patients, including children, with chronic recalcitrant wounds unresponsive to other treatments, experienced wound healing following the administration of oral MC oil, topical CBD oil (exact concentrations of components were not available), or other cannabinoid-containing topical preparations (3.8 mg/mL CBD, <1 mg/mL THC, 31.3 mg/mL quercetin, 25.3 mg/mL diosmin, 2.5 mg/mL hesperidin, and 152.7 mg/mL β-caryophyllene [BCP]).

MC has been indicated for chronic, non-responding wounds associated with rare conditions such as epidermolysis bullosa (EB), non-uremic calciphylaxis leg ulcers, and pyoderma gangrenosum; and also with non-healing leg ulcers, pressure ulcers, and recurrent aphthous ulcers [145,146,147]. Administration of MC has been shown to reduce pain scores, decrease the need for additional analgesics, and alleviate pruritus in patients suffering from pressure ulcers, EB, non-uremic calciphylaxis leg ulcers, pyoderma gangrenosum, and recurrent ulcers [141,142,143,144,146,147]. Wound closure, reduction in ulcer size, and decreased blistering were achieved by oral MC oils, topical CBD paste, topical CBD oil, and topical formulations containing cannabinoids as well as other phyto-actives found in the *C. sativa* plant [141,142,145,146,147]. The latter formulations included higher amounts of BCP, a terpene in *C. sativa* [142,145]. Formulations that resulted in wound healing and relieved wound-related symptoms mainly contained CBD and THC in oil-based formulations or in formulations containing terpenes [141,142,143,144,145,146,149].

In summary, the studies reviewed consistently demonstrate that MC application to chronic, non-healing, and recurrent wounds in humans produces beneficial outcomes.. Specifically, MC administration resulted in significant pain reduction, both clinically and statistically (*n* = six studies, 81 patients) [141,142,143,144,146,147]; decreased ulcer size (*n* = one study, 69 patients) [147]; decreased blistering (*n* = one study, three patients) [141]; reduced healing time (*n* = two studies, 72 patients) [141,147]; a reduction in pruritus (*n* = one study, three patients) [143]; and resulted in complete wound closure after an average time of 54 days (range: 21–150 days; *n* = three studies, 17 patients) [142,145,146]. The reported wound healing rate was 3.3 cm^2^/30 days for venous leg ulcers (*n* = one study, 14 patients) [145] and 1.5–1.8%/day for non-uremic calciphylaxis leg ulcers (*n* = one study, two patients) [142]. Except for Maida and Corban, 2017 [144], and Umpreecha et al. 2023 [147], the statistical significance of these results has not been reported. MC treatment facilitated a characteristic two-phase wound healing process in cases of chronic non-uremic calciphylaxis leg ulcers. The initial phase was dominated by pronounced granulation, followed by significant re-epithelialisation in the latter half [142]. Apart from Umpreecha et al. 2023 [147], other human studies did not use either standard therapy or placebo for comparison. No significant side effects except increased appetite were reported [143].

## 4. Discussion

To the best of our knowledge, this systematic review represents the first comprehensive evaluation of the integumentary wound healing and antibacterial potential of MC. Previous narrative reviews have highlighted the promising role of cannabinoids in wound management [61,64,65,109,110,112]. Additionally, three reviews have summarized the therapeutic potential of CBD, including its efficacy in wound healing [62,113,114]. The narrative reviews summarising the antimicrobial potential of cannabinoids did not specifically address their effects on integumentary infections [47,56,113,116,117]. Current research on MC has explored its potential use in acute wounds such as burns and incisions, skin infections in acute and chronic wounds in animal models, and chronic wounds in humans, including conditions such as EB, venous leg ulcers, pyoderma gangrenosum, pressure ulcers, and non-uremic calciphylaxis. It has also been studied in recurrent aphthous ulcers in humans and cutaneous lupus erythematosus in mice. The evidence suggests that MC may hold promise for certain wound types, as demonstrated by improvements in symptoms such as inflammation, pain, pruritus, and overall wound healing. Also, it improved the sleep quality and reduced anxiety. However, the current evidence is based on a limited number of small and primarily low-quality studies, necessitating further well-designed research to better understand the potential of MC in wound healing.

### 4.1. In Vivo Studies

The ECS is present in all animal species except insects [150,151,152], making animal and ex vivo studies valuable for preclinical observations of the therapeutic effects of MC. Murine and equine models have been used to test the wound healing and antibacterial properties of MC, specifically in acute wounds or infection scenarios such as burns, oral ulcers, skin incisions, and skin infections [67,131,132,133,134,135,136,137,138,139,140]. It has also been studied in cutaneous lupus erythematosus, both prophylactically and as an intervention [140]. The included animal studies utilized negative and positive controls, with positive controls including gold standard agents such as silver sulfadiazine for burns, mupirocin for infections, and Tegaderm wound dressings [131,135,136,138]. Negative controls included untreated groups, vehicle controls, or normal saline. All animal studies in this systematic review were found to be reliable according to the ToxRTool category one without restrictions. However, heterogeneity in the assay types, and varied bioactive MC and their formulations precluded a pooled analysis.

#### 4.1.1. Major Effects on Wounds

Significant wound healing properties of MC were reported on murine third-degree burns treated with topical *C. sativa* fruit oil with other medicinal oils, achieving complete wound healing in 21 days [131]. Synthetic cannabinoids (GP1a and JWH133) enhanced re-epithelialisation in murine dermal wounds. The formulation and route of administration influenced complete wound closure, with topical GP1a gel closing wounds in 5–7 days, while intraperitoneal GP1a injection resulted in complete re-epithelialisation in 13 days [67,137]. Intraperitoneal CBD in a murine model [132] and topical CBD in UMF 5 manuka honey in an equine model [133] showed similar wound healing effects to their respective vehicle controls. CBD-containing alginate-Zn hydrogel showed faster wound healing compared to vehicle, negative, and commercial controls on day seven and to the negative control on day 14 (~95% wound closure). Reported uninfected wound healing rates included a 66% wound size reduction on day 10 for murine third-degree burns, an 86% wound size reduction on day 10 for murine full-thickness dermal wounds, and a 0.1 cm^2^/day for second-intention wound healing in an equine model [131,133,138]. In general, third-degree burns or surgical uninfected wounds healed by secondary intention were completely healed in 23 (range: 5–84) days with a 66–86% wound healing rate in 10 days when treated with MC. Compared to other investigational agents, MC offers competitive healing times for burns and wound healing by secondary intention. For example, silver sulfadiazine ointment (10–23 days), *Aloe vera* ointment (10 days), and 5% *Albizia julibressin* bark extract (8 days) also demonstrate similar or faster healing times in burn wound healing [153,154,155,156]. For wounds healing by secondary intention, healing times vary widely across different interventions (26–104 days), with MC providing a comparatively faster and uncomplicated option for wound healing [157,158,159,160].

In murine models, MC exhibited bacterial load reduction and wound healing in either *S. aureus* or MRSA-infected wounds [135,136,139]. Significant bacterial load reduction was observed within 48 h, reaching minimal levels by day 5, which is comparable to 2% mupirocin, a standard treatment for staphylococcal skin infections [135,136,139,161,162]. Treatment with CBD alone or incorporated in nanoparticles healed >75% of acute and chronic infected wounds by day nine and day eleven, respectively [139]. CBD incorporated into nanoparticles, combined with laser therapy, almost wholly healed infected chronic wounds by day 11 in the murine model [139]. MC has also proven effective against other bacteria causing skin infections, such as *Streptococcus pyogenes* and clinical strains of *S. aureus*, within in vitro studies [48,50,52,163,164,165,166,167,168,169,170,171,172,173,174].

Anandamide showed improvement in cutaneous lupus erythematosus in murine models [140]. Cutaneous lupus erythematosus lesions are typically treated with topical steroids and calcineurin inhibitors (tacrolimus and pimecrolimus) but these treatments have limitations due to adverse effects [175,176,177]. Anandamide demonstrated improvement in lesions within 21 days, outperforming the typical 28–56 days required for tacrolimus treatment [140,175]. These findings suggest that further investigation into MC for this condition is warranted.

Some in vivo studies showed no difference between CBD and vehicle controls regarding acute wound healing, attributed to factors such as administration route, formulation-related issues, or sub-therapeutic concentrations of active ingredients [132,133,178,179]. For instance, systemic administration of CBD at higher doses (100–250 mg/kg) was ineffective in treating a MRSA thigh infection in mice [135]. Compared to these, the intraperitoneal CBD dose (5 or 10 mg/kg) used by Klein et al. 2018 [132] to treat wounds might be sub-therapeutic. Moreover, positive controls used in the included studies exhibited different wound healing rates compared to other studies on similar animal models, while some studies lack a positive control group [131,132,133,156,180,181,182]. In two studies, each animal served as its own experimental control and the therapy might have combined effects, either synergistic or antagonistic, due to systemic absorption of active ingredients through the wound bed [133,137]. This is supported by the ability of manuka honey as well as cannabinoids to exert synergistic and antagonistic effects [183,184,185,186,187]. In addition, other ingredients present in the tested MC formulations, such as *Sesamum indicum* oil, *Pistacia atlantica* oil, olive oil, manuka honey, alginate, zinc, alginate hydrogels with zinc nanoparticles, chitosan hydrogels, Prussian blue nanozyme, chitosan-Prussian blue nanoparticles (with or without laser light), silica nanoparticles, coconut oil, and coconut oil-loaded silica nanoemulsion also possess wound healing and antimicrobial properties [131,180,181,182,188,189,190,191,192,193,194,195,196,197,198,199,200,201,202,203,204,205,206,207,208,209,210]. These highlight the need for consistent and standardised studies for accurate comparison of therapeutic efficacy.

#### 4.1.2. Other Effects on Wounds

Additional effects of MC reported by in vivo studies include anti-staphylococcal effects, anti-inflammatory effects, increased angiogenesis, modulation of fibroblast counts, higher re-epithelialisation, amelioration of epidermal hypertrophy, decreased skin thickness, decreased fibrosis area, regeneration of hair follicle structures, and reduced scar formation on acute wounds [67,131,132,134,135,136,137,138,139]. Notably, some MC formulations demonstrated complete healing without scar formation, an advantage over natural healing and treatments like silver sulfadiazine, which often result in scarring [131,211,212,213,214,215,216].

#### 4.1.3. Side Effects

No significant side effects were reported with intraperitoneal injection of CBD, suggesting greater tolerability [132]. However, a significant increase in white blood cell count on day 11 was reported with topical CBD [139], while other studies did not record side effects. Further research is needed to fully assess the safety and tolerability of MC formulations for wound healing purposes.

In summary, *C. sativa* extracts (fixed oil from *C. sativa* fruit), endocannabinoids (anandamide), phytocannabinoids (CBD), semi-synthetic cannabinoids (tetrahydrocannabidiol), and synthetic cannabinoids (JWH133, Gp1a) were studied for antibacterial and/or wound healing potential in preclinical studies. These revealed substantial wound healing potential by fixed oil from *C. sativa* fruit with other oils, Gp1a, and CBD, with complete wound healing achieved within 23 (range: 5–84) days and 66–86% wound healing in 10 days. Antibacterial effects were exerted by CBD and tetrahydrocannabidiol. MC also offers additional benefits, including anti-staphylococcal properties, anti-inflammatory effects, increased angiogenesis, modulation of fibroblast proliferation, amelioration of epidermal hypertrophy, regeneration of hair follicle structures, and higher re-epithelialisation.

### 4.2. Human Studies

MC has been investigated for its potential to heal chronic, non-healing wounds in humans resulting from various conditions, including EB, non-uremic calciphylaxis leg ulcers, pyoderma gangrenosum, pressure ulcers, and venous leg ulcers, as well as recurrent aphthous ulcers. Except for the randomized controlled trial with CBD paste on recurrent aphthous ulcers [147], none of the other studies included a control group for comparison. The subjects in these studies spanned different age groups, including paediatric, middle-aged, and geriatric patients. Due to the heterogeneity of the wound types and reported outcomes, it was extremely challenging to perform a pooled analysis.

#### 4.2.1. Major Effects on Wounds

Complete wound healing with MC in humans was reported in patients with non-uremic calciphylaxis leg ulcers, venous leg ulcers, and pressure ulcers. The mean time to complete wound healing ranged from 34 days in venous leg ulcers to approximately 60 days in pressure ulcers and 76 days in non-uremic calciphylaxis leg ulcers [142,145,146]. Generally, the time to heal chronic wounds in humans was 54 days (range: 14–150 days). The reported healing rate was 3.3 cm^2^/30 days for venous leg ulcers [145] and 1.5–1.8%/day for non-uremic calciphylaxis leg ulcers [142]. Self-administration of MC in paediatric patients resulted in decreased blistering and healing times [141].

Similar studies in patients with non-uremic calciphylaxis reported complete wound healing in 6–9 months after intravenous sodium thiosulfate, four months after intravenous sodium thiosulfate with oral cinacalcet, and 2–6 months after pamidronate infusion [217,218,219,220,221]. Complete wound healing for venous leg ulcers was reported in four months for oral Daflon^®^, four months for oral pentoxifylline, and 28 days for negative-pressure wound therapy [222,223,224,225,226]. Pressure ulcers are typically treated with air-fluidized beds, alternating-pressure surfaces, nutritional supplements, different dressings (alginate, foam, gauze, honey, hydrogel, hydrocolloid, silver, and film), negative-pressure wound therapy, electrical stimulation, platelet-derived growth factor, and light therapy [227,228,229]. Due to the complex nature of pressure ulcers, time-to-heal data are scarce in the literature [229,230,231,232]. The reported time to heal for recurrent aphthous ulcers varies depending on the treatment such as AMD laser treatment (2–4 days), CO_2_ laser treatment (3–7 days), curcumin (3–5 days), doxycycline (2–4 days), and triamcinolone acetonide (3–5 days) [233,234,235]. However, long-term follow-up for recurrent aphthous ulcers is needed to observe recurrence. Compared to most of these treatments, MC formulations offer the advantage of healing within a relatively shorter duration (1–2.5 months) [142,145,146]. However, it is essential to consider that the studies examining the MC efficacy in wound healing also incorporated compression therapy and foam dressings, independently recognized wound healing treatments [142,145,146,236,237,238].

Various wound healing rates reported in the literature with different agents and methods offer some context. These include 0.08 cm^2^/day by pulsed radiofrequency energy treatment; 0.1 cm^2^/day by negative-pressure wound therapy; 0.29 cm^2^/day by manual lymph drainage plus compression bandaging; 0.83 cm^2^/day by topical hyperbaric oxygen therapy; 0.89 cm^2^/day by compression bandaging; and 0.56 cm^2^/month by compression and advanced wound dressing [236,239,240,241,242]. Compared to these, the healing rate associated with MC is lower. MC shows promise, whereas traditional interventions did not achieve complete healing in all patients [224,236].

Evidence from case reports indicates that topical application of MC (CBD oil) reduced blistering and pain in three paediatric EB patients [141], and sublingual MC (oil containing *C. sativa* plant extract in refined arachis oil) alleviated symptoms associated with EB wounds in three adults [143,149]. The application of CBD oil was self-initiated in paediatric patients, and the exact composition of the product was not reported [141]. Notably, in some EB patients, blistering naturally decreases with age [243,244], making the precise role of MC in reducing skin blistering unclear.. An international cross-sectional survey [245] revealed the use of topical and oral preparations containing THC or CBD alone, or their combinations for managing EB wounds and associated symptoms. Currently, a phase II clinical trial that examines the safety and preliminary efficacy of cannabinol (CBN) cream on wounds and affected skin areas in patients with EB is being completed [246]. However, the phase II/III trials registered to determine the efficacy and safety of a 3% CBD cream for acute and chronic wounds in EB patients were withdrawn since major changes in the study protocol have been recommended during the reviewing process [247]. Sublingual oil (100 mg/mL THC and 50 mg/mL CBD) for EB patients has been designed in a randomised, placebo-controlled, double-blind trial to provide more robust evidence [248]. A phase I clinical trial on human acute wound healing on the forehead with CBD oil plus silicone ointment is ongoing [249]. Outcomes from these studies will provide further insight into the wound healing capability of MC and its clinical potential.

#### 4.2.2. Other Effects on Wounds

The administration of MC has been associated with alleviating wound-related symptoms such as pain reduction (evident by lowered pain scores, decreased need for additional analgesics), improved ambulation, and reduced pruritus [141,142,143,144,145,146,147]. Furthermore, there were no reports of hypertrophic scars or keloids development, and patients experienced improved sleep quality, reduced anxiety, and enhanced quality of life [141,142,143,144,145,146,147]. Apart from an increased appetite observed with sublingual MC oil [143], no significant side effects were reported with topical MC preparations and oral paste [141,142,145,147]. The analgesic effects of MC, particularly in 1:1 THC: CBD preparations, have been well documented [250,251,252,253,254]. Conversely, treatments with THC alone did not demonstrate significant pain relief [254].

In summary, MC was associated with the complete healing of chronic, non-healing wounds and the alleviation of symptoms such as pain and pruritus without leading to scar formation. The MC formulations studied in humans included THC- and CBD-containing oils, CBD paste, CBD oil, whole *C. sativa* plant extract, and a formulation containing a mixture of compounds present in *C. sativa.*

Notably, the efficacy of CBD was found to be highly dependent on the specific formulation used in in vivo studies. This was evident in ex vivo studies, where different formulations exhibited varying effectiveness [135]. For a clearer understanding of the impact of these formulations, we have summarised the types of formulations employed in the included studies in Table 2 and Table 4. In most cases, MC was incorporated into other natural oils such as olive, emu, sunflower, and arachis or in a mixture of fixed oils such as sesame, wild pistachio, and walnut. Others were formulated in topical gels, pastes, sprays, tinctures, and creams. Two studies employed hyaluronic acid plus an *Aloe vera* gel base for the wound bed and a liposomal base for the peri-wound tissues with the same active pharmaceutical ingredients [142,145]. Hyaluronic acid and *Aloe vera* gel were combined to promote absorption through lipophilic tissues, while the liposomal base enhanced drug absorption through the stratum corneum [145]. Promising topical formulations and drug delivery systems for wound healing include coacervates, complexes, conjugates, creams, films, gels, hydrogels, nano-drug delivery systems, ointments, scaffolds, sponges, and wafers [255,256,257,258,259,260]. Future studies should focus on optimizing MC formulation bases and dermal carrier systems while considering the physiochemical properties of MC to improve therapeutic outcomes [261,262].

The quality scores of the human studies ranged from five to seven on the JBI Critical Appraisal Tools, with eight being the highest score for studies except for the randomized controlled trials. Umpreecha et al. 2023 [147], the only included randomized controlled trial scored 10, with 13 being the highest. In the included studies, except for the randomized controlled trial, treatment outcomes were not consistently defined and measured accurately, and different time points were used for the patients within the same study. Additionally, wound-related symptoms such as pain, pruritus, debridement, inflammation, and odour [263] were not always reported, likely due to poor study design in open-label trials and case studies. The absence of placebo- or vehicle-controlled study designs in most included studies may have underestimated the effects of other components present in the study formulations.

While MC has shown promise for chronic, non-healing wounds resulting from various conditions in humans, the heterogeneity of wound types and outcomes prevented a pooled analysis. Notably, studies have yet to test MC on skin infections in humans, and the results of animal studies cannot be directly translated to humans [264]. Hence, further studies are needed to understand the wound healing properties of MC, particularly in different types of wounds and skin infections.

Although all studies targeted antibacterial or wound healing properties, the different endpoints and measurements limited our understanding of MC use in individually targeted wounds. Furthermore, details of MC, such as the types and concentrations of cannabinoids present in the interventions, were not always reported, potentially affecting the reproducibility of these findings. Nevertheless, MC’s promising antibacterial and wound healing properties make it a strong candidate for superficial bacterial skin and skin structure infections and wounds. Additionally, other therapeutic attributes of cannabinoids, such as anti-pruritic and anti-inflammatory properties, may improve the therapeutic success of MC.

## 5. Conclusions

This systematic review methodically evaluates the current evidence on the wound healing and antibacterial properties of medicinal cannabis (MC) in treating integumentary wounds and infections, whether used alone or in combination with other agents. The findings demonstrate that MC possesses significant antibacterial properties and promotes wound healing, showing promising results in both animal models and human studies. Hemp fruit oil extract, CBD, and GP1a resulted in complete healing in acute wounds in 23 (5–84) days with a 66–86% healing rate in 10 days in animal models. CBD and tetrahydrocannabidiol irradicated *Staphylococcus aureus* and MRSA in skin infections of animal models. MC oils quantified for THC and CBD content, CBD-rich oil, and gels with THC, CBD, and terpenes promoted healing of chronic, non-healing wounds in humans with complete wound closure in 54 (21–150) days. In human subjects, the use of cannabinoids led to reduced blistering, shortened healing times, and alleviated symptoms, thus improving quality of life through topical, oral, and sublingual routes. The observed side effects were minimal, with increased appetite from sublingual oil in humans and increases in white blood cell count by CBD in animals being the most common.

However, the absence of controlled clinical trials and the inconsistent reporting of outcomes across studies significantly limit our ability to draw definitive conclusions regarding the specific effects of cannabinoids on different wound healing phases. Therefore, further robust, controlled studies are necessary to fully understand the therapeutic potential of cannabinoids in treating superficial bacterial skin and skin structure infections as well as dermal wounds.

## Figures and Tables

**Table 1:** Characteristics of in vivo studies (*n* = 11) included in laboratory and field settings.

Study Setting and Location	Study Design	Method/Assay	Intervention	Outcome Measure(s)	Treatment Outcome(s)	Quality Score
Mehrabani et al. 2016 [131] In vivo, laboratory-reared	Experimental	Third-degree burns in mice. **Experimental group** 25% of the total body surface area of the male albino mice (*n* = 8 per group, 6–8 weeks old) was burnt by boiling water.	**Treatment** Formulation (100 mg/mouse) containing a combination of the oils of sesame (*Sesamum indicum* L.) seed (60%), wild pistachio (*Pistacia atlantica* Desf.) fruit (20%), hemp (*Cannabis sativa* L.) fruit (12%), and walnut (*Juglans regia* L.) seed (8%) was applied topically twice daily for 21 days. **Comparator** Topical SSD * twice daily (200 mg/kg/day, as positive control), Untreated group (negative control)	Wound area, rate of wound healing, epithelialisation time, percentage of wound contraction.	Significant wound contraction was observed with the formulation on days 10 (65.9 ± 3.8%), 14, 18, and 21 (99.5 ± 0.8%) compared to SSD and untreated groups (*p* < 0.001). Epithelialisation time was significantly decreased by the formulation (formulation = 20.5 ± 1.37 days, untreated group 25.5 ± 0.83 days) (*p* < 0.001). The formulation resulted in improved skin re-epithelialisation, granulation tissue formation, scattered inflammatory cells infiltration, and collagenization on day 21. The collagenization and epithelialisation were significantly higher in the formulation group compared to SSD * group on day 21 (*p*-value is not given). No scar formation.	1
Wang et al. 2016 [67] In vivo, laboratory-reared	Experimental	Full-thickness skin wound in mice. **Experimental group** Two full-thickness excisions (6 mm diameter) were made symmetrically on the dorsal skin of 8-week-old male BALB/c mice (20–25 g). After the surgery, mice received daily treatments and were euthanized at 0.5, 1, 3, 5, 7, 10, 13, 17, and 21 days post-injury (6 mice at each time point).	**Treatment** Daily intraperitoneal injections of Gp1a (3 mg/kg, dissolved in vehicle). **Comparator** Daily intraperitoneal injections of vehicle (5% dimethyl sulfoxide/2% Tween-80/physiological saline, 2.5 μL/g), or AM630 (3 mg/kg, dissolved in vehicle). Six mice without surgery were used as controls.	Percentage wound size, wound re-epithelialisation percentage.	GP1a inhibited wound contraction after decrustation. Wound sizes in GP1a group were significantly larger than in the vehicle group on days 10–21 post-injury (*p* < 0.05). No significant difference in wound size was observed between AM630 and vehicle group. Wound re-epithelialisation was accelerated in GP1a group compared to other two groups (*p* < 0.05). Complete re-epithelialisation was reported in all 3 groups by day 13. In AM630 group, wound re-epithelialisation was delayed compared to vehicle control on days 5–7 (*p* < 0.05). The dermal scar in the GP1a group was thinner and the collagen fibres were much slenderer. AM630 group showed similar epidermal and dermal morphologies with vehicle group.	1
Klein et al. 2018 [132] In vivo, laboratory-reared	Experimental	Oral wound healing in rats. **Experimental group** Male Wistar rats (*Rattus novergicus*) (~ 90 days old, ~ 300 g, *n* = 10 per group) with an induced ulcer on the ventral tongue.	**Treatment** Intraperitoneal injection of CBD ** (5 or 10 mg/kg body weight) was initiated immediately after ulcer induction and repeated daily for 3 or 7 days. **Comparator** Vehicle (2% Tween 80 in saline) for 3 or 7 days.	Wound area, presence of oedema, hyperaemia and inflammatory infiltrate (by histological examination).	Wound size was not significantly different from that of the respective control group on any of the test dates. Significantly lower inflammation was observed by histological examination in CBD treatment groups compared to the control group (*p* < 0.05) on day 3. A significant difference in inflammation was not observed within the groups on day seven by histological examination. Higher re-epithelialisation (angiogenesis, fibroplasia, collagen fibres deposited in a well-arranged manner, thicker marginal epithelium, hyperkeratinisation) was observed in CBD-treated groups by histological examination on day seven. These histological changes were not sufficient to provide clinical improvement in the wound. No side effects were observed.	1
Koyama et al. 2019 [134] In vivo, laboratory-reared	Experimental	Full-thickness skin wound in mice. **Experimental group** Full-thickness excisions (< 5 mm × 5 mm) were made on the backs of 8–10-week-old adult female C57BL/6J mice and a silicone ring was attached to the lesion by a liquid sealing bandage (serving as a reservoir for the treatment). They were treated daily. On day five following the surgery, the skin was harvested and stained with a K14 (a marker of re-epithelialisation) antibody.	**Treatment** Topical 1 μM JWH133 (CB2R agonist, 50 μL) in olive oil daily (*n* = 13) for five days **Comparator** Topical olive oil (vehicle) daily (*n* = 16).	Distance of cell migration from the wound edge.	JWH133 enhanced re-epithelialisation compared to the vehicle control group (*p* = 0.071).	1
McIver et al. 2020 [133] In vivo, field, contaminated wound	Experimental	The second intention wound healing in the equine model. **Experimental group** 6 adult Standardbred horses (3 geldings, 3 mares, 3–10 years old) with five 2.5 cm × 2.5 cm full-thickness skin wounds on the dorsomedial aspect of the metacarpi (three wounds on a forelimb, two wounds on the contralateral forelimb) contaminated with faeces for 24 h under a bandage.	**Treatment** Topical application of 1% CBD ** in UMF *** 5 manuka honey (2 mL) daily for 42 days. Wounds were bandaged daily in the first 13 days. **Comparator** UMF 5 manuka honey (vehicle, 2 mL), UMF 20 manuka honey (2 mL), or 0.9% sterile saline (2 mL) daily for 42 days.	Wound area, overall time to complete healing, overall rate of wound healing (cm^2^/day).	Total days for healing (CBD: 84 days, other groups: 83–88 days) and overall healing rates (0.1 cm^2^/day) were similar across all treatment groups.	1
Blaskovich et al. 2021 [135] In vivo, laboratory-reared	Experimental	Bioluminescent in vivo mouse skin infection model for acute wounds. **Experimental group** Skin of the back was shaved and disrupted in immunocompromised adult female CD1 mice (*n* = 6 per group) and was inoculated with 10 μL *Staphylococcus aureus* Xen-29 bioluminescent bacteria (5 × 10^7^ CFU ****).	**Treatment** Formulations of 5% CBD formulation (either BTX 1503, 1503 gel, or BTX 1204 gel, 50 μL) topically at 0 (initiated immediately after inoculation), 12, 24, and 32 h post-infection. **Comparator** 2% mupirocin (50 μL) or vehicle (50 μL).	48 h after the first treatment and infection, animals were sacrificed, and the skin assessed for signs of gross pathology, CFU’s per mice. Bioluminescent in vivo imaging was performed pre-infection and then at 4, 24, 36, and 48 h post-infection.	5% CBD significantly reduced *S. aureus* load at 48 h compared to vehicle (*p* = 0.0184). 2% mupirocin was more effective than 5% CBD formulations tested (I = <0.0001 for mupirocin vs. vehicle).	1
Zhao et al. 2021 [137] In vivo, laboratory-reared	Experimental	Full-thickness skin wound in mice. **Experimental group** 3M transparent ventilation tape was placed around the operative area of 6-week-old (18–22 g) male wild-type BALB/c mice. Two full-thickness circular punch wounds (4 mm diameter) were made symmetrically over the midline of the dorsum. Then, the wounds in each mouse were treated: one with Gp1a-gel and the other with scramble Tm hydrogel (vehicle). Six mice were sacrificed from each group at 4, 8, 12, and 20 days post-surgery.	**Treatment** 50 μL of 4 mg/mL Gp1a-gel applied once after the surgery. **Comparator ** 50 μL scramble Tm hydrogel (vehicle) applied once after the surgery.	Length of epithelial sheet, time to complete wound healing.	GP1a gel resulted in a significantly longer epithelial sheet compared to vehicle from days 4 to 6 post-surgery (*p* < 0.05). Complete wound healing was achieved in 5–7 days, with earlier wound closure observed with GP1a gel compared to the vehicle in the same mouse.	1
Zheng et al. 2022 [138] In vivo, laboratory-reared	Experimental	Full-thickness skin wound in rats. **Experimental group ** Two full-thickness circular punch wounds (10 mm diameter) were made on the dorsal region of the 8–10-week-old (200–250 g) male Sprague Dawley rats. Four different treatments were, respectively, applied to the wounds (*n* = 6 per group) and the wound beds were covered with medical gauze. The state of wound healing was recorded on days 0, 3, 7, 10, and 14 post-surgery.	**Treatment** 2% *w/v* CBD/Alg@Zn (cannabidiol containing alginate–zinc hydrogel) applied once after the surgery. **Comparator** Normal saline group (control), Alg@Zn group (or alginate-Zn hydrogel, vehicle), or Tegaderm™ group (or 3M, commercial wound dressing) applied once after the surgery.	Percentage of remaining wound area.	Significant reduction in wound size on day 7 by CBD/Alg@Zn compared to other treatments (*p* < 0.05). CBD/Alg@Zn showed faster wound healing compared to other groups although not statistically significant. Percentages of remaining wound area on day 10 were 34.3 ± 2.9 for the control group, 25.8 ± 2.0 for the 3M group, 17.1 ± 2.1 for the Alg@Zn group, and 13.7 ± 2.7 for the CBD/Alg@Zn group. Wounds were not completely healed on day 14 in any of the groups. On day 14, CBD/Alg@Zn had the lowest remaining wound area compared to other groups, although not statistically significant. Angiogenesis and hair follicle structures were observed with CBD/Alg@Zn on day 14.	1
Zhong et al. 2022 [139] In vivo, laboratory-reared	Experimental	**Experiment A:** in vivo mouse skin infection model for acute wounds. **Experimental group** The skin of the back of 6-week-old female BALB/c mice (*n* = 5 per group) was shaved, and circular wound (~10 mm) was made. Methicillin-resistant *Staphylococcus aureus* (MRSA) cells (10^8^ CFU ****) were coated on wounds, and continuously infected for two days. Medicine-free band-aids were used to fix the MRSA and nanoparticles on wounds. Wounds were treated every two days. On the second day, liquid on wound site was absorbed with a sterile cotton swab and plated on a culture dish.	**Treatment** 100 μL of CBD (10 μg/mL), or 100 μL of Chi@HMPB@CBD NPs (CBD-loaded hollow mesoporous Prussian blue nanoparticles coated with chitosan; containing 100 μg/mL HMPB NPs) topically every two days up to day 7. Prior to fixing the wounds, Chi@HMPB@CBD plus laser groups were irradiated with an 808 nm laser (1 W/cm^2^, 3 min) and treated with 100 μL of Chi@HMPB@CBD topically every two days up to 7 days. **Comparator ** 100 μL of PBS (control), or 100 μL of Chi@HMPB NPs (hollow mesoporous Prussian blue nanoparticles coated with chitosan), applied topically every two days up to day 7.	Relative wound size, CFU count in the wound on day 2 of treatment.	Significant reduction in CFU on day 2 of treatment by Chi@HMPB@CBD compared to control group, and by Chi@HMPB@CBD plus laser group compared to Chi@HMPB@CBD group. Significant reduction in relative wound size by Chi@HMPB@CBD plus laser compared to the control group on day 5. Complete wound closure was not observed in any of the groups by day 9. CBD resulted in lower relative wound size compared to the control group on all the days, although not statistically significant. Chi@HMPB@CBD resulted in lower relative wound size compared to Chi@HMPB on all the days, although not statistically significant. Chi@HMPB@CBD plus laser resulted in lower relative wound size compared to Chi@HMPB@CBD on all the days, although not statistically significant. No significant differences in body weight were observed. CBD resulted in a significant increase in WBC count on day 11.	1
Zhong et al. 2022 [139] In vivo, laboratory-reared	Experimental	**Experiment B:** in vivo mouse skin infection model for chronic wounds. **Experimental group** Similar to above, using 6-week-old female MKR mice with induced type 2 diabetes.	Similar to above.	Similar to above.	Relative wound size in Chi@HMPB@CBD plus laser group was significantly less compared to the control group on days 7, 9, and 11. Complete wound closure by Chi@HMPB@CBD plus laser group on day 11, which was significant compared to the control group. CBD resulted in lower relative wound size compared to the control group on all the days, although not statistically significant. ~55.2% and ~98.3% of bacteria were killed in the Chi@HMPB@CBD NPs and Chi@HMPB@CBD NPs plus laser groups on day 2 respectively. Chi@HMPB@CBD resulted in significant reduction in CFU count compared to control on day 2 (*p* < 0.001). Chi@HMPB@CBD NPs plus laser groups resulted in significant reduction in CFU count compared to Chi@HMPB@CBD on day 2 (*p* < 0.001). No significant differences in body weight were observed.	1
McCormick et al. 2023 [140] In vivo, laboratory-reared	Experimental	**Experiment A:** Murine model of cutaneous lupus erythematosus (prophylactic). **Experimental group** 10-week-old female MRL/lpr mice, which spontaneously develop generalized autoimmune disease including skin lesions clinically and histologically similar to cutaneous lupus erythematosus were used. Mice were sacrificed at 20 weeks.	**Treatment** 50 mg of 1% Anandamide loaded into a silica nanoparticle in coconut oil (AEA-NP, *n* = 10) was topically applied to interscapular region twice daily for 10 weeks. **Comparator ** 50 mg of silica nanoparticle in coconut oil (NP, vehicle, *n* = 10) was topically applied to interscapular region twice daily for 10 weeks, and untreated controls (*n* = 5).	Lesion score for developing lesions and severity of existing lesions (erythema, thickness, scaling, and alopecia).	Significantly lower final lesion severity scores after 10 weeks of treatment with AEA-NP (M = 1.87 ± 1.82) compared to vehicle (M = 5.65 ± 2.86, *p* < 0.01) and untreated controls (M = 9.2 ± 4.65, *p* < 0.01).	1
McCormick et al. 2023 [140] In vivo, laboratory-reared	Experimental	**Experiment B:** Murine model of cutaneous lupus erythematosus (interventional). **Experimental group** Female MRL/lpr mice from 8 weeks of age with developed skin lesions with skin plaque score of 1 were enrolled and treated for 10 weeks.	**Treatment** 50 mg of either 1% AEA-NP in coconut oil (*n* = 10) or 1% unencapsulated anandamide in coconut oil (AEA-UE; *n* = 10) twice weekly for 10 weeks applied topically on head and interscapular area. **Comparator ** Untreated controls (*n* = 10).	Weekly lesion score (erythema, thickness, scaling, and alopecia).	AEA-NP resulted in lower average skin lesion scores compared to untreated controls from week 3 of treatment (*p* < 0.05) until the end of the treatment period (week 4–10 *p* < 0.0001). AEA-NP had significantly lower average skin lesion scores than AEA-UE mice from weeks 5–10 of treatment (*p* < 0.05). AEA-UE resulted in lower skin scores compared to untreated controls from weeks 2–10.	1
Cham et al. 2024 [136] In vivo, laboratory-reared	Experimental	In vivo mice dermal infection model **Experimental group** The skin of the area for the infection was depilated and a blood oozing patch was created on 3–4-week-old male Balb/c mice (22–26 g). An overnight-grown culture of MRSA-15187 equivalent to 0.5 McFarland standard in normal saline was applied for 24 h on the abraded skin.	**Treatment** 0.5% Tetrahydrocannabidiol (THCBD) formulated in a PEG-400 ointment base (*n* = 15), or 2% THCBD formulated in a PEG-400 ointment base (*n* = 15) was applied twice daily for 5 days. **Comparator ** 2% mupirocin (positive control group, *n* = 15), or control (drug-free control, *n* = 18) twice daily for 5 days	Number of CFU **** in a dissected 1 cm^2^ skin patch.	2% THCBD and mupirocin reduced initial bacterial load by ∼4-log on days 2 and 3, respectively. 0.5% THCBD resulted in a 2-log CFU reduction on day 2. All the treatments resulted in >3-log reduction by day 4 and reduced the bacterial load to minimum by day 5.	1

* SSD: silver sulfadiazine, ** CBD: cannabidiol, *** UMF: unique manuka factor, **** CFU: colony forming units

**Table 2:** Formulation details of medicinal cannabis-based products used within in vivo studies.

Reference	Formulation and/or Route of Administration	Content
Mehrabani et al. 2016 [131]	Topical formulation	Oils of sesame (*Sesamum indicum* L.) seed (60%), wild pistachio (*Pistacia atlantica* Desf.) fruit (20%), hemp (*Cannabis sativa* L.) fruit (12%), and walnut (*Juglans regia* L.) seed (8%). Dose: 100 mg/mouse.
Wang et al. 2016 [67]	Intraperitoneal injection	Gp1a dissolved in 5% dimethyl sulfoxide/2% Tween-80/physiological saline. Dose: 3 mg/kg body weight.
Klein et al. 2018 [132]	Intraperitoneal injection	Synthetic CBD * (≥99 purity) dissolved in 2% polyoxyethylene sorbitan monooleate (Tween 80) in saline. Immediately prepared before administration in a volume of 1 mL/kg. Protected from light. Dose: 5 or 10 mg/kg body weight.
Koyama et al. 2019 [134]	A silicone ring was attached to the lesion by a liquid sealing bandage (serving as a reservoir for the treatment) Topical	1 μM JWH133 in olive oil daily for 5 days. Dose: 50 μL/day.
McIver et al. 2020 [133]	Topical	Daily application of 1% CBD * extract (= 10 mg CBD) in UMF ** 5 manuka honey for 42 days with initial 13 days of bandaging. Dose: 2 mL/day.
Blaskovich et al. 2021 [135]	Topical	5% synthetic CBD * either in BTX 1503, 1503 gel, or BTX 1204 gel. Dose: 50 μL.
Zhao et al. 2021 [137]	Topical	4 mg/mL Gp1a in scramble Tm hydrogel. Dose: 50 μL.
Zheng et al. 2022 [138]	Topical	2% *w/v* CBD * containing alginate-Zn hydrogel. Dose: not given.
Zhong et al. 2022 [139]	Topical	Natural CBD * (10 μg/mL), Chi@HMPB NPs (hollow mesoporous Prussian blue nanoparticles coated with chitosan), Chi@HMPB@CBD NPs (CBD-loaded hollow mesoporous Prussian blue nanoparticles coated with chitosan; containing 100 μg/mL HMPB NPs). Prior to fixing the wounds, Chi@HMPB@CBD plus laser groups were irradiated with an 808 nm laser (1 W/cm^2^, 3 min). Dose: 100 μL.
McCormick et al. 2023 [140]	Topical	1% Anandamide loaded into a silica nanoparticle in coconut oil (AEA-NP), 1% unencapsulated AEA in coconut oil twice weekly (AEA-UE). Dose: 50 mg.
Cham et al. 2024 [136]	Topical	Tetrahydrocannabidiol (>99% purity, synthetic) formulated in a PEG-400 ointment base (0.5% and 2%). Dose: not given.

* CBD: cannabidiol, ** UMF: unique manuka factor

**Table 3:** Descriptive characteristics of included human studies (*n* = 7).

Study Setting and Location	Study Design	Study Population	Wound Aetiology/Classification	Intervention Descriptions		Outcome Measures	Follow-up	Study Outcomes	Quality Score
				Test	Comparator				
Maida and Corban 2017 [144] Ambulatory setting, Canada	Prospective case series	Patients suffering from Pyoderma Gangrenosum (*n* = 3), mean age = 62 years, gender = 2 females and 1 male.	Pyoderma gangrenosum in lower extremities.	0.5–1 mL of topical medical cannabis oil (5–7 mg/mL THC * and 6–9 mg/mL CBD **) applied to the wound bed 1–2 times a day. Then, wounds were bandaged. Treatment was tailored to the patient’s needs.	Condition before the treatment.	Reduction in average daily pain score, reduction in average daily morphine (MSE ***) usage.	Not reported.	Statistically significant reduction (*p* < 0.05) in the average daily pain score was observed in two patients. A clinically significant reduction (>60%) in average daily pain score was observed in all the patients. A substantial reduction in average daily MSE *** usage was observed (*p* < 0.05).	5
Chelliah et al. 2018 [141] Community setting, US	Case report, observational	Paediatric epidermolysis bullosa patients (*n* = 3), mean age = 4.5 years, gender = 2 females and 1 male.	Chronic non-healing epidermolysis bullosa wounds.	CBD ** oil (alone or mixed with emu oil) applied topically on the blisters 2–3 times daily. Self-initiated treatment.	Condition before the treatment.	Healing time, reduction in blistering, reduction in pain.	Not reported	Decreased blistering, decreased healing time, reduction in pain, stopped other analgesics, and improved ambulation (*p* values are not given). No significant side effects were reported.	6
Schrader et al. 2019 [143] Ambulatory setting, The Netherlands	Open-label trial	Patients suffering from epidermolysis bullosa (*n* = 3), mean age = 47, gender = 2 males and a female.	Chronic epidermolysis bullosa.	Whole *C. sativa* plant extract, containing 20 mg/mL CBD **, 13 mg/mL THC *, dissolved in refined peanut oil (arachis oil) [149]. Treatment was tailored to the patient needs.	Condition before the treatment.	Reduction in pain, reduction in pruritus, reduction in the overall intake of analgesic medications.	8 months–2 years	Reduced pain scores (from 9 out of 10 to 1–4 out of 10), Reduction in pruritus, discontinuation or reduction in other analgesics and amitriptyline (*p* values are not given). Side effects: increased appetite.	6
Maida et al. 2020 [142] Ambulatory setting, Canada	Prospective open-label cohort study.	Patients with non-uremic calciphylaxis leg ulcers (number of patients = 2, number of wounds = 3), The mean age is 77 years, 2 females.	Painful, non-healing, non-uremic calciphylaxis leg ulcers of more than six months of duration.	Wounds were gently cleansed with sterile normal saline daily. Formulations VS-12 **** and VS-14 ***** were applied daily to the wound beds and 4–6 cm radial cuff of peri-wound area, respectively, until complete wound closure. Both formulations contained CBD ** (3.75 mg/mL), THC * (<1 mg/mL), quercetin (31.25 mg/mL), disomin (25.31 mg/mL), hesperidin (2.5 mg/mL), and β-caryophyllene (152.69 mg/mL) in different bases. Wounds were covered with a layer of Jelonet and Mesorb (dressings), then by spiral bandaging.	Opioid for analgesic use before the treatment.	Wound healing/full closure, time for complete wound closure, reduction in pain, reduction in analgesic opioid usage.	Until complete wound closure.	50% wound closure after 32–41 days. Complete wound closure after a mean of 76.3 days. Re-epithelialisation rate was 1.5–1.8%/day. 33% reduction in analgesic usage by 18–19 days (clinically significant). Analgesics were no longer required after a mean of 63 days. Improved ambulation (*p* values are not given). No significant side effects were reported.	7
Maida et al. 2021 [145] Ambulatory setting, Canada	Open-label trial	Patients suffering from venous leg ulcers, (number of patients = 14; number of wounds = 16); mean age = 75.8 years; gender = 8 females and 6 males.	Chronic and non-healing leg ulcers. These did not respond to a minimum of 4 weeks of compression therapy with all available best practices (local treatments).	Wounds were gently cleansed with sterile normal saline every two days. Formulations VS-12 **** and VS-14 ***** were applied to the wound beds and 4–6 cm radial cuff of peri-wound area, respectively, every two days. Then, the wounds were bandaged with a layer of Jelonet^®^ and Mesorb^®^. Then, an inelastic compression bandage was applied.	None.	Complete wound closure, healing time.	Until complete wound closure.	The median time for complete wound healing was 34 days. The closing time for wounds 0–10 cm (*n* = 6 wounds) in size was reported to be 38 days, while the treatment for wounds >10 cm (*n* = 10 wounds) was estimated to be 29 days. 81% of wounds were closed completely (others were lost to follow-up). The median rate of surface area change was −3.3 cm^2^/30 days (*p* values are not given). No patient developed hypertrophic scars or keloids. No significant side effects were reported.	6
Diaz et al. 2021 [146] Ambulatory setting, Canada	Case report, observational	A 37-year-old female patient with a pressure ulcer.	Progressively worsening pressure ulcer between right iliac crest and right rib cage for 5 years, unresponsive to multiple interventions including wound dressings, antibiotics, and topical treatments.	Three oral medicinal cannabis oils containing THC and CBD at different times of the day. THC and CBD daily doses were gradually increased and stabilized over time from 0.11 mg/kg THC and 0.48 mg/kg CBD in week 1 to 0.67 mg/kg THC and 1.28 mg/kg CBD in week 8. A foam-padded dressing (Allevyn; Smith + Nephew) was also used to relieve pressure on the wound.	Condition before the treatment.	Complete wound closure, healing time.	2 months.	Reduction in wound size, pain, and erythema was reported within 2 weeks of treatment initiation. Complete wound healing was achieved in 2 months. Improved sleep quality and decreased anxiety were reported. The presence or absence of side effects was not reported.	6
Umpreecha et al. 2023 [147] Ambulatory setting, Thailand	Randomized, parallel double-blind, controlled trial.	Patients with recurrent aphthous ulcers at least 2 times/year on nonkeratinized oral mucosa, aged 18–65 years (*n* = 69).	1–3 minor (2–10 mm in diameter) aphthous ulcers of ≤48 h duration with easy access for evaluation.	0.1% CBD ** oral paste applied to ulcers with a provided calibrated spoon 3 times per day after meals for 7 days.	Control groups received 0.1% triamcinolone acetonide (TA), or placebo applied similarly.	Ulcer size, daily pain ratings.	7 days.	Pseudomembranous ulcer size and erythematous border size were increased with placebo until day 5 and then began to reduce. Ulcer size and erythematous border size were reduced with both CBD and TA compared to initial size over time. Statistically significant ulcer size and erythematous border size reductions were observed with CBD and TA compared to placebo on days 2, 5, and 7. CBD significantly reduced the pain levels on day 5 compared to placebo (*p* < 0.05). TA significantly reduced the pain levels on days 4, 5, and 7 compared to placebo (*p* < 0.05). Compared to CBD, TA better reduced the ulcer size and erythematous border sizes. No side effects (local or systemic) were reported.	10

* THC: tetrahydrocannabinol, ** CBD: cannabidiol, *** MSE: morphine sulfate equivalent, VS-12 **** and VS-14 *****: names of the formulation.

**Table 4:** Formulation details of medicinal cannabis-based products in human studies.

Reference	Formulation and Route of Administration	Content
Maida and Corban 2017 [144]	Topical oil	THC * (5 or 7 mg/mL) and CBD ** (6 or 9 mg/mL) in sunflower oil [(ARGYLE^TM^ THC 5 mg/mL and CBD 6 mg/mL) from TWEED Inc.; Bedrolite^TM^ (THC 7 mg/mL and CBD 9 mg/mL) from Bedrocan Inc.]
Chelliah et al. 2018 [141]	Different topical formulations such as spray (oil), oil, and cream	CBD ** oil with or without emu oil
Schrader et al. 2019 [143]	Sublingual oil	Whole *Cannabis sativa* plant extract, containing 20 mg/mL CBD **, 13 mg/mL THC *, dissolved in refined peanut oil (arachis oil) [149]
Maida et al. 2020 [142]	Topical formulations	Formulation VS-12 *** (base: 1:1 *v/v* hyaluronic acid and *Aloe vera* gel) applied to the wound beds. Formulation VS-14 **** (liposomal base) were applied to 4–6 cm radial cuff of peri-wound integument. Both formulations contain CBD ** (3.75 mg/mL), THC * (<1 mg/mL), quercetin (31.25 mg/mL), diosmin (25.31 mg/mL), hersperidin (2.5 mg/mL), and β-caryophyllene (152.69 mg/mL)
Maida et al. 2021 [145]	Topical formulations	Same as above
Diaz et al. 2021 [146]	Oral oil	CBD ** dominant oil: Yellow Cannabis Oil, 1:20 THC:CBD (Spectrum Therapeutics) for daytime use. THC * dominant oils: Red No. 1 Oil, 26.3:0 THC:CBD (Spectrum Therapeutics), and Red No. 2 Oil, 26.3:0 THC:CBD (Spectrum Therapeutics) for use in the daytime and just before sleep
Umpreecha et al. 2023 [147]	Oral paste	0.1% CBD (natural) oral paste

* THC: tetrahydrocannabinol, ** CBD: cannabidiol, VS-12 *** and VS-14 ****: names of the formulation.

## Data Availability

Data are contained within the article or Supplementary Materials.

## Supplementary Materials

The following supporting information can be downloaded at: https://www.mdpi.com/article/10.3390/pharmaceutics16081081/s1, Table S1: PRISMA 2020 checklist; Table S2: PRISMA 2020 for abstracts checklist; Supplementary material S1: Search strategies; Table S3: Summary of ex vivo studies on wound healing potential; Table S4: Summary of excluded preclinical studies (Descriptive characteristics); Table S5: Summary of excluded preclinical studies (Formulation details); Table S6: Summary of excluded clinical studies (Descriptive characteristics); Table S7: Summary of excluded clinical studies (Formulation details); Supplementary material S2: Details of risk-of-bias assessment tools; Supplementary material S3: Details of rejected articles; Table S8: Outcome measures of selected studies; Supplementary material S4: Amendments to PROSPERO registration from the initial submission.
