# Supplementary material for: Cannabinoids in Integumentary Wound Care: A Systematic Review of Emerging Preclinical and Clinical Evidence
Source: Pharmaceutics. 2024 Aug 17;16(8):1081. doi: 10.3390/pharmaceutics16081081 (PMC11359183; doi:10.3390/pharmaceutics16081081)
Supplement: Supplementary file 1 [file pharmaceutics-16-01081-s001.zip › pharmaceutics-3032184-supplementary.pdf]

Table S1: PRISMA 2020 Checklist

Section and Topic	Item #	Checklist item	Location where item is reported
**TITLE**			
Title	1	Identify the report as a systematic review.	title
**ABSTRACT**			
Abstract	2	See the PRISMA 2020 for Abstracts checklist.	abstract
**INTRODUCTION**			
Rationale	3	Describe the rationale for the review in the context of existing knowledge.	Introduction
Objectives	4	Provide an explicit statement of the objective(s) or question(s) the review addresses.	Introduction
**METHODS**			
Eligibility criteria	5	Specify the inclusion and exclusion criteria for the review and how studies were grouped for the syntheses.	Methods
Information sources	6	Specify all databases, registers, websites, organisations, reference lists and other sources searched or consulted to identify studies. Specify the date when each source was last searched or consulted.	Methods, Supplementary material S1
Search strategy	7	Present the full search strategies for all databases, registers and websites, including any filters and limits used.	Supplementary material S1
Selection process	8	Specify the methods used to decide whether a study met the inclusion criteria of the review, including how many reviewers screened each record and each report retrieved, whether they worked independently, and if applicable, details of automation tools used in the process.	Methods
Data collection process	9	Specify the methods used to collect data from reports, including how many reviewers collected data from each report, whether they worked independently, any processes for obtaining or confirming data from study investigators, and if applicable, details of automation tools used in the process.	Method
Data items	10a	List and define all outcomes for which data were sought. Specify whether all results that were compatible with each outcome domain in each study were sought (e.g. for all measures, time points, analyses), and if not, the methods used to decide which results to collect.	Methods, Results, Table S8
	10b	List and define all other variables for which data were sought (e.g. participant and intervention characteristics, funding sources). Describe any assumptions made about any missing or unclear information.	Methods-data extraction, Table S8
Study risk of bias assessment	11	Specify the methods used to assess risk of bias in the included studies, including details of the tool(s) used, how many reviewers assessed each study and whether they worked independently, and if applicable, details of automation tools used in the process.	Method, Supplementary material S2
Effect measures	12	Specify for each outcome the effect measure(s) (e.g. risk ratio, mean difference) used in the synthesis or presentation of results.	Not applicable
Synthesis methods	13a	Describe the processes used to decide which studies were eligible for each synthesis (e.g. tabulating the study intervention characteristics and comparing against the planned groups for each synthesis (item #5)).	Not applicable
	13b	Describe any methods required to prepare the data for presentation or synthesis, such as handling of missing summary statistics, or data conversions.	Not applicable
	13c	Describe any methods used to tabulate or visually display results of individual studies and syntheses.	Not applicable

Table S1: PRISMA 2020 Checklist

Section and Topic	Item #	Checklist item	Location where item is reported
	13d	Describe any methods used to synthesize results and provide a rationale for the choice(s). If meta-analysis was performed, describe the model(s), method(s) to identify the presence and extent of statistical heterogeneity, and software package(s) used.	Not applicable
	13e	Describe any methods used to explore possible causes of heterogeneity among study results (e.g. subgroup analysis, meta-regression).	Not applicable
	13f	Describe any sensitivity analyses conducted to assess robustness of the synthesized results.	Not applicable
Reporting bias assessment	14	Describe any methods used to assess risk of bias due to missing results in a synthesis (arising from reporting biases).	Not applicable
Certainty assessment	15	Describe any methods used to assess certainty (or confidence) in the body of evidence for an outcome.	Not applicable
**RESULTS**			
Study selection	16a	Describe the results of the search and selection process, from the number of records identified in the search to the number of studies included in the review, ideally using a flow diagram.	PRISMA chart, results
	16b	Cite studies that might appear to meet the inclusion criteria, but which were excluded, and explain why they were excluded.	Supplementary material S3
Study characteristics	17	Cite each included study and present its characteristics.	Results<br>Tables 1-4
Risk of bias in studies	18	Present assessments of risk of bias for each included study.	Results, Table 1, and 3
Results of individual studies	19	For all outcomes, present, for each study: (a) summary statistics for each group (where appropriate) and (b) an effect estimate and its precision (e.g. confidence/credible interval), ideally using structured tables or plots.	Not applicable
Results of syntheses	20a	For each synthesis, briefly summarise the characteristics and risk of bias among contributing studies.	Not applicable
	20b	Present results of all statistical syntheses conducted. If meta-analysis was done, present for each the summary estimate and its precision (e.g. confidence/credible interval) and measures of statistical heterogeneity. If comparing groups, describe the direction of the effect.	Not applicable
	20c	Present results of all investigations of possible causes of heterogeneity among study results.	Not applicable
	20d	Present results of all sensitivity analyses conducted to assess the robustness of the synthesized results.	Not applicable
Reporting biases	21	Present assessments of risk of bias due to missing results (arising from reporting biases) for each synthesis assessed.	Not applicable
Certainty of evidence	22	Present assessments of certainty (or confidence) in the body of evidence for each outcome assessed.	Not applicable
**DISCUSSION**			

Table S1: PRISMA 2020 Checklist

Section and Topic	Item #	Checklist item	Location where item is reported
Discussion	23a	Provide a general interpretation of the results in the context of other evidence.	Discussion
	23b	Discuss any limitations of the evidence included in the review.	Discussion
	23c	Discuss any limitations of the review processes used.	Discussion
	23d	Discuss implications of the results for practice, policy, and future research.	Discussion
**OTHER INFORMATION**			
Registration and protocol	24a	Provide registration information for the review, including register name and registration number, or state that the review was not registered.	Method
	24b	Indicate where the review protocol can be accessed, or state that a protocol was not prepared.	Method
	24c	Describe and explain any amendments to information provided at registration or in the protocol.	Supplementary material S4
Support	25	Describe sources of financial or non-financial support for the review, and the role of the funders or sponsors in the review.	Funding
Competing interests	26	Declare any competing interests of review authors.	Competing interests
Availability of data, code and other materials	27	Report which of the following are publicly available and where they can be found: template data collection forms; data extracted from included studies; data used for all analyses; analytic code; any other materials used in the review.	Results

From: Page MJ, McKenzie JE, Bossuyt PM, Boutron I, Hoffmann TC, Mulrow CD, et al. The PRISMA 2020 statement: an updated guideline for reporting systematic reviews. BMJ 2021;372:n71. doi: 10.1136/bmj.n71

For more information, visit: http://www.prisma-statement.org/

Table S2: PRISMA 2020 for Abstracts Checklist

Section and Topic	Item #	Checklist item	Reported (Yes/No)
**TITLE**			
Title	1	Identify the report as a systematic review.	Yes
**BACKGROUND**			
Objectives	2	Provide an explicit statement of the main objective(s) or question(s) the review addresses.	Yes
**METHODS**			
Eligibility criteria	3	Specify the inclusion and exclusion criteria for the review.	Yes
Information sources	4	Specify the information sources (e.g. databases, registers) used to identify studies and the date when each was last searched.	Yes
Risk of bias	5	Specify the methods used to assess risk of bias in the included studies.	Yes
Synthesis of results	6	Specify the methods used to present and synthesise results.	Yes
**RESULTS**			
Included studies	7	Give the total number of included studies and participants and summarise relevant characteristics of studies.	Yes
Synthesis of results	8	Present results for main outcomes, preferably indicating the number of included studies and participants for each. If meta-analysis was done, report the summary estimate and confidence/credible interval. If comparing groups, indicate the direction of the effect (i.e. which group is favoured).	Yes
**DISCUSSION**			
Limitations of evidence	9	Provide a brief summary of the limitations of the evidence included in the review (e.g. study risk of bias, inconsistency and imprecision).	Yes
Interpretation	10	Provide a general interpretation of the results and important implications.	Yes
**OTHER**			
Funding	11	Specify the primary source of funding for the review.	Yes
Registration	12	Provide the register name and registration number.	Yes

Supplementary material S1: Search strategies

CINAHL Plus with Full Text (EBSCO)

Search date: 10th July 2021

No	Search	No of articles
#1	(TI (cannabis OR hemp OR marijuana OR phytocannabinoid* OR cannabinoid* OR cannabichromene* OR cannabicyclol* OR cannabidiol* OR cannabielsoin* OR cannabigerol* OR cannabinodiol* OR cannabinol* OR cannabitriol* OR tetrahydrocannabinol* OR (MM "Medical Marijuana") OR (MM "Cannabinoids+")) OR AB (cannabis OR hemp OR marijuana OR phytocannabinoid* OR cannabinoid* OR cannabichromene* OR cannabicyclol* OR cannabidiol* OR cannabielsoin* OR cannabigerol* OR cannabinodiol* OR cannabinol* OR cannabitriol* OR tetrahydrocannabinol* OR (MM "Medical Marijuana") OR (MM "Cannabinoids+"))) AND ((TI ("wound*" OR "wound heal*" OR (MM "Wound Healing+") OR (MM "Wound Infection+")) OR AB ("wound*" OR "wound heal*" OR (MM "Wound Healing+") OR (MM "Wound Infection+"))) OR (TI (antibacterial* OR "anti bacterial*" OR "anti-bacterial" OR bactericid* OR bacteriostatic* OR antiseptic OR antibiotic OR (MM "Anti-Bacterial Agents+")) OR AB (antibacterial* OR "anti bacterial*" OR "anti-bacterial" OR bactericid* OR bacteriostatic* OR antiseptic* OR antibiotic* OR (MM "Anti-Bacterial Agents+"))) AND (TI ("skin and soft tissue infection*" OR ssti* OR "skin infection*" OR "acute bacterial skin and skin structure infection*" OR absssi* OR "dermatological practice*" OR dermatolog* OR "skin care" OR "skin condition*" OR "skin disease*" OR "skin health" OR "skin problem*" OR "skin treat*" OR "skin*" OR "skin approach*" OR topical* OR (MM "Dermatologic Agents+") OR (MM "Soft Tissue Infections") OR (MM "Skin Diseases, Bacterial+")) OR AB ("skin and soft tissue infection*" OR ssti* OR "skin infection*" OR "acute bacterial skin and skin structure infection*" OR absssi* OR "dermatological practice*" OR dermatolog* OR "skin care" OR "skin condition*" OR "skin disease*" OR "skin health" OR "skin problem*" OR "skin treat*" OR "skin*" OR "skin approach*" OR topical* OR (MM "Dermatologic Agents+") OR (MM "Soft Tissue Infections") OR (MM "Skin Diseases, Bacterial+"))))<br>Expanders - Apply related words; Apply equivalent subjects<br>Search modes - Find any of my search terms	27

Tl: title, AB: abstract, MM: exact major subject heading, MH: Exact subject heading, +: explode

Medline via EBSCOhost

Search date: 10th July 2021

No	Search	No of articles
#1	(TI (cannabis OR hemp OR marijuana OR phytocannabinoid* OR cannabinoid* OR cannabichromene* OR cannabicyclol* OR cannabidiol* OR cannabielsoin* OR cannabigerol* OR cannabinodiol* OR cannabinol* OR cannabitriol* OR tetrahydrocannabinol* OR (MM "Medical Marijuana") OR (MM "Cannabinoids+")) OR AB (cannabis OR hemp OR marijuana OR phytocannabinoid* OR cannabinoid* OR cannabichromene* OR cannabicyclol* OR cannabidiol* OR cannabielsoin* OR cannabigerol* OR cannabinodiol* OR cannabinol* OR cannabitriol* OR tetrahydrocannabinol* OR (MM "Medical Marijuana") OR (MM "Cannabinoids+"))) AND ((TI ("wound*" OR "wound heal*" OR (MM "Wound Healing+") OR (MM "Wound Infection+")) OR AB ("wound*" OR "wound heal*" OR (MM "Wound Healing+") OR (MM "Wound Infection+"))) OR (TI (antibacterial* OR "anti bacterial*" OR "anti-bacterial" OR bactericid* OR bacteriostatic* OR antiseptic OR antibiotic OR (MM "Anti-Bacterial Agents+")) OR AB (antibacterial* OR "anti bacterial*" OR "anti-bacterial" OR bactericid* OR bacteriostatic* OR antiseptic* OR antibiotic* OR (MM "Anti-Bacterial Agents+"))) AND (TI ("skin and soft tissue infection*" OR ssti* OR "skin infection*" OR "acute bacterial skin and skin structure infection*" OR absssi* OR "dermatological practice*" OR dermatolog* OR "skin care" OR "skin condition*" OR "skin disease*" OR "skin health" OR "skin problem*" OR "skin treat*" OR "skin*" OR "skin approach*" OR topical* OR (MM "Dermatologic Agents+") OR (MM "Soft Tissue Infections") OR (MM "Skin Diseases, Bacterial+")) OR AB ("skin and soft tissue infection*" OR ssti* OR	140

	"skin infection*" OR "acute bacterial skin and skin structure infection*" OR absssi* OR "dermatological practice*" OR dermatolog* OR "skin care" OR "skin condition*" OR "skin disease*" OR "skin health" OR "skin problem*" OR "skin treat*" OR "skin*" OR "skin approach*" OR topical* OR (MM "Dermatologic Agents+") OR (MM "Soft Tissue Infections") OR (MM "Skin Diseases, Bacterial+"))))<br>Expanders - Apply related words; Apply equivalent subjects<br>Search modes - Find any of my search terms	

Tl: title, AB: abstract, MM: exact major subject heading, MH: Exact subject heading, +: explode

Embase via Scopus

Search date: 10th July 2021

No	Search	No of articles
#1	TITLE-ABS-KEY (cannabis OR hemp OR marijuana OR phytocannabinoid* OR cannabinoid* OR cannabichromene* OR cannabicyclol* OR cannabidiol* OR cannabielsoin* OR cannabigerol* OR cannabinodiol* OR cannabinol* OR cannabitriol* OR tetrahydrocannabinol*)	95,886
#2	TITLE-ABS-KEY ("wound*" OR "wound heal*")	555,626
#3	TITLE-ABS-KEY (antibacterial* OR "anti bacterial*" OR "anti-bacterial" OR bactericid* OR bacteriostatic* OR antiseptic* OR antibiotic*)	1,119,164
#4	TITLE-ABS-KEY ("skin and soft tissue infection*" OR ssti* OR "skin infection*" OR "acute bacterial skin and skin structure infection*" OR absssi* OR "dermatological practice*" OR dermatolog* OR "skin care" OR "skin condition*" OR "skin disease*" OR "skin health" OR "skin problem*" OR "skin treat*" OR "skin*" OR "skin approach*" OR topical*)	1,561,512
#5	#1 AND #2	550
#6	#1 AND #3 AND #4	75
#7	#5 0R #6<br>((TITLE-ABS-KEY (cannabis OR hemp OR marijuana OR phytocannabinoid* OR cannabinoid* OR cannabichromene* OR cannabicyclol* OR cannabidiol* OR cannabielsoin* OR cannabigerol* OR cannabinodiol* OR cannabinol* OR cannabitriol* OR tetrahydrocannabinol*)) AND (TITLE-ABS-KEY ("wound*" OR "wound heal*"))) OR ((TITLE-ABS-KEY (cannabis OR hemp OR marijuana OR phytocannabinoid* OR cannabinoid* OR cannabichromene* OR cannabicyclol* OR cannabidiol* OR cannabielsoin* OR cannabigerol* OR cannabinodiol* OR cannabinol* OR cannabitriol* OR tetrahydrocannabinol*)) AND (TITLE-ABS-KEY (antibacterial* OR "anti bacterial*" OR "anti-bacterial" OR bactericid* OR bacteriostatic* OR antiseptic* OR antibiotic*)) AND (TITLE-ABS-KEY ("skin and soft tissue infection*" OR ssti* OR "skin infection*" OR "acute bacterial skin and skin structure infection*" OR absssi* OR "dermatological practice*" OR dermatolog* OR "skin care" OR "skin condition*" OR "skin disease*" OR "skin health" OR "skin problem*" OR "skin treat*" OR "skin*" OR "skin approach*" OR topical*)))	616

TITLE-ABS-KEY : article title, abstract, keywords

PubMed

Search date: 10th July 2021

No	Search	No of articles
#1	"cannabis"[Title/Abstract] OR "hemp"[Title/Abstract] OR "marijuana"[Title/Abstract] OR "phytocannabinoid*"[Title/Abstract] OR "cannabinoid*"[Title/Abstract] OR "cannabichromene*"[Title/Abstract] OR "cannabicyclol*"[Title/Abstract] OR "cannabidiol*"[Title/Abstract] OR "cannabielsoin*"[Title/Abstract] OR "cannabigerol*"[Title/Abstract] OR "cannabinodiol*"[Title/Abstract] OR	53,423

	"cannabinol*"[Title/Abstract] OR "cannabitriol*"[Title/Abstract] OR "tetrahydrocannabinol*"[Title/Abstract] OR "cannabinoids"[MeSH Terms] OR "medical marijuana"[MeSH Terms]	
#2	"wound*"[Title/Abstract] OR "wound heal*"[Title/Abstract] OR "Wound Healing"[MeSH Terms] OR "Wound Infection"[MeSH Terms]	342,812
#3	"antibacterial*"[Title/Abstract] OR "anti bacterial*"[Title/Abstract] OR "anti-bacterial"[Title/Abstract] OR "bactericid*"[Title/Abstract] OR "bacteriostatic*"[Title/Abstract] OR "antiseptic*"[Title/Abstract] OR "antibiotic*"[Title/Abstract] OR "Anti-Bacterial Agents"[MeSH Terms]	664,515
#4	"skin and soft tissue infection*"[Title/Abstract] OR "ssti*"[All Fields] OR "skin infection*"[Title/Abstract] OR "acute bacterial skin and skin structure infection*"[Title/Abstract] OR "absssi*"[All Fields] OR "dermatological practice*"[Title/Abstract] OR "dermatolog*"[All Fields] OR "skin care"[Title/Abstract] OR "skin condition*"[Title/Abstract] OR "skin disease*"[Title/Abstract] OR "skin health"[Title/Abstract] OR "skin problem*"[Title/Abstract] OR "skin treat*"[Title/Abstract] OR "skin*"[Title/Abstract] OR "skin approach*"[Title/Abstract] OR "topical*"[All Fields] OR "Dermatologic Agents"[MeSH Terms] OR "Soft Tissue Infections"[MeSH Terms]	1,116,538
#5	#3 AND #4	58,193
#6	#2 OR #5	392,921
#7	#1 AND #6	167

Web of Science (WoS)

Search date: 11th July 2021

No	Search	No of articles
#1	TS=(cannabis OR hemp OR marijuana OR phytocannabinoid* OR cannabinoid* OR cannabichromene* OR cannabicyclol* OR cannabidiol* OR cannabielsoin* OR cannabigerol* OR cannabinodiol* OR cannabinol* OR cannabitriol* OR tetrahydrocannabinol*)	92,605
#2	TS=("wound*" OR "wound heal*")	527,300
#3	TS=(antibacterial* OR “anti bacterial*” OR “anti-bacterial” OR bactericid* OR bacteriostatic* OR antiseptic* OR antibiotic*)	1,150,112
#4	TS=("skin and soft tissue infection*" OR ssti* OR "skin infection*" OR "acute bacterial skin and skin structure infection*" OR absssi* OR "dermatological practice*" OR dermatolog* OR "skin care" OR "skin condition*" OR "skin disease*" OR "skin health" OR "skin problem*" OR "skin treat*" OR "skin*" OR "skin approach*" OR topical*)	1,600,595
#5	#3 AND #4	91,551
#6	#2 OR #5	605,184
#7	#1 AND #6	580

TS: topic (title, abstract and key words)

Cochrane library

Search date: 20th July 2021

No	Search	No of articles
#1	(cannabis OR hemp OR marijuana OR phytocannabinoid* OR cannabinoid* OR cannabichromene* OR cannabicyclol* OR cannabidiol* OR cannabielsoin* OR cannabigerol* OR cannabinodiol* OR cannabinol* OR cannabitriol* OR tetrahydrocannabinol*):ti,ab,kw	4,628
#2	MeSH descriptor: [Cannabinoids] explode all trees	864
#3	MeSH descriptor: [Medical Marijuana] explode all trees	17
#4	#1 OR #2 OR #3	4,710

Supplementary material S1: Search strategies

#5	(wound* OR wound NEXT heal*):ti,ab,kw	31,634
#6	MeSH descriptor: [Wound Healing] explode all trees	6,028
#7	MeSH descriptor: [Wound Infection] explode all trees	3,677
#8	#5 OR #6 OR #7	32,627
#9	(antibacterial* OR anti NEXT bacterial* OR anti-bacterial OR bactericid* OR bacteriostatic* OR antiseptic* OR antibiotic*):ti,ab,kw	41,371
#10	MeSH descriptor: [Anti-Bacterial Agents] explode all trees	12,430
#11	#9 OR #10	42,291
#12	(skin and soft NEXT tissue NEXT infection* OR SSTI* OR skin NEXT infection* OR acute NEXT bacterial NEXT skin and skin NEXT structure NEXT infection* OR ABSSSI* OR dermatological NEXT practice* OR dermatolog* OR skin NEXT care OR skin NEXT condition* OR skin NEXT disease* OR skin NEXT health OR skin NEXT problem* OR skin NEXT treat* OR skin* OR skin NEXT approach* OR topical*):ti,ab,kw	89,139
#13	MeSH descriptor: [Dermatologic Agents] explode all trees	3,410
#14	MeSH descriptor: [Soft Tissue Infections] explode all trees	100
#15	#12 OR #13 OR #14	
#16	#11 AND #15	5,721
#17	#8 OR #16	36,923
#18	#1 AND #17	14 (trials)

ti,ab,kw: title, abstract and key words

(LILACS) Latin America and Caribbean health Sciences Literature

https://lilacs.bvsalud.org/en/

Search date: 17th Aug 2021

No	Search	No of articles
#1	cannabis OR hemp OR marijuana OR phytocannabinoid OR cannabinoid OR cannabichromene OR cannabicyclol OR cannabidiol OR cannabielsoin OR cannabigerol OR cannabinodiol OR cannabinol OR cannabitriol OR tetrahydrocannabinol [Words]	1,174
#2	wound OR wound heal [Title words] or wound OR wound heal [Abstract words]	2,904
#3	antibacterial OR antibacterial OR antibacterial OR bactericid OR bacteriostatic OR antiseptic OR antibiotic [Title words] or antibacterial OR antibacterial OR antibacterial OR bactericid OR bacteriostatic OR antiseptic OR antibiotic [Abstract words]	5,662
#4	skin and soft tissue infection OR ssti OR skin infection OR acute bacterial skin and skin structure infection OR absssi OR dermatological practice OR dermatolog OR skin care OR skin condition OR skin disease OR skin health OR skin problem OR skin treat OR skin OR skin approach OR topical [Title words] or skin and soft tissue infection OR ssti OR skin infection OR acute bacterial skin and skin structure infection OR absssi OR dermatological practice OR dermatolog OR skin care OR skin condition OR skin disease OR skin health OR skin problem OR skin treat OR skin OR skin approach OR topical [Abstract words]	13,602
#5	#3 AND #4<br><br>antibacterial OR antibacterial OR antibacterial OR bactericid OR bacteriostatic OR antiseptic OR antibiotic [Title words] or antibacterial OR antibacterial OR antibacterial OR bactericid OR bacteriostatic OR antiseptic OR antibiotic [Abstract words] [Words] and skin and soft tissue infection OR ssti OR skin infection OR acute bacterial skin and skin structure infection OR absssi OR dermatological practice OR dermatolog OR skin care OR skin condition OR skin disease OR skin health OR skin problem OR skin treat OR skin OR skin approach OR topical [Title words] or skin and soft tissue infection OR ssti OR skin infection OR acute bacterial skin and skin structure infection OR absssi OR dermatological practice OR dermatolog OR skin care OR skin condition OR skin disease OR skin health OR skin problem OR skin treat OR skin OR skin approach OR topical [Abstract words] [Words]	406
#6	#2 OR #5	36,236

	wound OR wound heal [Title words] or wound OR wound heal [Abstract words] [Words] or antibacterial OR antibacterial OR antibacterial OR bactericid OR bacteriostatic OR antiseptic OR antibiotic [Title words] or antibacterial OR antibacterial OR antibacterial OR bactericid OR bacteriostatic OR antiseptic OR antibiotic [Abstract words] [Words] and skin and soft tissue infection OR ssti OR skin infection OR acute bacterial skin and skin structure infection OR absssi OR dermatological practice OR dermatolog OR skin care OR skin condition OR skin disease OR skin health OR skin problem OR skin treat OR skin OR skin approach OR topical [Title words] or skin and soft tissue infection OR ssti OR skin infection OR acute bacterial skin and skin structure infection OR absssi OR dermatological practice OR dermatolog OR skin care OR skin condition OR skin disease OR skin health OR skin problem OR skin treat OR skin OR skin approach OR topical [Abstract words] [Words] [Words]	
#7	#1 AND #6<br>cannabis OR hemp OR marijuana OR phytocannabinoid OR cannabinoid OR cannabichromene OR cannabicyclol OR cannabidiol OR cannabielsoin OR cannabigerol OR cannabinodiol OR cannabinol OR cannabitriol OR tetrahydrocannabinol [Words] heal heal or words wound wound [Words] [Words] heal or words wound wound] [Words] or antibacterial OR antibacterial OR antibacterial OR bactericid OR bacteriostatic OR antiseptic OR antibiotic [Title words] or antibacterial OR antibacterial OR antibacterial OR bactericid OR bacteriostatic OR antiseptic OR antibiotic [Abstract words] [Words] and skin and soft tissue OR ssti OR skin infection OR acute bacterial skin and skin structure infection OR absssi OR dermatological practice OR dermatolog OR skin care OR skin condition OR skin disease OR skin health OR skin problem OR skin treat OR skin ORskin approach OR topical OR topical [Title words] OR skin and soft tissue OR skin infection OR acute bacterial skin and skin structure OR absssi OR dermatological practice OR dermatolog OR skin care OR skin condition OR skin disease OR skin health OR skin problem OR skin treat OR skin OR skin approach OR topical [Abstract words] [Words] [Words] [Words]	15<br>Hand searching results in 1

Grey literature

AHRQ (The Agency for Healthcare Research and Quality)

(AHRQ, https://www.ahrq.gov/)

Search date: 21st July 2021

No	Search terms	Hits	After manual search
#1	wound cannabis<br>• Searched entire documents<br>• At least one of these words "wound cannabis"<br>• These file types only "application/pdf"	70	0
#2	wound marijuana<br>• Searched entire documents<br>• At least one of these words "wound marijuana"<br>• These file types only "application/pdf"	21	0
#3	wound cannabinoid<br>• Searched entire documents<br>• At least one of these words "wound cannabinoid"<br>• These file types only "application/pdf"	32	0
#4	skin cannabis<br>• Searched entire documents<br>• At least one of these words "skin cannabis"<br>• These file types only "application/pdf"	96	0
#5	skin marijuana	41	0

	• Searched entire documents<br>• At least one of these words "skin marijuana"<br>• These file types only "application/pdf"		
#6	Skin cannabinoid<br>• Searched entire documents<br>• At least one of these words "skin cannabinoid"<br>• These file types only "application/pdf"	44	0
	Total	304	0

BASE (Bielefeld Academic Search Engine)

(BASE, https://www.base-search.net/)

Search dates: 21st, 22nd and 23rd July 2021

No	Search terms	Hits	After manual search
#1	(wound skin) AND (cannabis marijuana)<br>Entire document searched	461	101
#2	cannabinoid* (wound skin)	479	197
	TOTAL (after removing duplicates)		291

EBSCOhost Open Dissertations

https://biblioboard.com/opendissertations/

Search date: 4th Aug 2021

No	Search	No of articles	After manual search
#1	(TI (cannabis OR hemp OR marijuana OR phytocannabinoid* OR cannabinoid* OR cannabichromene* OR cannabicyclol* OR cannabidiol* OR cannabielsoin* OR cannabigerol* OR cannabinodiol* OR cannabinol* OR cannabitriol* OR tetrahydrocannabinol* OR (MM "Medical Marijuana") OR (MM "Cannabinoids+")) OR AB (cannabis OR hemp OR marijuana OR phytocannabinoid* OR cannabinoid* OR cannabichromene* OR cannabicyclol* OR cannabidiol* OR cannabielsoin* OR cannabigerol* OR cannabinodiol* OR cannabinol* OR cannabitriol* OR tetrahydrocannabinol* OR (MM "Medical Marijuana") OR (MM "Cannabinoids+"))) AND ((TI ("wound*" OR "wound heal*" OR (MM "Wound Healing+") OR (MM "Wound Infection+")) OR AB ("wound*" OR "wound heal*" OR (MM "Wound Healing+") OR (MM "Wound Infection+"))) OR (TI (antibacterial* OR “anti bacterial*” OR “anti-bacterial” OR bactericid* OR bacteriostatic* OR antiseptic OR antibiotic OR (MM "Anti-Bacterial Agents+")) OR AB (antibacterial* OR “anti bacterial*” OR “anti-bacterial” OR bactericid* OR bacteriostatic* OR antiseptic* OR antibiotic* OR (MM "Anti-Bacterial Agents+"))) AND (TI ("skin and soft tissue infection*" OR ssti* OR "skin infection*" OR "acute bacterial skin and skin structure infection*" OR absssi* OR "dermatological practice*" OR dermatolog* OR "skin care" OR "skin condition*" OR "skin disease*" OR "skin health" OR "skin problem*" OR "skin treat*" OR "skin*" OR "skin approach*" OR topical* OR (MM "Dermatologic Agents+") OR (MM "Soft Tissue Infections") OR (MM "Skin Diseases, Bacterial+")) OR AB ("skin and soft tissue infection*" OR ssti* OR "skin infection*" OR "acute bacterial skin and skin structure infection*" OR absssi* OR "dermatological practice*" OR dermatolog* OR "skin care" OR "skin condition*" OR "skin disease*" OR "skin health" OR "skin problem*" OR "skin treat*" OR "skin*" OR "skin approach*" OR topical* OR (MM "Dermatologic Agents+") OR (MM "Soft Tissue Infections") OR (MM "Skin Diseases, Bacterial+"))))	3	0

	Expanders - Apply equivalent subjects<br>Search modes - Boolean/Phrase		

Tl: title, AB: abstract, MM: exact major subject heading, MH: Exact subject heading, +: explode

F1000Research

https://f1000research.com

Search date: 4th Aug 2021

No	Search terms	Hits	After manual search
#1	wound AND cannabis	0 articles<br>1 faculty reviews<br>0 documents<br>0 posters<br>0 slides	0
#2	wound AND marijuana	0 articles<br>3 faculty reviews<br>0 documents<br>0 posters<br>0 slides	0
#3	wound AND cannabinoid	0 articles<br>2 faculty reviews<br>0 documents<br>0 posters<br>0 slides	0
#4	skin AND cannabis	2 articles<br>4 faculty reviews<br>0 documents<br>0 posters<br>0 slides	0
#5	skin AND marijuana	0 articles<br>3 faculty reviews<br>0 documents<br>0 posters<br>0 slides	0
#6	skin AND cannabinoid	1 articles<br>4 faculty reviews<br>0 documents<br>1 posters<br>0 slides	0
	Total	21	0

R_TI = title, R_ABS = abstract

Global Index Medicus

https://www.globalindexmedicus.net/

Search date: 4th Aug 2021

No	Search terms	Hits
#1	(tw:(cannabis OR hemp OR marijuana OR phytocannabinoid* OR cannabinoid*)) AND (tw:(wound OR skin))	38

tw: = title, abstract, subject

ISRCTN registry (BioMed central)

https://www.isrctn.com/

Supplementary material S1: Search strategies

Search date: 4th Aug 2021

No	Search terms	Hits	After manual search
#1	wound* AND (cannabis OR hemp OR marijuana OR phytocannabinoid* OR cannabinoid* OR cannabichromene* OR cannabicyclol* OR cannabidiol* OR cannabielsoin* OR cannabigerol* OR cannabinodiol* OR cannabinol* OR cannabitriol* OR tetrahydrocannabinol*)	1	1*
#2	skin* AND (cannabis OR hemp OR marijuana OR phytocannabinoid* OR cannabinoid* OR cannabichromene* OR cannabicyclol* OR cannabidiol* OR cannabielsoin* OR cannabigerol* OR cannabinodiol* OR cannabinol* OR cannabitriol* OR tetrahydrocannabinol*)	15	1**

*csv file downloaded. Article was searched using doi and included in endnote.
** this is similar to the article in search #1

NICE (National Institute for Health and Care Excellence)

https://www.nice.org.uk/

Search date: 4th Aug 2021

No	Search terms	Hits	After manual search
#1	wound AND cannabis	2<br>1 is research recommendations (https://www.nice.org.uk/about/what-we-do/science-policy-research/research-recommendations)<br>This is with 1705 results. Filtered with the term 'cannabis' resulted in 7 recommendations.	0
#2	wound AND marijuana	0	0
#3	wound AND cannabinoid	0	0
#4	skin AND cannabis	4<br>1 is research recommendations (https://www.nice.org.uk/about/what-we-do/science-policy-research/research-recommendations)<br>This is with 1705 results. Filtered with the term 'cannabis' resulted in 7 recommendations.	0
#5	skin AND marijuana	0	0
#6	skin AND cannabinoid	0	0
	Total	7	0

NDLTD (Networked Digital Library of Theses and Dissertations)

http://search.ndltd.org/

Search date: 4th Aug 2021

No	Search terms	Hits	After manual search
#1	wound AND cannabis	1	0
#2	wound AND marijuana	2	0
#3	wound AND cannabinoid	7	0
#4	skin AND cannabis	7	0
#5	skin AND marijuana	6	0
#6	skin AND cannabinoid	13	1
	Total	36	1

Supplementary material S1: Search strategies

OpenGrey

http://www.opengrey.eu/

Search date: 4th Aug 2021

No	Search	No of articles	After manual search
#1	(cannabis OR hemp OR marijuana OR phytocannabinoid OR cannabinoid OR cannabichromene OR cannabicyclol OR cannabidiol OR cannabielsoin OR cannabigerol OR cannabinodiol OR cannabinol OR cannabitriol OR tetrahydrocannabinol) AND ("skin and soft tissue infection" OR "skin and soft tissue infections" OR "skin infection" OR "skin infections" OR "acute bacterial skin and skin structure infection" OR "acute bacterial skin and skin structure infections" OR "dermatological practice" OR "dermatological practices" OR dermatology OR "skin care" OR "skin condition" OR "skin conditions" OR "skin disease" OR "skin diseases" OR "skin health" OR "skin problem" OR "skin problems" OR skin OR "skin approach" OR "skin approaches" OR topical OR wound)	3	0

ProQuest Dissertations & Theses A&I

Search date: 5th Aug 2021

No	Search	Hits
#1	(ti(cannabis OR hemp OR marijuana OR phytocannabinoid* OR cannabinoid* OR cannabichromene* OR cannabicyclol* OR cannabidiol* OR cannabielsoin* OR cannabigerol* OR cannabinodiol* OR cannabinol* OR cannabitriol* OR tetrahydrocannabinol*) OR ab(cannabis OR hemp OR marijuana OR phytocannabinoid* OR cannabinoid* OR cannabichromene* OR cannabicyclol* OR cannabidiol* OR cannabielsoin* OR cannabigerol* OR cannabinodiol* OR cannabinol* OR cannabitriol* OR tetrahydrocannabinol*)) AND ((ti(wound*) OR ab(wound*)) OR ((ti(antibacterial* OR “anti bacterial” OR “anti bacterials” OR “anti-bacterial” OR “anti-bacterials” OR bactericid* OR bacteriostatic* OR antiseptic* OR antibiotic*) OR ab(antibacterial* OR “anti bacterial” OR “anti bacterials” OR “anti-bacterial” OR “anti-bacterials” OR bactericid* OR bacteriostatic* OR antiseptic* OR antibiotic*)) AND (ti("skin and soft tissue infection" OR "skin and soft tissue infections" OR ssti* OR "skin infection" OR "skin infections" OR "acute bacterial skin and skin structure infection" OR OR "acute bacterial skin and skin structure infections" OR absssi* OR "dermatological practice" OR "dermatological practices" OR dermatolog* OR "skin care" OR "skin condition" OR "skin conditions" OR "skin disease" OR "skin diseases" OR "skin health" OR "skin problem" OR "skin problems" OR skin* OR "skin approach" OR "skin approaches" OR topical*) OR ab("skin and soft tissue infection" OR "skin and soft tissue infections" OR ssti* OR "skin infection" OR "skin infections" OR "acute bacterial skin and skin structure infection" OR OR "acute bacterial skin and skin structure infections" OR absssi* OR "dermatological practice" OR "dermatological practices" OR dermatolog* OR "skin care" OR "skin condition" OR "skin conditions" OR "skin disease" OR "skin diseases" OR "skin health" OR "skin problem" OR "skin problems" OR skin* OR "skin approach" OR "skin approaches" OR topical*))))	11

ti=title, ab=abstract

The Grey Literature Report

https://www.greylit.org/

Search date: 5th Aug 2021

No	Search terms	Hits	After manual search
#1	wound cannabis	0	0
#2	wound marijuana	0	0
#3	wound cannabinoid	0	0

#4	skin cannabis	0	0
#5	skin marijuana	0	0
#6	skin cannabinoid	0	0

Boolean operators are not supported. Search is by keyword and terms are AND together.

UK national research register

https://discovery.nationalarchives.gov.uk/browse/r/r/C17376

Search date: 5th Aug 2021

No	Search terms	Hits	After manual search
#1	wound AND (cannabis OR hemp OR marijuana OR "phytocannabinoid*" OR "cannabinoid*" OR "cannabichromene*" OR "cannabicyclol*" OR "cannabidiol*" OR "cannabielsoin*" OR "cannabigerol*" OR "cannabinodiol*" OR "cannabinol*" OR "cannabitriol*" OR "tetrahydrocannabinol*")	2	0
#2	skin AND (cannabis OR hemp OR marijuana OR "phytocannabinoid*" OR "cannabinoid*" OR "cannabichromene*" OR "cannabicyclol*" OR "cannabidiol*" OR "cannabielsoin*" OR "cannabigerol*" OR "cannabinodiol*" OR "cannabinol*" OR "cannabitriol*" OR "tetrahydrocannabinol*")	3	0
	Total	5	0

World Health Organization Institutional Repository for Information Sharing

(WHO IRIS, https://apps.who.int/iris/)

Search date: 10th Aug 2021

No	Search terms	Hits
#1	wound AND (cannabis OR hemp OR marijuana OR phytocannabinoid* OR cannabinoid* OR cannabichromene* OR cannabicyclol* OR cannabidiol* OR cannabielsoin* OR cannabigerol* OR cannabinodiol* OR cannabinol* OR cannabitriol* OR tetrahydrocannabinol*)	248
#2	skin AND (cannabis OR hemp OR marijuana OR phytocannabinoid* OR cannabinoid* OR cannabichromene* OR cannabicyclol* OR cannabidiol* OR cannabielsoin* OR cannabigerol* OR cannabinodiol* OR cannabinol* OR cannabitriol* OR tetrahydrocannabinol*)	465
	Total	713

(NLM) U.S. National Library of Medicine

https://www.nlm.nih.gov/

Search date: 10th Aug 2021

No	Search terms	Hits	After manual search
#1	wound AND (cannabis OR hemp OR marijuana OR phytocannabinoid* OR cannabinoid* OR cannabichromene* OR cannabicyclol* OR cannabidiol* OR cannabielsoin* OR cannabigerol* OR cannabinodiol* OR cannabinol* OR cannabitriol* OR tetrahydrocannabinol*)<br><br>filter: health information	1,952<br><br>11 (filtering)	0
#2	skin AND (cannabis OR hemp OR marijuana OR phytocannabinoid* OR cannabinoid* OR cannabichromene* OR cannabicyclol* OR cannabidiol* OR cannabielsoin* OR cannabigerol* OR cannabinodiol* OR cannabinol*	2,172	0

	OR cannabitriol* OR tetrahydrocannabinol*)<br><br>filter: health information	48	
	Total	59	0

DART Europe E-theses Portal

(https://www.dart-europe.org/basic-search.php)

Search date: 11th Aug 2021

No	Search terms	Hits	After manual search
#1	wound AND (cannabis OR hemp OR marijuana OR phytocannabinoid* OR cannabinoid* OR cannabichromene* OR cannabicyclol* OR cannabidiol* OR cannabielsoin* OR cannabigerol* OR cannabinodiol* OR cannabinol* OR cannabitriol* OR tetrahydrocannabinol*)	1	0
#2	skin AND (cannabis OR hemp OR marijuana OR phytocannabinoid* OR cannabinoid* OR cannabichromene* OR cannabicyclol* OR cannabidiol* OR cannabielsoin* OR cannabigerol* OR cannabinodiol* OR cannabinol* OR cannabitriol* OR tetrahydrocannabinol*)	14	2
	Total	15	2

SciFINDER

Search date: 3rd Aug 2021

No	Search terms	No of results
#1	antibacterial AND cannabinoid AND skin	Substances 0<br>References 30
#2	antibacterial AND phytocannabinoid AND skin	Substances 0<br>References 2
#3	cannabinoid AND wound	Substances 0<br>References 230
#4	phytocannabinoid AND wound	Substances 0<br>References 7
	Total	269

All downloaded in ris format and imported to endnote.

Journal search

Search dates: 27th, 28th Aug 2021

List of journals searched
Eastern Mediterranean Health Journal
Phytotherapy Research
Wiley online library
Journal of Inflammation
PLoS ONE
Dermatology journals
Acta Dermato-Venereologica
Acta Dermatovenerologica Alpina Pannonica Et Adriatica
Actas Dermo-Sifiliograficas
American Journal of Clinical Dermatology
Anais Brasileiros de Dermatologia

Supplementary material S1: Search strategies

Annals of Dermatology
Archives of Dermatological Research
JAMA Dermatology
Australasian Journal of Dermatology
British Journal of Dermatology
Case Reports in Dermatology
Clinical, Cosmetic & Investigational Dermatology
Clinical and Experimental Dermatology
Clinics in Dermatology
CUTIS
Dermatologic Clinics
Dermatologic Surgery
Dermatologic Therapy
Dermatology
Dermatology Practical & Conceptual
Dermatology Research & Practice
Dermatology Reports
European Journal of Dermatology
Experimental Dermatology
International Wound Journal
Wound Repair and Regeneration
phytotherapy research
journal of herbal medicine
phytomedicine
Journal of Pharmacy & Pharmacognosy Research

Additional Bibliography Review

In addition to the above literature searches, a thorough search of bibliographies from the articles which met the inclusion criteria and additional sources was conducted as part of the grey literature search to complete this systematic review. The following list contains the articles in which the hand search was conducted.

1. Baswan SM, Klosner AE, Glynn K, et al. Therapeutic potential of cannabidiol (CBD) for skin health and disorders. Clinical, Cosmetic and Investigational Dermatology 2020;13:927-942.
2. Chingwaru C, Bagar T, Maroyi A, Kapewangolo PT, Chingwaru W. Wound healing potential of selected Southern African medicinal plants: A review. Journal of Herbal Medicine 2019;17-18, 100263.
3. Copeland-Halperin LR, Herrera-Gomez LC, LaPier JR, Shank N, Shin JH. The Effects of Cannabis: Implications for the surgical patient. Plast Reconstr Surg Glob Open 2021;9(3):e3448.
4. Dhadwal G, Kirchhof MG. The Risks and Benefits of Cannabis in the Dermatology Clinic. J Cutan Med Surg 2018;22(2):194-199.
5. Farahani RMZ. Endocannabinoid system and wound healing. Journal of Tissue Viability 2008;17(3):100-101.
6. Eagelston LRM, Yazd NKK, Patel RR, Flaten HK, Dunnick CA, Dellavalle RP. Cannabinoids in dermatology: a scoping review. Dermatology Online Journal 2018;24(6),1-17.
7. Goncalves J, Rosado T, Soares S, et al. Cannabis and its secondary metabolites: Their use as therapeutic drugs, toxicological aspects, and analytical determination. Medicines (Basel) 2019;6(1):31.

8. Mansouri K, Norooznezhad AH. Cannabinoids: A possible treatment for chronic cutaneous wounds. J Dermatolog Treat 2021;32(1):128-129.
9. Shao K, Stewart C, Grant-Kels JM. Cannabis and the skin. Clinics in Dermatology 2021;39(5):784-795.
10. Weigelt MA, Sivamani R, Lev-Tov H. The therapeutic potential of cannabinoids for integumentary wound management. Experimental Dermatology 2021;30(2):201-211.
11. Maida V, Shi RB, Fazzari FGT, Zomparelli LM. A new treatment paradigm for sickle cell disease leg ulcers: Topical cannabis-based medicines. Experimental Dermatology 2021;30(2):291-293.
12. Maida V. Medical Cannabis in the Palliation of Malignant Wounds—A Case Report. Journal of Pain and Symptom Management 2017;53(1):e4-e6.
13. Kibret BG, Patel S, Niezgoda J, Guns W, Niezgoda J, Gopalakrishnan S, Baban B, Cubillos P, Villeneuve D, Kumar P. Evidence-based Potential Therapeutic Applications of Cannabinoids in Wound Management. Adv Skin Wound Care. 2022;35(8):447-453. doi: 10.1097/01.ASW.0000831920.15801.25.
14. Schofs, L.; Sparo, M.D.; Sánchez Bruni, S.F. The antimicrobial effect behind Cannabis sativa. Pharmacol. Res. Perspect. 2021;9:e00761.
15. Ferreira BP, Costa G, Mascarenhas-Melo F, et al. Skin applications of cannabidiol: sources, effects, delivery systems, marketed formulations and safety. Phytochemistry Reviews 2023;22(3):781-828. DOI: 10.1007/s11101-023-09860-5
16. Healy CR, Gethin G, Pandit A, Finn DP. Chronic wound-related pain, wound healing and the therapeutic potential of cannabinoids and endocannabinoid system modulation. Biomedicine and Pharmacotherapy 2023;168. DOI: 10.1016/j.biopha.2023.115714
17. Makhakhe L. Topical cannabidiol (CBD) in skin pathology -- A comprehensive review and prospects for new therapeutic opportunities. South African Family Practice 2022;64(1):a5493. DOI: 10.4102/safp.v64i1.5493
18. Martinelli G, Magnavacca A, Fumagalli M, Dell'Agli M, Piazza S, Sangiovanni E. Cannabis sativa and skin health: Dissecting the role of phytocannabinoids. 2022; 88: 492–506 (http://hdl.handle.net/2434/897955)
19. Niezgoda J, Kimball T, Gen. Cannabis, Cannabinoids, & Wound Care: Where There's Smoke There's Fire. American Professional Wound Care Association Unpublished; 2023
20. Parikh AC, Jeffery CS, Sandhu Z, Brownlee BP, Queimado L, Mims MM. The effect of cannabinoids on wound healing: A review. Health Science Reports 2024;7(2) e1908. DOI: 10.1002/hsr2.1908
21. Ramer R, Hinz B. Cannabinoid Compounds as a Pharmacotherapeutic Option for the Treatment of Non-Cancer Skin Diseases. Cells 2022;11(24) 4102. DOI: 10.3390/cells11244102
22. Shao K, Grant-Kels JM, Stewart C. The impact of cannabis and cannabinoids on the skin. Cannabis Use, Neurobiology, Psychology, and Treatment: Elsevier; 2023:525-539.
23. Soares MGdS, Silva MEWdB, Barbosa MLCdS, et al. The use of cannabinoids in dermatological pathophysiologies: a systematic review ; El uso de cannabinoides en fisiopatología dermatológica: una revisión sistemática ; O uso de canabinoides em fisiopatologias dermatológicas: uma revisão sistemática. 2022; 11(2): e55411225961 (https://rsdjournal.org/index.php/rsd/article/view/25961)

1 Updated search

CINAHL Plus with Full Text (EBSCO)

Search date: 20.05.2024 (from June 2021-todate)

No	Search	No of

Supplementary material S1: Search strategies

		articles
#1	(TI (cannabis OR hemp OR marijuana OR phytocannabinoid* OR cannabinoid* OR cannabichromene* OR cannabicyclol* OR cannabidiol* OR cannabielsoin* OR cannabigerol* OR cannabinodiol* OR cannabinol* OR cannabitriol* OR tetrahydrocannabinol* OR (MM "Medical Marijuana") OR (MM "Cannabinoids+")) OR AB (cannabis OR hemp OR marijuana OR phytocannabinoid* OR cannabinoid* OR cannabichromene* OR cannabicyclol* OR cannabidiol* OR cannabielsoin* OR cannabigerol* OR cannabinodiol* OR cannabinol* OR cannabitriol* OR tetrahydrocannabinol* OR (MM "Medical Marijuana") OR (MM "Cannabinoids+"))) AND ((TI ("wound*" OR "wound heal*" OR (MM "Wound Healing+") OR (MM "Wound Infection+")) OR AB ("wound*" OR "wound heal*" OR (MM "Wound Healing+") OR (MM "Wound Infection+"))) OR (TI (antibacterial* OR “anti bacterial*” OR “anti-bacterial” OR bactericid* OR bacteriostatic* OR antiseptic OR antibiotic OR (MM "Anti-Bacterial Agents+")) OR AB (antibacterial* OR “anti bacterial*” OR “anti-bacterial” OR bactericid* OR bacteriostatic* OR antiseptic* OR antibiotic* OR (MM "Anti-Bacterial Agents+"))) AND (TI ("skin and soft tissue infection*" OR ssti* OR "skin infection*" OR "acute bacterial skin and skin structure infection*" OR absssi* OR "dermatological practice*" OR dermatolog* OR "skin care" OR "skin condition*" OR "skin disease*" OR "skin health" OR "skin problem*" OR "skin treat*" OR "skin*" OR "skin approach*" OR topical* OR (MM "Dermatologic Agents+") OR (MM "Soft Tissue Infections") OR (MM "Skin Diseases, Bacterial+")) OR AB ("skin and soft tissue infection*" OR ssti* OR "skin infection*" OR "acute bacterial skin and skin structure infection*" OR absssi* OR "dermatological practice*" OR dermatolog* OR "skin care" OR "skin condition*" OR "skin disease*" OR "skin health" OR "skin problem*" OR "skin treat*" OR "skin*" OR "skin approach*" OR topical* OR (MM "Dermatologic Agents+") OR (MM "Soft Tissue Infections") OR (MM "Skin Diseases, Bacterial+"))))	16

Tl: title, AB: abstract, MM: exact major subject heading, MH: Exact subject heading, +: explode

Medline via EBSCOhost

Search date: 20.05.2024 (from June 2021-todate)

No	Search	No of articles
#1	(TI (cannabis OR hemp OR marijuana OR phytocannabinoid* OR cannabinoid* OR cannabichromene* OR cannabicyclol* OR cannabidiol* OR cannabielsoin* OR cannabigerol* OR cannabinodiol* OR cannabinol* OR cannabitriol* OR tetrahydrocannabinol* OR (MM "Medical Marijuana") OR (MM "Cannabinoids+")) OR AB (cannabis OR hemp OR marijuana OR phytocannabinoid* OR cannabinoid* OR cannabichromene* OR cannabicyclol* OR cannabidiol* OR cannabielsoin* OR cannabigerol* OR cannabinodiol* OR cannabinol* OR cannabitriol* OR tetrahydrocannabinol* OR (MM "Medical Marijuana") OR (MM "Cannabinoids+"))) AND ((TI ("wound*" OR "wound heal*" OR (MM "Wound Healing+") OR (MM "Wound Infection+")) OR AB ("wound*" OR "wound heal*" OR (MM "Wound Healing+") OR (MM "Wound Infection+"))) OR (TI (antibacterial* OR “anti bacterial*” OR “anti-bacterial” OR bactericid* OR bacteriostatic* OR antiseptic OR antibiotic OR (MM "Anti-Bacterial Agents+")) OR AB (antibacterial* OR “anti bacterial*” OR “anti-bacterial” OR bactericid* OR bacteriostatic* OR antiseptic* OR antibiotic* OR (MM "Anti-Bacterial Agents+"))) AND (TI ("skin and soft tissue infection*" OR ssti* OR "skin infection*" OR "acute bacterial skin and skin structure infection*" OR absssi* OR "dermatological practice*" OR dermatolog* OR "skin care" OR "skin condition*" OR "skin disease*" OR "skin health" OR "skin problem*" OR "skin treat*" OR "skin*" OR "skin approach*" OR topical* OR (MM "Dermatologic Agents+") OR (MM "Soft Tissue Infections") OR (MM "Skin Diseases, Bacterial+")) OR AB ("skin and soft tissue infection*" OR ssti* OR "skin infection*" OR "acute bacterial skin and skin structure infection*" OR absssi* OR "dermatological practice*" OR dermatolog* OR "skin care" OR "skin condition*" OR "skin disease*" OR "skin health" OR "skin problem*" OR "skin treat*" OR "skin*" OR "skin	87

Supplementary material S1: Search strategies

	approach*" OR topical* OR (MM "Dermatologic Agents+") OR (MM "Soft Tissue Infections") OR (MM "Skin Diseases, Bacterial+"))))	

Tl: title, AB: abstract, MM: exact major subject heading, MH: Exact subject heading, +: explode

Scopus

Search date: 20.05.2024 (2021-todate)

No	Search	No of articles
#1	TITLE-ABS-KEY (cannabis OR hemp OR marijuana OR phytocannabinoid* OR cannabinoid* OR cannabichromene* OR cannabicyclol* OR cannabidiol* OR cannabielsoin* OR cannabigerol* OR cannabinodiol* OR cannabinol* OR cannabitriol* OR tetrahydrocannabinol*)	121,923
#2	TITLE-ABS-KEY ("wound*" OR "wound heal*")	655,312
#3	TITLE-ABS-KEY (antibacterial* OR "anti bacterial*" OR "anti-bacterial" OR bactericid* OR bacteriostatic* OR antiseptic* OR antibiotic*)	1,359,719
#4	TITLE-ABS-KEY ("skin and soft tissue infection*" OR ssti* OR "skin infection*" OR "acute bacterial skin and skin structure infection*" OR absssi* OR "dermatological practice*" OR dermatolog* OR "skin care" OR "skin condition*" OR "skin disease*" OR "skin health" OR "skin problem*" OR "skin treat*" OR "skin*" OR "skin approach*" OR topical*)	1,808,867
#5	#1 AND #2	793
#6	#1 AND #3 AND #4	130
#7	#5 0R #6<br>((TITLE-ABS-KEY (cannabis OR hemp OR marijuana OR phytocannabinoid* OR cannabinoid* OR cannabichromene* OR cannabicyclol* OR cannabidiol* OR cannabielsoin* OR cannabigerol* OR cannabinodiol* OR cannabinol* OR cannabitriol* OR tetrahydrocannabinol*)) AND (TITLE-ABS-KEY ("wound*" OR "wound heal*"))) OR ((TITLE-ABS-KEY (cannabis OR hemp OR marijuana OR phytocannabinoid* OR cannabinoid* OR cannabichromene* OR cannabicyclol* OR cannabidiol* OR cannabielsoin* OR cannabigerol* OR cannabinodiol* OR cannabinol* OR cannabitriol* OR tetrahydrocannabinol*)) AND (TITLE-ABS-KEY (antibacterial* OR "anti bacterial*" OR "anti-bacterial" OR bactericid* OR bacteriostatic* OR antiseptic* OR antibiotic*)) AND (TITLE-ABS-KEY ("skin and soft tissue infection*" OR ssti* OR "skin infection*" OR "acute bacterial skin and skin structure infection*" OR absssi* OR "dermatological practice*" OR dermatolog* OR "skin care" OR "skin condition*" OR "skin disease*" OR "skin health" OR "skin problem*" OR "skin treat*" OR "skin*" OR "skin approach*" OR topical*)))	900<br>When period is adjusted from 2021-2024<br>314

TITLE-ABS-KEY : article title, abstract, keywords

PubMed

Search date: 20.05.2024 (2021-todate)

No	Search	No of articles
#1	"cannabis"[Title/Abstract] OR "hemp"[Title/Abstract] OR "marijuana"[Title/Abstract] OR "phytocannabinoid*"[Title/Abstract] OR "cannabinoid*"[Title/Abstract] OR "cannabichromene*"[Title/Abstract] OR "cannabicyclol*"[Title/Abstract] OR "cannabidiol*"[Title/Abstract] OR "cannabielsoin*"[Title/Abstract] OR "cannabigerol*"[Title/Abstract] OR "cannabinodiol*"[Title/Abstract] OR "cannabinol*"[Title/Abstract] OR "cannabitriol*"[Title/Abstract] OR "tetrahydrocannabinol*"[Title/Abstract] OR "cannabinoids"[MeSH Terms] OR "medical marijuana"[MeSH Terms]	67,037
#2	"wound*"[Title/Abstract] OR "wound heal*"[Title/Abstract] OR "Wound Healing"[MeSH Terms] OR "Wound Infection"[MeSH Terms]	397,576

#3	"antibacterial*"[Title/Abstract] OR "anti bacterial*"[Title/Abstract] OR "anti-bacterial"[Title/Abstract] OR "bactericid*"[Title/Abstract] OR "bacteriostatic*"[Title/Abstract] OR "antiseptic*"[Title/Abstract] OR "antibiotic*"[Title/Abstract] OR "Anti-Bacterial Agents"[MeSH Terms]	786,779
#4	"skin and soft tissue infection*"[Title/Abstract] OR "ssti*"[All Fields] OR "skin infection*"[Title/Abstract] OR "acute bacterial skin and skin structure infection*"[Title/Abstract] OR "absssi*"[All Fields] OR "dermatological practice*"[Title/Abstract] OR "dermatolog*"[All Fields] OR "skin care"[Title/Abstract] OR "skin condition*"[Title/Abstract] OR "skin disease*"[Title/Abstract] OR "skin health"[Title/Abstract] OR "skin problem*"[Title/Abstract] OR "skin treat*"[Title/Abstract] OR "skin*"[Title/Abstract] OR "skin approach*"[Title/Abstract] OR "topical*"[All Fields] OR "Dermatologic Agents"[MeSH Terms] OR "Soft Tissue Infections"[MeSH Terms]	1,085,901
#5	#3 AND #4	63,067
#6	#2 OR #5	449,840
#7	#1 AND #6	256<br>After setting period to 2021-2024:<br>114

Web of Science (WoS)

Search date: 20.05.2024 (2021-todate)

**No**	**Search**	**No of articles**
#1	TS=(cannabis OR hemp OR marijuana OR phytocannabinoid* OR cannabinoid* OR cannabichromene* OR cannabicyclol* OR cannabidiol* OR cannabielsoin* OR cannabigerol* OR cannabinodiol* OR cannabinol* OR cannabitriol* OR tetrahydrocannabinol*)	133,602
#2	TS=("wound*" OR "wound heal*")	659,822
#3	TS=(antibacterial* OR “anti bacterial*” OR “anti-bacterial” OR bactericid* OR bacteriostatic* OR antiseptic* OR antibiotic*)	1,501,283
#4	TS=("skin and soft tissue infection*" OR ssti* OR "skin infection*" OR "acute bacterial skin and skin structure infection*" OR absssi* OR "dermatological practice*" OR dermatolog* OR "skin care" OR "skin condition*" OR "skin disease*" OR "skin health" OR "skin problem*" OR "skin treat*" OR "skin*" OR "skin approach*" OR topical*)	1,959,400
#5	#3 AND #4	113,755
#6	#2 OR #5	753,803
#7	#1 AND #6	831<br>After setting period to 2021-2024:<br>240

TS: topic (title, abstract and key words)

Cochrane library

Search date: 20.05.2024 (2021-todate)

**No**	**Search**	**No of articles**

Supplementary material S1: Search strategies

#1	(cannabis OR hemp OR marijuana OR phytocannabinoid* OR cannabinoid* OR cannabichromene* OR cannabicyclol* OR cannabidiol* OR cannabielsoin* OR cannabigerol* OR cannabinodiol* OR cannabinol* OR cannabitriol* OR tetrahydrocannabinol*):ti,ab,kw	6,050
#2	MeSH descriptor: [Cannabinoids] explode all trees	1,531
#3	MeSH descriptor: [Medical Marijuana] explode all trees	55
#4	#1 OR #2 OR #3	6,144
#5	(wound* OR wound NEXT heal*):ti,ab,kw	40,362
#6	MeSH descriptor: [Wound Healing] explode all trees	8,208
#7	MeSH descriptor: [Wound Infection] explode all trees	5,177
#8	#5 OR #6 OR #7	32,627
#9	(antibacterial* OR anti NEXT bacterial* OR anti-bacterial OR bactericid* OR bacteriostatic* OR antiseptic* OR antibiotic*):ti,ab,kw	49,788
#10	MeSH descriptor: [Anti-Bacterial Agents] explode all trees	16,890
#11	#9 OR #10	51,025
#12	(skin and soft NEXT tissue NEXT infection* OR SSTI* OR skin NEXT infection* OR acute NEXT bacterial NEXT skin and skin NEXT structure NEXT infection* OR ABSSSI* OR dermatological NEXT practice* OR dermatolog* OR skin NEXT care OR skin NEXT condition* OR skin NEXT disease* OR skin NEXT health OR skin NEXT problem* OR skin NEXT treat* OR skin* OR skin NEXT approach* OR topical*):ti,ab,kw	109,485
#13	MeSH descriptor: [Dermatologic Agents] explode all trees	4,859
#14	MeSH descriptor: [Soft Tissue Infections] explode all trees	190
#15	#12 OR #13 OR #14	110,586
#16	#11 AND #15	6,908
#17	#8 OR #16	46,986
#18	#1 AND #17	27 (trials) After setting period to 2021-2024: 12

ti,ab,kw: title, abstract and key words

(LILACS) Latin America and Caribbean health Sciences Literature

https://lilacs.bvsalud.org/en/

Search date: 20.05.2024 (2021-todate)

No	Search	No of articles
#1	cannabis OR hemp OR marijuana OR phytocannabinoid OR cannabinoid OR cannabichromene OR cannabicyclol OR cannabidiol OR cannabielsoin OR cannabigerol OR cannabinodiol OR cannabinol OR cannabitriol OR tetrahydrocannabinol [Title, abstract, subject]	73,403
#2	wound OR wound heal [Title, abstract, subject]	17,258
#3	antibacterial OR antibacterial OR antibacterial OR bactericid OR bacteriostatic OR antiseptic OR antibiotic [Title, abstract, subject]	849,460
#4	soft tissue infection OR ssti OR skin infection OR absssi OR dermatological practice OR dermatolog OR skin care OR skin condition OR skin disease OR skin health OR skin problem OR skin treat OR skin OR skin approach OR topical [Title, abstract, subject]	201,787
#5	#3 AND #4<br>(antibacterial OR antibacterial OR antibacterial OR bactericid OR bacteriostatic OR antiseptic OR antibiotic) AND (soft tissue infection OR ssti OR skin infection OR absssi OR dermatological practice OR dermatolog OR skin care OR skin condition OR skin disease OR skin health OR skin problem OR skin treat OR skin OR skin approach OR topical)	9,227

#6	#2 OR #5<br>(wound OR wound heal) OR ((antibacterial OR antibacterial OR antibacterial OR bactericid OR bacteriostatic OR antiseptic OR antibiotic) AND (soft tissue infection OR ssti OR skin infection OR absssi OR dermatological practice OR dermatolog OR skin care OR skin condition OR skin disease OR skin health OR skin problem OR skin treat OR skin OR skin approach OR topical))	26,436
#7	#1 AND #6<br>(cannabis OR hemp OR marijuana OR phytocannabinoid OR cannabinoid OR cannabichromene OR cannabicyclol OR cannabidiol OR cannabielsoin OR cannabigerol OR cannabinodiol OR cannabinol OR cannabitriol OR tetrahydrocannabinol) AND ((wound OR wound heal) OR ((antibacterial OR antibacterial OR antibacterial OR bactericid OR bacteriostatic OR antiseptic OR antibiotic) AND (soft tissue infection OR ssti OR skin infection OR absssi OR dermatological practice OR dermatolog OR skin care OR skin condition OR skin disease OR skin health OR skin problem OR skin treat OR skin OR skin approach OR topical)))	14<br>After setting period to 2021-2024:<br>8<br>Hand searching results in 1 (common with Medline)

Grey literature

AHRQ (The Agency for Healthcare Research and Quality)

(AHRQ, https://www.ahrq.gov/)

Search date: 21.05.2024

No	Search terms	Hits	After manual search
#1	wound cannabis<br>• Searched entire documents<br>• At least one of these words "wound cannabis"<br>• These file types only "application/pdf"	2,867	0
#2	wound marijuana<br>• Searched entire documents<br>• At least one of these words "wound marijuana"<br>• These file types only "application/pdf"	2,942	0
#3	wound cannabinoid<br>• Searched entire documents<br>• At least one of these words "wound cannabinoid"<br>• These file types only "application/pdf"	2,792	0
#4	skin cannabis<br>• Searched entire documents<br>• At least one of these words "skin cannabis"<br>• These file types only "application/pdf"	3,514	0
#5	skin marijuana<br>• Searched entire documents<br>• At least one of these words "skin marijuana"<br>• These file types only "application/pdf"	3,543	0
#6	Skin cannabinoid<br>• Searched entire documents<br>• At least one of these words "skin cannabinoid"<br>• These file types only "application/pdf"	3,453	0

BASE (Bielefeld Academic Search Engine)

(BASE, https://www.base-search.net/)

Supplementary material S1: Search strategies

Search dates: 21.05.2024 (2021-todate)

No	Search terms	Hits
#1	(wound skin) AND (cannabis marijuana) year:[2021 TO 2024]<br>Entire document searched	463
#2	cannabinoid* (wound skin) year:[2021 TO 2024]<br>Entire document searched	504

EBSCOhost Open Dissertations

https://biblioboard.com/opendissertations/

Search date: 21.05.2024 (2021-todate)

No	Search	No of articles	After manual search
#1	TI (cannabis OR hemp OR marijuana OR phytocannabinoid* OR cannabinoid* OR cannabichromene* OR cannabicyclol* OR cannabidiol* OR cannabielsoin* OR cannabigerol* OR cannabinodiol* OR cannabinol* OR cannabitriol* OR tetrahydrocannabinol* OR (MM "Medical Marijuana") OR (MM "Cannabinoids+")) OR AB (cannabis OR hemp OR marijuana OR phytocannabinoid* OR cannabinoid* OR cannabichromene* OR cannabicyclol* OR cannabidiol* OR cannabielsoin* OR cannabigerol* OR cannabinodiol* OR cannabinol* OR cannabitriol* OR tetrahydrocannabinol* OR (MM "Medical Marijuana") OR (MM "Cannabinoids+"))<br>Limitations - Publication Date: 20210101-20241231<br>Expanders - Apply equivalent subjects<br>Search modes - Boolean/Phrase	310	
#2	TI ("wound*" OR "wound heal*" OR (MM "Wound Healing+") OR (MM "Wound Infection+")) OR AB ("wound*" OR "wound heal*" OR (MM "Wound Healing+") OR (MM "Wound Infection+"))) OR (TI (antibacterial* OR “anti bacterial*” OR “anti-bacterial” OR bactericid* OR bacteriostatic* OR antiseptic OR antibiotic OR (MM "Anti-Bacterial Agents+")) OR AB (antibacterial* OR “anti bacterial*” OR “anti-bacterial” OR bactericid* OR bacteriostatic* OR antiseptic* OR antibiotic* OR (MM "Anti-Bacterial Agents+")<br>Limitations - Publication Date: 20210101-20241231<br>Expanders - Apply equivalent subjects<br>Search modes - Boolean/Phrase	1,988	
#3	TI ("skin and soft tissue infection*" OR ssti* OR "skin infection*" OR "acute bacterial skin and skin structure infection*" OR absssi* OR "dermatological practice*" OR dermatolog* OR "skin care" OR "skin condition*" OR "skin disease*" OR "skin health" OR "skin problem*" OR "skin treat*" OR "skin*" OR "skin approach*" OR topical* OR (MM "Dermatologic Agents+") OR (MM "Soft Tissue Infections") OR (MM "Skin Diseases, Bacterial+")) OR AB ("skin and soft tissue infection*" OR ssti* OR "skin infection*" OR "acute bacterial skin and skin structure infection*" OR absssi* OR "dermatological practice*" OR dermatolog* OR "skin care" OR "skin condition*" OR "skin disease*" OR "skin health" OR "skin problem*" OR "skin treat*" OR "skin*" OR "skin approach*" OR topical* OR (MM "Dermatologic Agents+") OR (MM "Soft Tissue Infections") OR (MM "Skin Diseases, Bacterial+")))<br>Limitations - Publication Date: 20210101-20241231<br>Expanders - Apply equivalent subjects<br>Search modes - Boolean/Phrase	1,551	
#4	#2 OR #3	3,366	
#5	#1 AND #4	7	**0**

Tl: title, AB: abstract, MM: exact major subject heading, MH: Exact subject heading, +: explode

Supplementary material S1: Search strategies

F1000Research

https://f1000research.com

Search date: 21.05.2024

No	Search terms	Hits	After manual search
#1	wound AND cannabis	2 articles<br>1 faculty reviews<br>0 documents<br>0 posters<br>0 slides	0
#2	wound AND marijuana	0 articles<br>3 faculty reviews<br>0 documents<br>0 posters<br>0 slides	0
#3	wound AND cannabinoid	1 articles<br>2 faculty reviews<br>0 documents<br>0 posters<br>0 slides	0
#4	skin AND cannabis	6 articles<br>4 faculty reviews<br>0 documents<br>0 posters<br>0 slides	0
#5	skin AND marijuana	2 articles<br>3 faculty reviews<br>0 documents<br>0 posters<br>0 slides	0
#6	skin AND cannabinoid	5 articles<br>4 faculty reviews<br>0 documents<br>1 posters<br>0 slides	0
	Total	21	0

R_TI = title, R_ABS = abstract

Global Index Medicus

https://www.globalindexmedicus.net/

Search date: 21.05.2024 (period: 2021-2024)

No	Search terms	Hits
#1	(tw:(cannabis OR hemp OR marijuana OR phytocannabinoid* OR cannabinoid*)) AND (tw:(wound OR skin))	5

tw: = title, abstract, subject

ISRCTN registry (BioMed central)

https://www.isrctn.com/

Search date: 21.05.2024

Supplementary material S1: Search strategies

No	Search terms	Hits	After manual search
#1	wound* AND (cannabis OR hemp OR marijuana OR phytocannabinoid* OR cannabinoid* OR cannabichromene* OR cannabicyclol* OR cannabidiol* OR cannabielsoin* OR cannabigerol* OR cannabinodiol* OR cannabinol* OR cannabitriol* OR tetrahydrocannabinol*) within date applied from 01.07.2021 to 31.12.2024	2	0
#2	skin* AND (cannabis OR hemp OR marijuana OR phytocannabinoid* OR cannabinoid* OR cannabichromene* OR cannabicyclol* OR cannabidiol* OR cannabielsoin* OR cannabigerol* OR cannabinodiol* OR cannabinol* OR cannabitriol* OR tetrahydrocannabinol*)	18	0

NICE (National Institute for Health and Care Excellence)

https://www.nice.org.uk/

Search date: 21.05.2024 (last updated: last 3 years)

No	Search terms	Hits	After manual search
#1	wound AND cannabis	0	0
#2	wound AND marijuana	0	0
#3	wound AND cannabinoid	0	0
#4	skin AND cannabis	1	0
#5	skin AND marijuana	0	0
#6	skin AND cannabinoid	0	0

NDLTD (Networked Digital Library of Theses and Dissertations)

http://search.ndltd.org/

Search date: 21.05.2024 (period: 2021-2024)

No	Search terms	Hits	After manual search
#1	wound AND cannabis	0	0
#2	wound AND marijuana	0	0
#3	wound AND cannabinoid	0	0
#4	skin AND cannabis	3	0
#5	skin AND marijuana	0	0
#6	skin AND cannabinoid	2	0

OpenGrey

http://www.opengrey.eu/

Search date: 21.05.2024

No	Search	No of articles	After manual search
#1	cannabis OR hemp OR marijuana OR phytocannabinoid OR cannabinoid OR cannabichromene OR cannabicyclol OR cannabidiol OR cannabielsoin OR cannabigerol OR cannabinodiol OR cannabinol OR cannabitriol OR tetrahydrocannabinol	**0**	**0**
#2	("skin and soft tissue infection" OR "skin and soft tissue infections" OR "skin infection" OR "skin infections" OR "acute bacterial skin and skin structure infection" OR "acute bacterial skin and skin structure infections" OR "dermatological practice" OR "dermatological practices" OR dermatology OR	**3**	**0**

	"skin care" OR "skin condition" OR "skin conditions" OR "skin disease" OR "skin diseases" OR "skin health" OR "skin problem" OR "skin problems" OR skin OR "skin approach" OR "skin approaches" OR topical OR wound		

ProQuest Dissertations & Theses A&I

Search date: 21.05.2024 (period: 01.08.2021-31.12.2024)

No	Search	Hits	Manual search
#1	(ti(cannabis OR hemp OR marijuana OR phytocannabinoid* OR cannabinoid* OR cannabichromene* OR cannabicyclol* OR cannabidiol* OR cannabielsoin* OR cannabigerol* OR cannabinodiol* OR cannabinol* OR cannabitriol* OR tetrahydrocannabinol*) OR ab(cannabis OR hemp OR marijuana OR phytocannabinoid* OR cannabinoid* OR cannabichromene* OR cannabicyclol* OR cannabidiol* OR cannabielsoin* OR cannabigerol* OR cannabinodiol* OR cannabinol* OR cannabitriol* OR tetrahydrocannabinol*)) AND ((ti(wound*) OR ab(wound*)) OR ((ti(antibacterial* OR “anti bacterial” OR “anti bacterials” OR “anti-bacterial” OR “anti-bacterials” OR bactericid* OR bacteriostatic* OR antiseptic* OR antibiotic*) OR ab(antibacterial* OR “anti bacterial” OR “anti bacterials” OR “anti-bacterial” OR “anti-bacterials” OR bactericid* OR bacteriostatic* OR antiseptic* OR antibiotic*)) AND (ti("skin and soft tissue infection" OR "skin and soft tissue infections" OR ssti* OR "skin infection" OR "skin infections" OR "acute bacterial skin and skin structure infection" OR OR "acute bacterial skin and skin structure infections" OR absssi* OR "dermatological practice" OR "dermatological practices" OR dermatolog* OR "skin care" OR "skin condition" OR "skin conditions" OR "skin disease" OR "skin diseases" OR "skin health" OR "skin problem" OR "skin problems" OR skin* OR "skin approach" OR "skin approaches" OR topical*) OR ab("skin and soft tissue infection" OR "skin and soft tissue infections" OR ssti* OR "skin infection" OR "skin infections" OR "acute bacterial skin and skin structure infection" OR OR "acute bacterial skin and skin structure infections" OR absssi* OR "dermatological practice" OR "dermatological practices" OR dermatolog* OR "skin care" OR "skin condition" OR "skin conditions" OR "skin disease" OR "skin diseases" OR "skin health" OR "skin problem" OR "skin problems" OR skin* OR "skin approach" OR "skin approaches" OR topical*))))	53	7

ti=title, ab=abstract
*Found in other studies

1	U.S. patent and trademark office publishes TF holdings's patent application for wound treatment topical composition containing cannabinoids. (2024/02/12/, 2024 Feb 12). *Global IP News.Medical Patent News* Retrieved from https://ezproxy.canberra.edu.au/login?url=https://www.proquest.com/wire-feeds/u-s-patent-trademark-office-publishes-tf/docview/2925435638/se-2
2	Kim, E., Jang, J., Seo, H. H., Lee, J. H., & Moh, S. H. (2024). *Cannabis sativa (hemp) seed-derived peptides WVYY and PSLPA modulate the Nrf2 signaling pathway in human keratinocytes*. Cold Spring Harbor: doi:https://doi.org/10.1101/2024.01.26.577509
3	Kilner, J. (2023/07/13/, 2023 Jul 13). Ukraine will legalise cannabis to help war wounded cope with trauma. *Telegraph.Co.Uk* Retrieved from https://ezproxy.canberra.edu.au/login?url=https://www.proquest.com/newspapers/ukraine-will-legalise-cannabis-help-war-wounded/docview/2836632553/se-2
4	Zarei, A., Behdarvandi, B., Dinani, E. T., & Maccarone, J. (2021/12//). Cannabis sativa L photoautotrophic micropropagation: A powerful tool for industrial scale in vitro propagation. *In Vitro Cellular & Developmental Biology, 57*(6), 932-941. doi:https://doi.org/10.1007/s11627-021-10167-3
5	Golovin, V. (2023/12/22/). *Wounded ukrainian soldiers find hope to ease pain after parliament legalizes medical cannabis: Medical cannabis could also be used to treat post-traumatic stress disorder – a problem not only for soldiers but for civilians frequently subjected to drone and missile attacks*. Toronto: The Globe and Mail. Retrieved from https://ezproxy.canberra.edu.au/login?url=https://www.proquest.com/blogs-podcasts-websites/wounded-ukrainian-soldiers-find-hope-ease-pain/docview/2904610114/se-2
6	Fu, R. (2022). *Cannabidiol and ascorbic acid promotes epithelial cell migration in an in vitro model of wound*

	healing (Order No. 30247055). Available from ProQuest Dissertations & Theses Global: The Sciences and Engineering Collection. (2774848361). Retrieved from https://ezproxy.canberra.edu.au/login?url=https://www.proquest.com/dissertations-theses/cannabidiol-ascorbic-acid-promotes-epithelial/docview/2774848361/se-2
7	INTERNATIONAL PATENT: PFNATURE CO., LTD., 주식회사 피에프네이처 FILES APPLICATION FOR "COMPOSITION FOR ANTI-BACTERIAL, ANTI-INFLAMMATORY AND SKIN WRINKLE IMPROVEMENT, COMPRISING FERMENTED HEMP STEM EXTRACT AS ACTIVE INGREDIENT". (2024/01/16/, 2024 Jan 16). *US Fed News Service, Including US State News* Retrieved from https://ezproxy.canberra.edu.au/login?url=https://www.proquest.com/wire-feeds/international-patent-pfnature-co-ltd-주식회사-피에프네이처/docview/2914927403/se-2

The Grey Literature Report

https://www.greylit.org/

Search date: 21.05.2024

No	Search terms	Hits	After manual search
#1	wound cannabis	0	0
#2	wound marijuana	0	0
#3	wound cannabinoid	0	0
#4	skin cannabis	1	0
#5	skin marijuana	1	0
#6	skin cannabinoid	0	0

Boolean operators are not supported. Search is by keyword and terms are AND together.

UK national research register

https://discovery.nationalarchives.gov.uk/browse/r/r/C17376

Search date: 21.05.2024 (period: 2021-2024)

No	Search terms	Hits
#1	wound AND (cannabis OR hemp OR marijuana OR “phytocannabinoid*” OR “cannabinoid*” OR “cannabichromene*” OR “cannabicyclol*” OR “cannabidiol*” OR “cannabielsoin*” OR “cannabigerol*” OR “cannabinodiol*” OR “cannabinol*” OR “cannabitriol*” OR “tetrahydrocannabinol*”)	0
#2	skin AND (cannabis OR hemp OR marijuana OR “phytocannabinoid*” OR “cannabinoid*” OR “cannabichromene*” OR “cannabicyclol*” OR “cannabidiol*” OR “cannabielsoin*” OR “cannabigerol*” OR “cannabinodiol*” OR “cannabinol*” OR “cannabitriol*” OR “tetrahydrocannabinol*”)	0

World Health Organization Institutional Repository for Information Sharing

(WHO IRIS, https://apps.who.int/iris/)

Search date: 21.05.2024

No	Search terms	Date issues contains	Hits	After manual search
#1	wound AND cannabis	2021, 2022, 2023, 2024	0, 9, 6, 1	0
#2	wound AND marijuana		2, 3, 0, 0	0
#3	wound AND cannabinoid		1, 1, 3, 0	0
#4	skin AND cannabis		12, 8, 13, 3	0
#5	skin AND marijuana		7, 2, 2, 3	0

#6	skin AND cannabinoid		4, 2, 5, 2	0

(NLM) U.S. National Library of Medicine

https://www.nlm.nih.gov/

Search date: 21.05.2024

No	Search terms	Hits	After manual search
#1	wound AND (cannabis OR hemp OR marijuana OR phytocannabinoid* OR cannabinoid* OR cannabichromene* OR cannabicyclol* OR cannabidiol* OR cannabielsoin* OR cannabigerol* OR cannabinodiol* OR cannabinol* OR cannabitriol* OR tetrahydrocannabinol*)<br>filter: health information	11	0
#2	skin AND (cannabis OR hemp OR marijuana OR phytocannabinoid* OR cannabinoid* OR cannabichromene* OR cannabicyclol* OR cannabidiol* OR cannabielsoin* OR cannabigerol* OR cannabinodiol* OR cannabinol* OR cannabitriol* OR tetrahydrocannabinol*)<br>filter: health information	50	0

SciFINDER

Search date: 22.05.2024 (2021-2024)

No	Search terms	No of results
#1	antibacterial AND cannabinoid AND skin	15
#2	antibacterial AND phytocannabinoid AND skin	2
#3	cannabinoid AND wound	108
#4	phytocannabinoid AND wound	6

All downloaded in ris format and imported to endnote.

Table S3: Summary of *ex vivo* studies on wound healing potential

Table S3: Descriptive characteristics of *ex vivo* studies on antibacterial and wound healing potential of medicinal cannabis (n=13)

Study setting	Study design	Method/Assay	Experimental groups	Intervention	Outcome measure (s)	Treatment outcome (s)	Quality score
Blaskovich *et al* 2021[1]<br><br>Bench top	Experimental	*Ex vivo* porcine *Staphylococcus aureus* skin infection model (n=2-21)	Porcine tissue (from *Sus scrofa domesticus*) inoculated with methicillin resistant *S. aureus* (MRSA) ATCC 43300 (1×10^6 CFU/explant) and clinical isolates of *S. aureus*	12 cannabidiol (CBD)-containing topical formulations (containing CBD 5, 10, 15 or 20%)<br>**Comparator**<br>The respective vehicles, Untreated control	Log_{10}(CFU/explant) after 1h and 24 h of treatment	MRSA ATCC 43300 load was reduced significantly by formulations #3 (ointment containing 5% CBD) and #12 (gel containing 5% CBD) at 24 h ($p < 0.001$). Formulations #2 (liquid), #3 and #12 containing 20% CBD significantly reduced MRSA load with low and high levels of mupirocin resistance by 24 h.	**1**
del Rio *et al* 2016[2]<br><br>Bench top	Experimental	Wound healing by cell migration assay (n=3)	24 h old monolayer of normal human dermal fibroblasts (NHDFs) in a 96-well Essen ImageLock plate were scratched with a 96-pin Wound Maker and incubated with 10ng/mL of mitomycin C (to arrest cell proliferation). Imaged in every 60 min for 36 h.	VCE-004.8 (1, 5 or 10 μM) and TGFβ1 (wound healing inducer) or rhIL-4 (10ng/mL, wound healing inducer)<br>**Comparator**:<br>TGFβ1 alone,<br>Control	Relative Wound Density (%)	VCE-004.8 significantly inhibited wound healing induced by either TGFβ1 or rhIL-4	**1**
del Rio *et al* 2018[3]<br><br>Bench top	Experimental	Cell migration assay (n = 5)	Monolayer of NHDFs in 96-well Essen ImageLock plates were scratched with a 96-pin Wound Maker. Cell proliferation was inhibited by replacing culture medium with DMEM containing 1% antibiotics and mitomycin C (10 ng/mL). Imaged in every 3 h for 48 h.	VCE-004.3 (1, 5 or 10 μM) was added with TGFβ1 (10 ng/mL, wound healing inducer)<br>**Comparator**:<br>TGFβ1 alone,<br>Control	Percentage of wound confluence	Migration of fibroblasts induced by TGFβ1 was strongly inhibited by VCE-004.3 ($p<0.05$)	**1**

Table S3: Summary of *ex vivo* studies on wound healing potential

Moore 2019 (Maters thesis)[4]<br><br>Bench top	Experimental	Scratch wound healing assay	Linear scratch wound made in 24 h old monolayer of mouse NIH/3T3 fibroblasts (ATCC CRL-1658), treated and incubated for 24 h	44 µg/mL (50 µL/mL) Hemp oil in DMEM containing 0.1% glycerol and<br>1% spotted wintergreen (SWG) crude extract in hemp oil<br>**Comparator**<br>Vehicle (0.1% glycerol), 20% fetal bovine serum (positive control)	Percentage of scratch wound closure at 24 h	Hemp oil significantly increased cell migration (~40%) compared to vehicle (~20%).<br>20% FBS resulted ~60% cell migration.<br>Cell migration induced by 1% SWG crude extract in hemp oil as a carrier (~40%) was similar to that of hemp oil alone (~40%).	**1**
Sangiovanni *et al* 2019[5]<br><br>Bench top	Experimental	Quantitative polymerase chain reaction (PAHS-121Z Human Wound Healing PCR)	72 h old NHDFs (ATCC PCS-201-012™)	Cells were treated with TNFα (10 ng/mL) and ethanolic extract of *C. sativa* L. flowers (CSE) (25 µg/mL) or CBD (4 µM) for 6 h	Reduction of mRNA levels of 84 genes involved in wound healing in HDF cells	16 genes involved in wound healing were induced by the stimulus TNFα.<br>Transcription of all those genes upregulated by TNFα was counteracted by CSE.<br>CBD counteracted the effect of TNFα on 11 genes.	**1**
Wang et al 2016[6]<br><br>Bench top	Experimental	Scratch wound healing assay (n=5)	confluent monolayers of HaCaT cells were scratched, treated and incubated for 24 h	GP1a (40 nM to 25 µM), or AM630 (40 nM to 25 µM)<br>**Comparator**<br>Vehicle (0.1% DMSO)	Wound closure after 24 h	GP1a accelerated wound closure at 1 µM and 5 µM, and reduced wound closure at 25 µM compared to vehicle control.<br>AM630 inhibited wound closure at 5 µM and 25 µM compared to vehicle control.	**1**
Lephart 2023[7]<br><br>Bench top	Experimental	Quantitative polymerase chain reaction-messenger ribonucleic acid (qPCR-mRNA) analysis (n=4)	Epidermal full-thickness human skin cultures (EFT-400)	20 µL of either 0.3% CBD, 0.5% equol, or 0.3% CBD+0.5% equol for 24 h<br>**Comparator**<br>20 µL of transcutol (vehicle) for 24 h	Modulation of wound healing biomarkers	CBD significantly stimulated 4 out of the 12 wound healing biomarkers compared to vehicle control (FAF2, FETUB, KUTKG, LEP).<br>3 out of the 12 biomarkers were significantly modulated by equol over vehicle control and with 2 genes (KITLG showed stimulation and PJP1 displayed inhibition). These were significantly greater compared to CBD group.<br>11 out of the 12 biomarkers showed significantly greater modulation by CBD with Equol compared to the CBD or Equol only, suggesting improved wound healing, antioxidant effects and cell/tissue survival.	**1**

Table S3: Summary of *ex vivo* studies on wound healing potential

Monou et al 2022[8]<br><br>Bench top	Experimental	Scratch wound healing assay (n=3)	Confluent monolayers of HaCaT cells were scratched (two straight perpendicular scratches), treated and incubated for 12 h	CBD and cannabigerol (CBG) nanoparticles (0.1, 1, and 5 mg/mL)<br>**Comparator**<br>Serum-free medium (control)	Relative wound closure at 6 and 12 h	Relative wound area was reduced at 6 h for all the concentrations except for 5mg/mL CBD.<br>Relative wound area was increased at 12 h for all the concentrations of both drugs.<br>Except for 0.1 mg/mL CBG, all the other drugs increased the relative wound area at 12h more than initial wound area.	**1**
Kongkadee et al 2022[9]	Experimental	Scratch wound healing assay (n=3)	Confluent monolayers of human gingival fibroblast (HGF-1) cells were scratched and rinsed with drugs for 3 mins. Rinsing was repeated 3 times at 5–6 h intervals for 48 h.	1.5 mL of the hemp extract (5 µg/mL) or CBD (0.5 µg/mL) in serum-free media<br>**Comparator**<br>0.9% (w/v) NaCl (positive control), non-treated control	Percentage wound closure at 0, 12, 24, 36, 48 h after wounding	Significant wound closure by CBD at 24, 36 and 48h compared to non-treated control ($p<0.05$).<br>Significant wound closure by hemp extract at 24, and 36h compared to non-treated control ($p<0.05$).	**1**
Gerasymchuk et al 2022[10]	Experimental	Scratch wound healing assay (n=6)	**Experiment A:** Confluent monolayers of normal human neonatal foreskin fibroblasts [CCD-1064Sk (ATCC® CRL-2076™)] were scratched and treated	2µM delta-9-tetrahydrocannabinol (THC), 2µM CBD<br>**Comparator**<br>ISCOVE's Modified Dulbecco's Medium (IMDM) cell culture medium (untreated), DMSO (vehicle)	Percentage of unhealed wound observed at 1, 24, 48, and 72 h after wounding.	Significantly smaller percentage of unhealed wounds by THC compared to the vehicle after 24 h ($p < 0.05$) and after 48 h ($p < 0.001$).<br>Complete healing by THC in 72h.<br>Significantly smaller percentage of unhealed wounds by CBD compared to the vehicle by 48 h ($p < 0.01$).<br>No significant differences were observed at 72 h between any groups.	**1**
Gerasymchuk et al 2022[10]	Experimental	Scratch wound healing assay (n=6)	**Experiment B:** As above with stress-induced premature senescence (SIPS) fibroblasts. SIPS fibroblasts were prepared by incubating fibroblasts for 1 h with 25 µM of hydrogen peroxide.	Same as above	Same as above	Wound size was increased in hydrogen peroxide treated fibroblasts after 24.<br>THC ($p<0.05$) and CBD ($p<0.01$) significantly decreased the wound size in 24 h compared to vehicle.<br>THC and CBD significantly decreased the wound size in 72 h compared to vehicle ($p<0.001$).<br>Wounds induced in THC-SIPS fibroblasts were completely healed by 72h.	**1**

Table S3: Summary of *ex vivo* studies on wound healing potential

Gerasymchuk et al 2022[10]	Experimental	Scratch wound healing assay (n=6)	**Experiment C**: Confluent monolayers of normal human adult skin fibroblasts [CCD-1135Sk (ATCC® CRL-2691™)] were scratched and treated	2μM THC, 2μM CBD **Comparator** metformin (500 μM), triacetylresveratrol (10 μM), rapamycin (5 μM), or a mixed treatment of all nutrient signaling receptors (NSRs) and phytocannabinoids	Same as above	THC improved wound healing compared to the vehicle at 48h ($p < 0.05$). CBD did not heal the wound better than the vehicle. Mixed group healed the wound compared to vehicle at 72 h ($p < 0.001$).	
Montreekachon et al 2023[11]	Experimental	Scratch wound healing assay (n=3)	Confluent monolayers of human gingival fibroblasts (HGFs) were scratched and treated	0.3, 1, or 3 μM of CBD **Comparator** 1% (v/v) of methanol, DMEM with 10% (v/v) of fetal bovine serum (FBS) (positive control)	Wound closure (percentage of cell migration) 12 h interval for 72 h	Higher wound closure percentage by 1 μM CBD compared to other two doses of CBD and 1% (v/v) of methanol at all the time points. Percentage of cell migration by 1 μM CBD was significantly higher than methanol at 48, 60 and 72h. 10% (v/v) of FBS in DMEM closed the wound at 36 h.	**1**
Rouabhia et al 2023[12]	Experimental	Scratch wound healing assay (n=4)	**Experiment A**: Confluent monolayers of nasal epithelial cells were treated with drugs for 6 h, washed and scratched.	Cannabis smoke condensate (CSC) at various concentrations (0, 1, 5, 10, and 20 %) **Comparator** Fresh medium (negative control)	Distance separating two wound edges at 8, and 24 h	Negative control and 1% CSC closed the wounds completely at 24h. Significant amount of uncovered gap was observed between the wound edges in 8 and 24 h by the other CSC extracts (5, 10 and 20%) compared to control and 1% CSC ($p<0.001$). The uncovered distance increased with increasing CSC concentration at both time points.	**1**
Rouabhia et al 2023[12]	Experimental	Scratch wound healing assay (n=4)	**Experiment B**: Confluent monolayers of nasal epithelial cells were scratched and treated	Same as above	Same as above	Complete wound closure was only observed by the negative control at 24h. compared to control and 1% CSC, other CSC extracts (5, 10 and 20%) had significant amount of uncovered gap between the wound edges in 8 and 24 h ($p<0.001$). Wound closure at both time points by all the extracts were less with 24 h exposure compared to 6 h exposure.	**1**

Table S3: Summary of *ex vivo* studies on wound healing potential

Klinsang et al 2023[13]	Experimental	Scratch wound healing assay (n=not given)	Confluent monolayers of normal human dermal fibroblasts (NHDFs) were scratched and treated	A complex of cannabidiol and 2-hydroxypropyl-β-cyclodextrin (CBD/HP-β-CD) was incorporated into a fibroin-based film to prepare a film dressing. It was sterilized, cut into 1 × 1 cm^2 pieces, immersed in 1 mL of serum/antibiotic-free Dulbecco's Modified Eagle Medium (DMEM) and incubated at 37 °C for 24 h. The supernatants were filtered through a 0.22 μm membrane. 1 mL of the film-free supernatant was used as the treatment. **Comparator** serum/antibiotic-free medium (control)	The gap between wound edges were observed every 12 h for a total duration of 36h.	Gap was completely healed by film-free supernatant by 36h. Gap was not closed in control group at 36h.	**2**

Risk of bias of *ex vivo* studies were assessed by the Toxicological data Reliability Assessment Tool (ToxRTool)[14].

Table S4: Summary of excluded pre-clinical studies (Descriptive characteristics)

Table S4: Descriptive characteristics of excluded laboratory and field *in vivo* studies reporting wound healing

Study setting	Study design	Method/Assay	Intervention	Outcome measure (s)	Treatment outcome (s)	Quality score
Boehm et al 2021[15]<br><br>*In vivo*, field	Case study	A 30-year-old, intact female Indian rhinoceros (*Rhinocerus unicornis*) with ongoing erosive, ulcerative, non-pruritic and non-seasonal skin lesions for 4-years (8 lesions). Lesions were infected with *Staphylococcus dysgalactiae*, *Escherichia coli*, *Stenotrophomonas maltophilia*, *Corynebacterium spp*.	Dermoscent® PYOspot® spot-on (contains ajowan and neem extracts combined with oils of tamanu and hemp) twice weekly, one ampulla per lesional skin site.<br>Dermoscent® ATOP 7® spray (contains lavandin, manuka essential oils and N-acetylcysteine) twice daily,<br>Dermoscent® BIO BALM (contained cajuputoil and vegetable oil from soybean) twice daily.<br>All were directly applied on the lesional skin for 4 months.	Wound closure, Remission of infection	Skin lesions were considerably improved with reduced erythema and crusting on day 21.<br>No clinical signs of infection and minor growth of *Streptococcus dysgalactiae* on culture by week 3.<br>Complete clinical remission at 4 months without scarring.<br>No recurrence for the 2 years of follow-up.	**3**
Ghacham et al 2023[16]<br><br>*In vivo*, laboratory-reared	Experimental	Skin wound in mouse.<br>**Experimental group**<br>Linear incisions (~1.2cm) were made on the dorsal skin of 12–15 weeks old (30-33g) adult male mice. Drugs were applied daily on the wound for 9 days. (n=10 per group)	Essential oils from dried inflorescence of *Cannabis Sativa* L. (CSEO)<br><br>**Comparator**:<br>Control non-injured group, incision treated with povidone-iodine group and incision untreated group.	Percentage wound contraction	CSEO accelerated wound contraction compared to the untreated group and povidone-iodine on day 3, 6 and 9 ($p \leq 0.05$).	**3**

Table S4: Summary of excluded pre-clinical studies (Descriptive characteristics)

Christy et al 2024[17]<br><br>*In vivo*, laboratory-reared	Experimental	Deep partial-thickness burn wounds in pigs<br><br>**Experimental group**<br>Sixteen 25 cm^2 square, deep partial-thickness burn wounds per pig were made on dorsum and flanks of four Yorkshire Hybrid pigs (two male and two female, pathogen-free 50 and 70 kg). Wounds of each animal were randomly allocated to topical treatment (n=4).	Noneuphoric Phytocannabinoid Elixir 14 oil [NEPE14, a proprietary product. Contains a complex mixture of phytocannabinoids, including CBD, CBD acid, tetrahydrocannabinol acid, and essential fatty acids, (< 0.3%) of D9-tetrahydrocannabinol]. 7.5 mL of NEPE14 was applied on wound to make 3mm thick layer on day 0, 4 and 7. Wounds were covered with strips of Tegaderm (3M).<br>**Comparator**:<br>Vehicle control, Silverlon (standard of care control), no treatment (gauze only). Wounds were covered with strips of Tegaderm (3M).<br>All the pigs were covered with customized surgical swine jacket.	Clinically relevant bacterial infection (10^6 colony forming units) in wounds, percentage re-epithelialization on day 14	Percentage re-epithelialization on day 14 varied among treatments: NEPE14 (49.6±26.8%), vehicle control (28.8±21.7%), Silverlon (26.6±15.8%), and gauze (31.5±26.0%).<br>NEPE14 accelerated the re-epithelialization (not statistically significant).<br>On day 4, only gauze-treated burns showed signs of infection (25%).<br>On day 7, NEPE14 (50%), vehicle control (58%), Silverlon (67%), and gauze (33%) treated groups showed signs of infection.<br>On day 14, NEPE14 (42%), vehicle control (42%), Silverlon (42%), and gauze (33%) treated groups showed signs of infection.	**3**
da Silva et al 2024[18]<br><br>*In vivo*, field	Case study	A 2-year-old female mixed-breed dog (25.5kg) with discoid lupus erythematosus (DLE) presented with body condition score of 5 (on a scale of 1 to 9), epidermal scaling, depigmentation, and formation of crust in the nasal bridge area and in the nostrils. The standard care including corticosteroid was failed.	Oral full-spectrum CBD-rich oil (50mg/mL) and THC-dominant oil (40mg/mL) tailored depending on response for one year.	Lesion stabilisation	Nasal planum lesion size was reduced within few weeks and stabilized in week 6, with no further improvement or deterioration.	**3**

The experimental low-quality studies were not extracted.

Table S5: Summary of excluded pre-clinical studies (Formulation details)

Table S5: Formulation details of medicinal cannabis based products used in *in vivo* studies reported in table S4

Reference	Formulation and/or route of administration	Content
Boehm et al 2021[15]	Spot-on, Topical	Dermoscent® PYOspot® spot-on (contains ajowan and neem extracts combined with oils of tamanu and hemp)<br>Dose: one ampulla per lesional skin site.
Ghacham et al 2023[16]	Topical oil	Essential oils from dried inflorescence of *Cannabis Sativa* L. (CSEO)
Christy et al 2024[17]	Topical	Noneuphoric Phytocannabinoid Elixir 14 oil [NEPE14, a proprietary product. Contains a complex mixture of phytocannabinoids, including CBD, CBD acid, tetrahydrocannabinol acid, and essential fatty acids, (< 0.3%) of D9-tetrahydrocannabinol].<br>Dose: 7.5 mL was applied on wound to make 3mm thick layer
da Silva et al 2024[18]	Oral oil	Full-spectrum CBD-rich oil (50mg/mL) and THC-dominant oil (40mg/mL)<br>Dose: Tailored depending on the needs

Table S6: Summary of excluded clinical studies (Descriptive characteristics)

Table S6: Descriptive characteristics of excluded human studies

Study setting and location	Study design	Study population	Wound aetiology/ classification	Intervention descriptions		Outcome measures	Follow up	Study outcomes	Quality score
				Test	Comparator				
Maida 2017[19] Canada	Prospective case report	Malignant wound (n=1)<br>Age = 44 years<br>Gender = male	Malignant wound on cheek	Vaporized MC (THC 7.25% and CBD 8.21%) through a certified Volcano vaporizer unit.<br>Dose: 0.5-1.0 g of dried cannabis per day, vaporized every 2-4 hours and 15 minutes before daily wound dressing change.<br>Treatment was changed to topical MC in nongenetically modified organic sunflower oil (THC 5.24% and CBD 8.02%) after 4 months.<br>Dose: 1-2 mL of oil applied to wound both externally and intrabuccal, and any residual oil was swished throughout the oral cavity and swallowed, 4 times a day.	Condition before the treatment	Wound size,<br>Reduction in pain,<br>Reduction in analgesic usage	5 months	**Vaporized MC**<br>Discontinue and reduce the doses of analgesics<br>Pain reduction<br>Experienced less trismus and nausea, along with improved appetite, sleep, and effect<br>No negative effects from MC<br>Resumed career responsibilities again<br>Increase in wound size from 8.8 cm^2 to 44.2 cm^2 in 4 months<br><br>**Topical MC**<br>Wound size was decreased by 5% in 4 weeks<br>No adverse effects<br><br>Patient was globally deteriorated, and daily opioid use was doubled	**7**
Maida 2020[20] Canada	Prospective case report	Patient with sickle cell disease (n=1 patient, 3 wounds)<br>Age = 44 years<br>Gender = female	Chronic recurrent ulcers for 12 years	Daily topical application of VS-21 to the wound beds and VS-22 to a 4–6 cm radial cuff of peri-wound integument<br>Both formulations contain CBD (2.5 mg/mL), THC (<1 mg/mL), delta-9 tetrahydrocannabinolic acid (2.65 mg/mL), quercetin (31.25 mg/mL), disomin (25.31 mg/mL), hersperidin (2.5 mg/mL), and BCP (101.79 mg/mL) in different bases<br>Tissues were then covered with one layer each of Jelonet® and Mesorb®, followed by spiral bandaging	Condition before the treatment	Healing time,<br>Wound size	5 months	Complete wound healing was achieved<br>Average healing time 43.3 days<br>Deterioration of the wounds were observed if the treatment was ceased when wounds were healed 97%	**7**
Maida et al 2020[21] Canada	Prospective case report	patient with uremic calciphylaxis (n=1 patient, 2 wounds)<br>Age = 74 years<br>Gender = female	Chronic wounds more than 12 months	Formulations VS-12 and VS-14 were applied to the wound beds and 4 – 6 cm radial cuff of peri-wound areas respectively daily. Then the wounds were bandaged.	Condition before the treatment	Wound size	21 days	5-9% wound size reduction<br>59-78% increase in granulation tissue<br>No side effects	**7**

Table S7: Summary of excluded clinical studies (Formulation details)

Table S7: Formulation details of medicinal cannabis based products in human studies reported in table S6

Reference	Formulation and route of administration	Content
Maida 2017[19]	Vapor, inhalation, topical	ARGYLE™ (containing THC 7.25% þ CBD 8.21%) from TWEED, Inc. delivered through a certified Volcano™ vaporizer unit<br>Topical medicinal cannabis in nongenetically modified organic sunflower oil (THC 5.24% and CBD 8.02%)
Maida 2020[20]	Topical formulations	Formulation VS-21 (base: 1:1 v/v hyaluronic acid and *Aloe vera* gel) applied to the wound beds<br>Formulation VS-22 (liposomal base) were applied to 4 - 6 cm radial cuff of peri-wound areas<br>Both formulations contain CBD (2.5 mg/mL), THC (<1 mg/mL), delta-9 tetrahydrocannabinolic acid (2.65 mg/mL), quercetin (31.25 mg/mL), disomin (25.31 mg/mL), hersperidin (2.5 mg/mL), and BCP (101.79 mg/mL)
Maida et al 2020[21]	Topical formulations	Same as above

Supplementary material S2: Details of Risk of bias assessment tools

In vivo and ex vivo studies were evaluated for risk of bias (RoB) using the Toxicological data Reliability assessment Tool (ToxRTool)[14]. The criteria for assessing RoB and assigning a reliability category used by the ToxRTool were identification of test substance, characterization of test organism, description of study design, documentation of study results and plausibility of study design and results[14]. The user can deviate from automatic categorization by providing reasons[14]. The criteria ranged from 1-4; 1 being the reliable without restrictions, 2 is reliable with restrictions, 3 is not reliable and 4 is not assignable.

All the in vivo studies included in this review were reliable without restrictions. However, purity of the test substance was not reported by three studies[1,22,23], source of the test substance was not reported by one study[22], age/body weight of the test organism was not reported by one study[1], complete study results for all the end point measurements was not reported by two studies[23,24].

Joanna Briggs Institute's (JBI) critical appraisal tools were used to assess the methodological quality in human studies. Risk of bias associated with case reports (which reports on 4 patients or less individually as single cases[25]) and case series (report the grouped data of 5 patients or more[25]) were assessed by critical appraisal checklist for case reports[26] and critical appraisal checklist for case series[27] respectively. Randomised controlled trials were assessed by the JBI critical appraisal tool for assessment of risk of bias for randomized controlled trials[28].

Criteria in assessing RoB used by JBI critical appraisal checklist for case reports were on reporting of patient's demographic characteristics, history, current and post-intervention clinical condition, results of diagnostic tests and/or assessments, interventions, adverse and/or unanticipated events and takeaway lessons[26]. Criteria in assessing RoB used by JBI critical appraisal checklist for case series were on reporting of inclusion of participants (criterian, and consecutive, complete inclusion), identification and measurement of the condition, demographic details, clinical details, and outcomes[27]. Criteria in assessing RoB used by JBI critical appraisal tool for the assessment of risk of bias for randomized controlled trials were on bias related to selection and allocation; administration of intervention/exposure; assessment, detection and measurement of the outcome; and participant retention[28]. It also includes statistical conclusion validity[28].

Study quality scores varied from 5-7 on the JBI critical appraisal tools for case studies and case series (8 being the highest quality). Clear descriptions were not found on patient demographic characteristics in 3 studies[29-31], and on patient history in 3 studies[30-32]. Details on treatment procedure was unclear in one study[29], adverse effects were unclear in one study[31]. With respect to the case series, it was unclear whether it has consecutive inclusion of patients[33], and statistical analysis[33]. It had not reported complete inclusion of participants, and clear reporting of the presenting site(s)/clinic(s) demographic infornation[33].

Study quality scores of the included randomised controlled trial[34] was 10. The highest score in the respective tool for randomised controlled trials[28] is 13.

Supplementary material S3: Details of rejected articles

Following are the details of rejected articles which were rejected at the full-text screening.

Ten (10) studies[35-44] were excluded since they do not contain adequate data to be included in this systematic review. Four (4) studies[45-48] were excluded because they were duplicates (1 article was a commentary on an already included study[45], one was a media statement[46], and two were a thesis and their respective journal article has been included in the full-text review[47,48]).

Sixteen (16) studies[49-64] were excluded since they were patents. Five (5) study[65-69] was excluded because of wrong indication. Thirteen (13) articles[70-82] were excluded due to they did not look for wound healing or antibacterial properties of *Cannabis sativa* extracts or cannabinoids in in vivo or in humans (wrong outcomes). Eleven (11) studies[83-93] were excluded since they were wrong study designs. Eleven (11) articles[2-5,7-13] were excluded because they were ex vivo studies on integumentary wound healing. Four (4) articles[15-18] with poor quality (scored 3 in ToxRTool) were excluded.

Table S8: Outcome measures of the selected studies

Table S8: Outcomes measures of the selected studies

Reference	Outcome	Description as per the article	Time points	Funding source
**ex vivo studies**				
del Rio et al 2016	Wound healing by cell migration assay: Relative Wound Density (%)	NDA	Hourly up to 36 h	Government
del Rio et al 2018	Cell migration assay: Percentage of wound confluence (closure)	NDA	Every 3h up to 48h	Government, European Commission
Sangiovanni et al 2019	Quantitative polymerase chain reaction: Reduction of mRNA levels of 84 genes involved in wound healing	NDA	For 6h	Government, Industry
Moore 2019	Scratch wound healing assay: Percentage of scratch wound closure	NDA<br>Calculated relative to the vehicle control	0h and 24h	NG
Blaskovich et al 2021	ex vivo porcine skin *S. aureus* infection model: Reduction in bacterial load	Log_{10}(CFU/explant) after 1h and 24 h of treatment<br>CFU is defined as the number of colony forming units remaining on 5 mm biopsy explants inoculated with 2 ± 0.5 µL of ~5 × 10^8 CFU/mL MRSA ATCC 43300 and incubated at 37 °C in 6-well plates containing a 0.4 µm trans-well insert	1h and 24h	Government, Industry, Competing interests revealed
Lephart 2023	Quantitative polymerase chain reaction-messenger ribonucleic acid (qPCR-mRNA) analysis: Modulation of wound healing biomarkers	Gene expression inhibition was detected by significant lower copy numbers and gene expression stimulation was detected by significant higher copy numbers compared to vehicle control numbers for each biomarker.	24h	University
Monou et al 2022	Scratch wound healing assay: Relative wound closure	Percentage of wound closure compared to initial wound size	6 and 12 h	None
Kongkadee et al 2022	Scratch wound healing assay: Percentage wound closure	Percentage of wound closure compared to initial wound size	0, 12, 24, 36, 48 h	Government, project
Gerasymchuk et al 2022	Scratch wound healing assay: Percentage of unhealed wound	Percentage by which the original scratch width has decreased for each time point	1 h, 24 h, 48 h, and 72 h	Government, project
Montreekachon et al 2023	Scratch wound healing assay: percentage of cell migration	Percentage area of the wound closed compared to initial wound area	12 h interval for 72 h	Government, University
Rouabhia et al 2023	Scratch wound healing assay: Distance separating two wound edges	Distance separating the two cell monolayer edges	8 and 24 h	Foundation
Klinsang et al 2023	Scratch wound healing assay: Closure of gap between wound edges	NDA	every 12 h for 36 h	Government, University

Table S8: Outcome measures of the selected studies

**in vivo studies**				
Mehrabani et al 2016	Third degree burns in mice: Wound area	Wound area was measured (in mm^2) by tracing the wound boundaries using image J software (http://rsbweb.nih.gov/ij) in every 3 days	Measured on day 0, and every 3 days until complete wound healing	University
	Third degree burns in mice: Rate of wound healing	Determined by measuring the size of the lesions in every 3 days. The wound area on day 1 was considered as 100% and the wound areas on subsequent days were compared with the initial wound area	Every 3 days until complete wound healing	
	Third degree burns in mice: Epithelialization time	Epithelialization time was monitored until the scars that covered the burn lesion surface fell off	Until complete wound healing	
	Third degree burns in mice: Percentage of wound contraction	Wound contraction (%) = (wound area of day 0 - wound area of day x) / wound area of day 0 × 100 Where x = 0, 4, 7, 10, 14, 18, or 21	Measured on day 0, and every 3 days until complete wound healing	
Wang et al 2016	Percentage wound size	The original wound sizes (measured by tracing the dermal border of the wound).	at 0.5, 1, 3, 5, 7, 10, 13, 17 and 21 days	Government, Projects
	Wound re-epithelialization percentage.	NDA	Same as above	
Klein et al 2018	Oral wound healing in rats: Wound area	The largest (D) and smallest (d) diameters of the wound were measured. The area of the ulcer was calculated using the following formula: $A = \pi \times D/2 \times d/2$	Day 0, 3 and 7	Government, University
McIver et al 2020	Second intension wound healing in the equine model: Wound area	Advancing edge of epithelium on each side of the granulation bed was measured	Day 1, 7, 14, 21, 28, 35, 42 after wound creation	Industry
	Second intension wound healing in the equine model: Overall time to complete healing	Wounds were considered completely healed when the granulation tissue was no longer visible	Day 1 and day at which wound was completely healed	
	Second intension wound healing in the equine model: Overall rate of wound healing (cm^2/day)	Rate = wound area on day 1/total days to complete healing	Day 1 and day at which wound was completely healed	
Blaskovich et al 2021	Bioluminescent in vivo mouse skin infection model: Reduction in *S. aureus* load at 48 h compared to vehicle	CFU's per mice	At 48 h	Government, Industry, Competing interests revealed

Table S8: Outcome measures of the selected studies

Zhao et al 2021	Full thickness skin wound in mouse: epithelial sheet length	Epithelial sheet length = the distance between the wound bed to the leading edge of the epidermis along the epidermal-dermal border.	epithelial sheet length: 4-6 days	Government, Project, University Competing interests revealed
	Full thickness skin wound in mouse: Time to complete wound healing	NDA	Followed up to 20 days	
Zheng et al 2022	Full thickness skin wound in rats: Percentage of remaining wound area	NDA	3, 7, 10, and 14 days	Government, Project
Zhong et al 2022	in vivo mouse skin infection model for acute and chronic wounds: Relative wound size	NDA	Day 1, 3, 5, 7 and 9	Government, Project
	in vivo mouse skin infection model for acute and chronic wounds: CFU count in the wound on day 2 of treatment.	NDA	Day 2	
McCormick et al 2023	Murine model of cutaneous lupus erythematosus: lesion score	scored weekly for developing lesions and severity of existing lesions, using a skin plaque scoring protocol	20 weeks	Industry
Cham et al 2024	In vivo mice dermal infection model: number of CFU in a dissected 1 cm^2 skin patch	Number of colony forming units in a dissected 1 cm^2 skin patch of the infected wound from the mouse	Daily for 5 days	Government, Project
**Human**				
Maida and Corban 2017	Reduction in average daily pain score	11-point numeric rating scale (0 to 10) was used Percentage in average daily pain score after the initiation of cannabis treatment comparative to pre-treatment	9-33 weeks (depends on the patient)	NG
	Average daily morphine sulfate equivalent (MSE) usage	Assessed before and after the treatment	9-33 weeks (depends on the patient)	
Chelliah et al 2018	Healing time	NDA	Not reported	NG
	Reduction in blistering	NDA	Not reported	
	Reduction in pain	NDA	Not reported	
Schrader et al 2019	Pain reduction	Pain on the visual analogue scale	8 months – 2 years	NG
	Reduction in pruritus	Reduction in the frequency and intensity of the pruritus	8 months – 2 years	
	Reduction in the overall intake of analgesic medications	Before and after medicinal cannabis initiation and at different time points after initiation of medicinal cannabis therapy	8 months – 2 years	
Maida et al 2020	Wound healing/full closure	Complete reepithelization of the wound	Up to 2.6 months	NG,

Table S8: Outcome measures of the selected studies

	Time for complete wound closure	Time elapsed from treatment initiation to complete wound closure	Up to 2.6 months	Conflict of interest revealed
	Reduction in pain	With patient's level of distress, ambulation, and reduction in analgesic usage	Up to 2.6 months	
	Reduction in analgesic opioid usage	Percentage reduction of analgesic requirement compared to pre-treatment analgesic need	Up to 2.6 months	
Maida et al 2021	Complete closure of the wound	Defined as being fully epithelialized	Up to 150 days	NG, Conflict of interest revealed
	Healing time	Time elapsed from treatment initiation to complete wound closure	Up to 150 days	
Diaz et al 2021	Complete closure of the wound	NDA	2 months	NG
	Healing time	NDA	2 months	
Umpreecha et al 2023	Ulcer size	Calculated using the formulas for the surface area of a circle or ellipse using the diameters.	0, 2, 5, and 7 days	University
	Daily pain ratings	visual analog scale (VAS) consisting of a 100-mm horizontal line between the edges marked "no pain" and "unbearable pain" were marked daily by the participants	For 7 days	

NDA: not defined in the article; NG: not given

Supplementary material S4: Amendments to PROSPERO registration from the initial submission

Named contact was changed from Dhakshila Niyangoda to Jackson Thomas. Email salutation was changed from Dhakshila to Jackson. Named contact email was changed from dhakshila.niyangoda@canberra.edu.au to jackson.thomas@canberra.edu.au. Named contact phone number was changed from +94763494638 to +61423127345.

Review team members order was changed from Dhakshila Niyangoda, Jackson Thomas, Wubshet Tesfaye to Dhakshila Niyangoda, Wubshet Tesfaye, Jackson Thomas.

Dr Wubshet Tesfaye's affiliation was changed from Health Research Institute, University of Canberra to Pharmacy School, University of Sydney, Sydney, New South Wales.

Dr Wubshet Tesfaye's email address was changed from wubshet.tesfaye@canberra.edu.au to wubshet.tesfaye@sydney.edu.au.

Review question was changed from "Do cannabis, cannabis derived extracts or secondary metabolites of cannabis demonstrate antibacterial and wound healing effects *in ex vivo, in vivo and* human studies?" to "Do cannabis, cannabis derived extracts or secondary metabolites of cannabis demonstrate antibacterial and wound healing effects in *in vivo* and human studies?" (The term "ex vivo" was removed from the review question.)

Searches was edited by adding SciFINDER.

Participants/population was edited by replacing "Any patient group with diagnosis of wound irrespective of sociodemographic background will be included. Depending on the search results studies conducted with any animal with induced wound and/or existing wound and/or animal or human cells/tissue may be included" with "Any patient group with diagnosis of wound or infection irrespective of sociodemographic background will be included. Studies conducted with any animal with induced or existing wound and/or infection will be included." (Ex vivo studies were excluded from the targeted population.)

Intervention(s), exposure(s) was edited by replacing the sentence "These strategies will include ex vivo, in vivo, and clinical treatments." with "These strategies will *include* in vivo, and clinical treatments." (Ex vivo studies were excluded.)

Types of study to be included was edited by replacing "Studies that investigated the effectiveness of cannabis, cannabis derived extracts and secondary metabolites of cannabis in wound healing and as antibacterials in ex vivo, in animals or in humans will be included. The study designs to be targeted include ex vivo studies, pre-clinical animal studies, randomised controlled trials (RCTs), non-randomized controlled studies, clinical trials, or case series containing more than 5 patients." with "Studies that investigated the effectiveness of cannabis, cannabis derived extracts and secondary metabolites of cannabis in wound healing and as antibacterials in animals or in humans will be included.

The study designs to be targeted include pre-clinical animal studies, randomised controlled trials (RCTs), non-randomized controlled studies, clinical trials, or case series of more than 5 patients." (*Ex vivo* studies were excluded.)

Context was edited by removing "Ex vivo studies might employ any cell and/or tissue of animal or human origin."

Main outcome(s) was edited by removing "For cells and tissues, values defined with respect to the conducted assay (eg: number of colonies,) will be considered.". "Bacterial load: difference from initial load after specified period of time" was added to the Measures of effect.

Data extraction (selection and coding) was edited by removing "For laboratory studies (ex vivo studies) information that will be extracted include study design, assay, study treatment, outcomes, and cytotoxicity/adverse events."

Risk of bias (quality) assessment was edited by replacing "Joanna Briggs Institute's (JBI) critical appraisal tools will be used to assess the methodological quality." with "Joanna Briggs Institute's (JBI) critical appraisal tools will be used to assess the methodological quality of human studies. ToxRTool (Toxicological data reliability assessment) tool will be used to assess the methodological quality of animal studies."

Supplementary material S4: Amendments to PROSPERO registration from the initial submission

Analysis of subgroups or subsets was edited by removing the phrase "*ex vivo*,"
Type and method of review was edited by ticking narrative synthesis.
Keywords was edited by removing "cannabis" and adding "medicinal cannabis", and "antimicrobial resistance".
Current review status was changed from ongoing to completed but not published.

References

The supplementary materials were prepared as per the PRISMA 2020 guidelines which needs to include the studies that appear meeting the inclusion criteria but rejected in the full-text review. They were cited here.

1. Blaskovich, M.A.T.; Kavanagh, A.M.; Elliott, A.G.; Zhang, B.; Ramu, S.; Amado, M.; Lowe, G.J.; Hinton, A.O.; Do Minh Thu, P.; Zuegg, J.; et al. The antimicrobial potential of cannabidiol. *Communications Biology* **2021**, *4*, 7, doi:10.1038/s42003-020-01530-y.
2. del Rio, C.; Navarrete, C.; Collado, J.A.; Bellido, M.L.; Gómez-Cañas, M.; Pazos, M.R.; Fernández-Ruiz, J.; Pollastro, F.; Appendino, G.; Calzado, M.A.; et al. The cannabinoid quinol VCE-004.8 alleviates bleomycin-induced scleroderma and exerts potent antifibrotic effects through peroxisome proliferator-activated receptor-γ and CB2 pathways. *Sci Rep* **2016**, *6*, 21703, doi:10.1038/srep21703.
3. del Rio, C.; Cantarero, I.; Palomares, B.; Gomez-Canas, M.; Fernandez-Ruiz, J.; Pavicic, C.; Garcia-Martin, A.; Luz Bellido, M.; Ortega-Castro, R.; Perez-Sanchez, C.; et al. VCE-004.3, a cannabidiol aminoquinone derivative, prevents bleomycin-induced skin fibrosis and inflammation through PPARγ- and CB2 receptor-dependent pathways. *British Journal of Pharmacology* **2018**, *175*, 3813-3831.
4. Moore, E.M. Combined Therapeutic Potential of Spotted Wintergreen and Botanical Oils in Dermatology. M.Sc. Thesis, North Carolina State University, Raleigh, North Carolina, US, 2019.
5. Sangiovanni, E.; Fumagalli, M.; Pacchetti, B.; Piazza, S.; Magnavacca, A.; Khalilpour, S.; Melzi, G.; Martinelli, G.; Dell'Agli, M. Cannabis sativa L. extract and cannabidiol inhibit in vitro mediators of skin inflammation and wound injury. *Phytotherapy Research* **2019**, *33*, 2083-2093, doi:10.1002/ptr.6400.
6. Wang, L.L.; Zhao, R.; Li, J.Y.; Li, S.S.; Liu, M.; Wang, M.; Zhang, M.Z.; Dong, W.W.; Jiang, S.K.; Zhang, M.; et al. Pharmacological activation of cannabinoid 2 receptor attenuates inflammation, fibrogenesis, and promotes re-epithelialization during skin wound healing. *European Journal of Pharmacology* **2016**, *786*, 128-136, doi:10.1016/j.ejphar.2016.06.006.
7. Lephart, E.D. Cannabidiol (CBD) with 4′,7-Isoflavandiol (Equol) Efficacy is Greater than CBD or Equol Treatment Alone via Human Skin Gene Expression Analysis. *Front. Biosci. Landmark* **2023**, *28*, 154, doi:10.31083/j.fbl2807154.
8. Monou, P.K.; Mamaligka, A.M.; Tzimtzimis, E.K.; Tzetzis, D.; Vergkizi-Nikolakaki, S.; Vizirianakis, I.S.; Andriotis, E.G.; Eleftheriadis, G.K.; Fatouros, D.G. Fabrication and Preliminary In Vitro Evaluation of 3D-Printed Alginate Films with Cannabidiol (CBD) and Cannabigerol (CBG) Nanoparticles for Potential Wound-Healing Applications. *Pharmaceutics* **2022**, *14*, 1637, doi:10.3390/pharmaceutics14081637.
9. Kongkadee, K.; Wisuitiprot, W.; Ingkaninan, K.; Waranuch, N. Anti-inflammation and gingival wound healing activities of Cannabis sativa L. subsp. sativa (hemp) extract and cannabidiol: An in vitro study. *Archives of oral biology* **2022**, *140*, 105464, doi:10.1016/j.archoralbio.2022.105464.
10. Gerasymchuk, M.; Robinson, G.I.; Groves, A.; Haselhorst, L.; Nandakumar, S.; Stahl, C.; Kovalchuk, O.; Kovalchuk, I. Phytocannabinoids Stimulate Rejuvenation and Prevent Cellular Senescence in Human Dermal Fibroblasts. *Cells* **2022**, *11*, 3939, doi:10.3390/cells11233939.
11. Montreekachon, P.; Chaichana, N.; Makeudom, A.; Kerdvongbundit, V.; Krisanaprakornkit, W.; Krisanaprakornkit, S. Proliferative effect of cannabidiol in human gingival fibroblasts via the mitogen-activated extracellular signal-regulated kinase (MEK) 1/2. *Journal of Periodontal Research* **2023**, *58*, 1223-1234, doi:10.1111/jre.13178.
12. Rouabhia, M.; Piché, M.; Hazzi, C.; Corriveau, M.N.; Chakir, J. Effect of cannabis smoke condensate on human nasal epithelial cell adhesion, growth, and migration. *American Journal of Otolaryngology - Head and Neck Medicine and Surgery* **2023**, *44*, 103890, doi:10.1016/j.amjoto.2023.103890.

13. Klinsang, T.; Charoensit, P.; Phimnuan, P.; Luangpraditkun, K.; Ross, G.M.; Viennet, C.; Ross, S.; Viyoch, J. In Vitro Wound Healing Potential of a Fibroin Film Incorporating a Cannabidiol/2-Hydroxypropyl-β-cyclodextrin Complex. *Pharmaceutics* **2023**, *15*, 2682, doi:10.3390/pharmaceutics15122682.
14. Schneider, K.; Schwarz, M.; Burkholder, I.; Kopp-Schneider, A.; Edler, L.; Kinsner-Ovaskainen, A.; Hartung, T.; Hoffmann, S. "ToxRTool", a new tool to assess the reliability of toxicological data. *Toxicology Letters* **2009**, *189*, 138-144, doi:https://doi.org/10.1016/j.toxlet.2009.05.013.
15. Boehm, T.M.S.A.; Klinger, C.J.; Gohl, C.; Lücht, M.; Baumann, K.N.; Mueller, R.S. Topical treatment of multiple erosive, ulcerative skin lesions in an Indian rhinoceros (Rhinoceros unicornis). *Tierarztl. Prax. Ausg. G. Grosstiere Nutztiere* **2021**, *49*, 210-214, doi:10.1055/a-1475-3459.
16. Ghacham, S.E.; Bakali, I.E.; Zarouki, M.A.; Ali, Y.A.E.H.; Ismaili, R.; Ayadi, A.E.; Souhail, B.; Tamegart, L.; Azzouz, A. Wound healing efficacy of Cannabis sativa L. essential oil in a mouse incisional wound model: A possible link with stress and anxiety. *South African Journal of Botany* **2023**, *163*, 488-496, doi:10.1016/j.sajb.2023.11.005.
17. Christy, S.; Carlsson, A.H.; Larson, D.; Davenport, G.J.; Glenn, J.F.; Brumfield, R.; Avina, G.; Jockheck-Clark, A.; Christy, R.J.; Nuutila, K. Topical Noneuphoric Phytocannabinoid Elixir 14 Reduces Inflammation and Mitigates Burn Progression. *Journal of Surgical Research* **2024**, *296*, 447-455, doi:10.1016/j.jss.2024.01.014.
18. da Silva, M.E.S.; Christianetti, B.; Amazonas, E.; Pereira, M.L. Case report: Cannabinoid therapy for discoid lupus erythematosus in a dog. *Front Vet Sci* **2024**, *11*, 1309167, doi:10.3389/fvets.2024.1309167.
19. Maida, V. Medical cannabis in the palliation of malignant wounds—A Case Report. *Journal of Pain and Symptom Management* **2017**, *53*, e4-e6, doi:https://doi.org/10.1016/j.jpainsymman.2016.09.003.
20. Maida, V.; Shi, R.B.; Fazzari, F.G.T.; Zomparelli, L.M. A new treatment paradigm for sickle cell disease leg ulcers: Topical cannabis-based medicines. *Experimental Dermatology* **2021**, *30*, 291-293, doi:10.1111/exd.14256.
21. Maida, V.; Shi, R.B.; Fazzari, F.G.T.; Zomparelli, L. Promoting wound healing of uremic calciphylaxis leg ulcers using topical cannabis-based medicines. *Dermatologic therapy* **2020**, *33*, e14419-n/a, doi:10.1111/dth.14419.
22. McIver, V.C.; Tsang, A.S.; Symonds, N.E.; Perkins, N.R.; Uquillas, E.; Dart, C.M.; Jeffcott, L.B.; Dart, A.J. Effects of topical treatment of cannabidiol extract in a unique manuka factor 5 manuka honey carrier on second intention wound healing on equine distal limb wounds: a preliminary study. *Australian Veterinary Journal* **2020**, *98*, 250-255, doi:10.1111/avj.12932.
23. Koyama, S.; Purk, A.; Kaur, M.; Soini, H.A.; Novotny, M.V.; Davis, K.; Cheng Kao, C.; Matsunami, H.; Mescher, A. Beta-caryophyllene enhances wound healing through multiple routes. *PLoS One* **2019**, *14*, e0216104, doi:10.1371/journal.pone.0216104.
24. Klein, M.; de Quadros De Bortolli, J.; Guimaraes, F.S.; Salum, F.G.; Cherubini, K.; de Figueiredo, M.A.Z. Effects of cannabidiol, a Cannabis sativa constituent, on oral wound healing process in rats: Clinical and histological evaluation. *Phytother. Res.* **2018**, *32*, 2275-2281, doi:10.1002/ptr.6165.
25. Abu-Zidan, F.M.; Abbas, A.K.; Hefny, A.F. Clinical "case series": A concept analysis. *African Health Sciences* **2012**, *12*, 557-562, doi:10.4314/ahs.v12i4.25.
26. Moola, S.; Munn, Z.; Tufanaru, C.; Aromataris, E.; Sears, K.; Sfetcu, R.; Currie, M.; Lisy, K.; Qureshi, R.; Mattis, P.; et al. Chapter 7: Systematic reviews of etiology and risk In *JBI Manual for Evidence Synthesis*, Aromataris, E., Munn, Z., Eds.; JBI: 2020 doi:10.46658/JBIMES-20-08 (Accessed on 28 January 2022).
27. Munn, Z.; Barker, T.H.; Moola, S.; Tufanaru, C.; Stern, C.; McArthur, A.; Stephenson, M.; Aromataris, E. Methodological quality of case series studies: an introduction to the JBI critical appraisal tool. *JBI Evidence Synthesis* **2020**, *18*, 2127-2133, doi:10.11124/JBISRIR-D-19-00099.

28. Barker, T.H.; Stone, J.C.; Sears, K.; Klugar, M.; Tufanaru, C.; Leonardi-Bee, J.; Aromataris, E.; Munn, Z. The revised JBI critical appraisal tool for the assessment of risk of bias for randomized controlled trials. *JBI Evidence Synthesis* **2023**, *21*, 494-506.
29. Chelliah, M.P.; Zinn, Z.; Khuu, P.; Teng, J.M.C. Self-initiated use of topical cannabidiol oil for epidermolysis bullosa. *Pediatric Dermatology* **2018**, *35*, E224-E227, doi:10.1111/pde.13545.
30. Schräder, N.H.B.; Duipmans, J.C.; Molenbuur, B.; Wolff, A.P.; Jonkman, M.F. Combined tetrahydrocannabinol and cannabidiol to treat pain in epidermolysis bullosa: a report of three cases. *British Journal of Dermatology* **2019**, *180*, 922-924, doi:10.1111/bjd.17341.
31. Maida, V.; Corban, J. Topical medical cannabis: A new treatment for wound pain-three cases of pyoderma gangrenosum. *Journal of Pain & Symptom Management* **2017**, *54*, 732-736, doi:10.1016/j.jpainsymman.2017.06.005.
32. Maida, V.; Shi, R.B.; Fazzari, F.G.T.; Zomparelli, L. Topical cannabis-based medicines – A novel paradigm and treatment for non-uremic calciphylaxis leg ulcers: An open label trial. *International Wound Journal* **2020**, *17*, 1508-1516, doi:10.1111/iwj.13484.
33. Maida, V.; Shi, R.B.; Fazzari, F.G.T.; Zomparelli, L. Topical cannabis-based medicines - A novel adjuvant treatment for venous leg ulcers: An open-label trial. *Experimental Dermatology* **2021**, *30*, 1258–1267, doi:10.1111/exd.14395.
34. Umpreecha, C.; Bhalang, K.; Charnvanich, D.; Luckanagul, J. Efficacy and safety of topical 0.1% cannabidiol for managing recurrent aphthous ulcers: a randomized controlled trial. *BMC Complement Med Ther* **2023**, *23*, 57, doi:10.1186/s12906-023-03886-0.
35. Ciaglia, E.; Lamberti, A.; Cuomo, G.; Faggiana, G.; Abate, M.; Ranieri, R.; Lembo, S.; Balato, A.; Monfrecola, G.; Bifulco, M. Modulation of inflammatory milieu in skin diseases: Novel multi-actions of the non-psychotropic plant-derived cannabinoid, Cannabidiol. *Journal of Investigative Dermatology* **2016**, *136*, S204.
36. Cocchiara, E.; Spinella, A.; Magnani, L.; Lumetti, F.; Palermo, A.; Baiocchi, G.; Salvarani, C.; Giuggioli, D. Cannabinoids in the treatment of pain related to systemic sclerosis skin ulcers: Our experience. *Annals of the Rheumatic Diseases* **2019**, *78*, 1784-1784, doi:10.1136/annrheumdis-2019-eular.6004.
37. Klein, M.; De Quadros De Bortolli, J.; Borghetti, R.L.; Jacoby, L.S.; Salum, F.G.; Cherubini, K.; De Figueiredo, M.A.Z. Effects of cannabidiol, a cannabis sativa constituent, on oral wound healing. *Oral Surgery, Oral Medicine, Oral Pathology & Oral Radiology* **2020**, *129*, e166-e167, doi:10.1016/j.oooo.2019.06.711.
38. Schuetz, M.; Savile, C.; Webb, C.; Rouzard, K.; Fernandez, J.R.; Perez, E. Cannabigerol: The mother of cannabinoids demonstrates a broad spectrum of anti-inflammatory and anti-microbial properties important for skin. *Journal of Investigative Dermatology* **2021**, *141*, S83-S83.
39. Tomida, I.; Azuara–Blanco, A.; Pu, J.; Zhao, M.; Pertwee, R.; Forrester, J.V. Effects of the synthetic cannabinoid WIN 55,212–2 on corneal epithelial wound healing in vitro. *Investigative Ophthalmology & Visual Science* **2004**, *45*, 4873-4873.
40. Narla, S.; Price, K.N.; Sachdeva, M.; Shah, M.; Shi, V.; Hamzavi, I.; Alavi, A.; Lowes, M.A. Proceeding report of the Fourth Symposium on Hidradenitis Suppurativa Advances 2019. *Journal of the American Academy of Dermatology* **2021**, *84*, 120-129, doi:10.1016/j.jaad.2020.05.114.
41. Larson, D.A.; Carlsson, A.H.; Nuutila, K.; Christy, S.E.; Jockheck-Clark, A.R.; Christy, R.J. 613 Evaluation of Non-Euphoric Phytocannabinoid Elixir 14 (NEPE-14) Application in Deep Partial-Thickness Burn Wounds. In Proceedings of the American Burn Association 54th Annual Meeting, 2022; p. S146.
42. Cristina, A.; Laura, Z.; Oliviero, M.; Conf. Hemp extract safety for dermatological application. 2021.
43. Yılmaz, E.; Usta, M.; Conf. Fabrication of hemp seed oil-doped gelatin/sodium alginate nanofibers using the Box-Behnken experimental design. In Proceedings of the 5th International Eurasian Conference on Biological and Chemical Sciences (EurasianBioChem 2022), Ankara, Turkey 2022; p. 344.

44. Jimenez-Rodriguez, C.; Santana-del-Pino, A.; Jimenez-Diaz, J.F.; Hernandez-Martinez, F.; Rodriguez-de-Vera, B.C. Cutaneous ulceration scattering induced topically by cannabidiol oil in the laboratory animal. *Eur. J. Public Health* **2019**, *29*, 6.
45. Kiefer, D. Topical cannabis for wound pain: A case series. *Integrative Medicine Alert* **2017**, *20*, 140-141.
46. ISRCTN16488940. Can a cannabis-based medicine applied to a non-healing wound improve healing and reduce pain? **2020**. doi:10.1186/isrctn16488940 (Accessed on 19 May 2023)
47. McIver, V.C. Studies on the effect of various topical agents on second intention wound healing of the equine distal limb. Masters of Veterinary Clinical Studies Thesis, University of Sydney, 2020.
48. Mamaligka, A.M. 3D-Printed Alginate Films With CBD And CBG Nanoparticles For Potential Wound-Healing Applications (Ανάπτυξη τρισδιάστατα εκτυπωμένων υμενίων από αλγινικό νάτριο με νανοσωματίδια cbd και cbg και αξιολόγηση της συνεισφοράς τους στην επούλωση πληγών). Masters Thesis, Aristotle University of Thessaloniki, Thessaloniki, 2022.
49. Callahan, M.; Thurn, M. Antibacterial treatment using cannabinoid combinations for treatment of bacterial infection. WO/2020/000024, 2020. Available online: https://patentscope.wipo.int/search/en/detail.jsf?docId=WO2020000024 (accessed on 14 August 2021)
50. Callahan, M.; Thurn, M. Antibacterial dosage regime using cannabinoids. AU2020297660A1, 2020. Available online: https://patents.google.com/patent/AU2020297660A1/en (accessed on 14 December 2021)
51. Christensen, J. Compositions of fulvic acid and cannabinoid and uses thereof. 20220184026. 2022. Available online: https://patents.justia.com/patent/20220184026 (accessed on 17 June 2022)
52. Fabries, L. Dermo-cosmetic composition for pets. US9333186B2, 2012. Available online: https://patents.google.com/patent/US9333186B2/en (accessed on 14 August 2021)
53. Hartenbach, J. Cannabinoid and CBD liposome formulations and uses thereof. US20210030678A1. Available online: https://patents.google.com/patent/US20210030678A1/en (accessed on 18 August 2022)
54. Kennedy, J.P. Compositions for treating dermatological diseases. WO2021003488, 2021. Available online: https://patentscope.wipo.int/search/en/detail.jsf?docId=WO2021003488 (accessed on 14 August 2021)
55. Popp, K.F.; Stiefel, B.D. Topical anti-acne composition. US6433024B1, 2021. Available online: https://patents.google.com/patent/US6433024B1/en (accessed on 18 August 2022)
56. Maida, V. Topical formulations and instillates, kits, and methods for treating integumentary wounds, and uses thereof. CA3028706A1, 2021. Available online: https://patents.google.com/patent/CA3028706A1/en (accessed on 18 August 2022)
57. Maida, V. Topical cannabinoid formulations and instillates, kits, and methods for treating integumentary wounds, and uses thereof. WO2019191830, 2019. Available online: https://patentscope.wipo.int/search/en/detail.jsf?docId=WO2019191830 (accessed on 16 August 2021)
58. Murphy, B.; El Sohly, M.; Gul, W.; Jacob, M. Cannabinoids for the treatment of Gram-positive infections including antibiotic-resistant bacterial strains. WO2020051284, 2020. Available online: Cannabinoids for the treatment of Gram-positive infections including antibiotic-resistant bacterial strains (accessed on 16 August 2021)
59. Palaio, P. Methods of accelerating wound healing using cannabinoid compositions. 20200376156A1, 2020. Available online: https://www.freepatentsonline.com/y2020/0376156.html (accessed on 15 August 2021)
60. Postrel, R. Process and Method to Accelerate Cellular Regeneration, Healing and Wound Management. 20190060220, 2019. Available online: https://patents.justia.com/patent/20190060220 (accessed on 19 August 2022)
61. Raz, N.; Eyal, A.M. Terpene-enriched cannabinoid compositions and uses thereof in the treatment of infectious conditions. EP3793542A2, 2019. Available online: https://patents.google.com/patent/EP3793542A2/en (accessed on 19 August 2021)

62. Raz, N.; Eyal, A.M. Terpene-enriched cannabinoid compositions and uses thereof in the treatment of infectious conditions. 20210059978, 2021. Available online: https://patents.justia.com/inventor/noa-raz?page=2 (accessed on 20 August 2021)
63. Zimmer, A.; Karsak, M.; Werner, S. Methods for identifying modulators of CB1 and CB2 cannabinoid receptors and their use in wound healing. WO2006111424A1, 2006. Available online: https://patents.google.com/patent/WO2006111424A1/en?q=(cannabinoid)&inventor=andreas+zimmer (accessed on 20 August 2021)
64. Greenspan, M.H.; Norval, C.P. Topical cannabinoid compositions, delivery systems, and uses for pain relief. US20210015740, 2021. Available online: https://patents.google.com/patent/US20210015740A1/en (accessed on 20 August 2021)
65. Simpson, A.C.; Bradley, C.W.; Schissler, J.R. Probable cutaneous adverse drug reaction due to a cannabidiol-containing hemp oil product in a dog. *Veterinary Dermatology* **2020**, *31*, 404-e108, doi:10.1111/vde.12876.
66. Thathapudi, N.C.; Groleau, M.; Degué, D.S.; Aghajanzadeh Kiyaseh, M.; Kujawa, P.; Soulhi, F.; Akla, N.; Griffith, M.; Robert, M.-C. Novel micellar CB2 receptor agonist with anti-inflammatory action for treating corneal alkali burns in a mouse model. *Frontiers in pharmacology* **2023**, *14*, 1270699, doi:10.3389/fphar.2023.1270699.
67. Fu, R.Z. Cannabidiol and Ascorbic Acid Promotes Epithelial Cell Migration in an In Vitro Model of Wound Healing. M.Sc. Thesis, Adelphi University, New York, US, 2022.
68. Ferrini, F.; Donati Zeppa, S.; Fraternale, D.; Carrabs, V.; Annibalini, G.; Verardo, G.; Gorassini, A.; Albertini, M.C.; Ismail, T.; Fimognari, C.; et al. Characterization of the Biological Activity of the Ethanolic Extract from the Roots of Cannabis sativa L. Grown in Aeroponics. *Antioxidants (Basel)* **2022**, *11*, 860, doi:10.3390/antiox11050860.
69. Yu, L.; Zeng, L.; Zhang, Z.; Zhu, G.; Xu, Z.; Xia, J.; Weng, J.; Li, J.; Pathak, J.L. Cannabidiol Rescues TNF-α-Inhibited Proliferation, Migration, and Osteogenic/Odontogenic Differentiation of Dental Pulp Stem Cells. *Biomolecules* **2023**, *13*, 118, doi:10.3390/biom13010118.
70. Atif, A.; Naveed, A. The safety and efficacy of 3% Cannabis seeds extract cream for reduction of human cheek skin sebum and erythema content. *Pak J Pharm Sci* **2015**, *28*, 1389-1395.
71. Casares, L.; García, V.; Garrido-Rodríguez, M.; Millán, E.; Collado, J.A.; García-Martín, A.; Peñarando, J.; Calzado, M.A.; de la Vega, L.; Muñoz, E. Cannabidiol induces antioxidant pathways in keratinocytes by targeting BACH1. *Redox Biology* **2020**, *28*, 101321, doi:10.1016/j.redox.2019.101321.
72. Dahham, S.S.; Tabana, Y.M.; Ahamed, M.B.K.; Majid, A.M.S.A. In vivo anti-inflammatory activity of β-caryophyllene, evaluated by molecular imaging. *Molecules & Medicinal Chemistry* **2015**, *1*, e1001, doi:10.14800/mmc.1001.
73. Jin, S.; Lee, M.-Y. The ameliorative effect of hemp seed hexane extracts on the Propionibacterium acnes-induced inflammation and lipogenesis in sebocytes. *PLoS One* **2018**, *13*, e0202933, doi:10.1371/journal.pone.0202933.
74. Oláh, A.; Tóth, B.I.; Borbíró, I.; Sugawara, K.; Szöllõsi, A.G.; Czifra, G.; Pál, B.; Ambrus, L.; Kloepper, J.; Camera, E.; et al. Cannabidiol exerts sebostatic and antiinflammatory effects on human sebocytes. *J Clin Invest* **2014**, *124*, 3713–3724.
75. Palmieri, B.; Laurino, C.; Vadalà, M. A therapeutic effect of cbd-enriched ointment in inflammatory skin diseases and cutaneous scars. *La Clinica terapeutica* **2019**, *170*, e93-e99, doi:10.7417/CT.2019.2116.
76. Sangiovanni, E.; Fumagalli, M.; Pacchetti, B.; Piazza, S.; Magnavacca, A.; Khalilpour, S.; Melzi, G.; Martinelli, G.; Dell'Agli, M. Cannabis sativa L. extract reduces inflammatory markers in human fibroblasts and keratinocytes. *PLANTA MEDICA* **2019**, *85*, 1407.
77. Zagórska-Dziok, M.; Bujak, T.; Ziemlewska, A.; Nizioł-Łukaszewska, Z. Positive Effect of Cannabis sativa L. Herb Extracts on Skin Cells and Assessment of Cannabinoid-Based Hydrogels Properties. *Molecules* **2021**, *26*, 802.

78. Perez, E.; Fernandez, J.R.; Fitzgerald, C.; Rouzard, K.; Tamura, M.; Savile, C. In Vitro and Clinical Evaluation of Cannabigerol (CBG) Produced via Yeast Biosynthesis: A Cannabinoid with a Broad Range of Anti-Inflammatory and Skin Health-Boosting Properties. *Molecules* **2022**, *27*, 491.
79. Cohen, G.; Jakus, J.; Baroud, S.; Gvirtz, R.; Rozenblat, S. Development of an Effective Acne Treatment Based on CBD and Herbal Extracts: Preliminary in Vitro, Ex Vivo, and Clinical Evaluation. *Evidence-based Complementary and Alternative Medicine* **2023**, *2023*, 4474255, doi:10.1155/2023/4474255.
80. Purida, J. The efficacy and safety of topical hemp seed extract in treatment of acne vulgaris: a split-face, double-blinded, randomized, controlled trial. Masters Thesis, Thammasat University, Bangkok, Thailand, 2021.
81. Aryannejad, A.; Eslami, F.; Shayan, M.; Noroozi, N.; Hedayatyanfard, K.; Tavangar, S.M.; Jafari, R.M.; Jour. Cannabidiol Improves Random-Pattern Skin Flap Survival in Rats: Involvement of Cannabinoid Type-2 Receptors. *Journal of Reconstructive Microsurgery* **2023**, *39*, 48-58.
82. Aryannejad, A.; Noroozi, N.; Tavangar, S.M.; Ramezani, S.; Rashidian, A.; Laripour, R.; Yousefi Zoshk, M.; Dehpour, A.R.; Chamanara, M. Involvement of Cannabinoid Type 2 Receptors in the Favorable Effects of Sumatriptan on the Random-Pattern Skin Flap Survival in Rats: A Novel Potential Target. *European Surgical Research* **2022**, *63*, 203-210, doi:10.1159/000521581.
83. Menjivar, J.; Bendaoud, M. Anti-Biofilm Properties of Flax, Chia, and Hemp Seed Oil Extracts. *Faseb Journal* **2020**, *34*, 1, doi:10.1096/fasebj.2020.34.s1.05146.
84. Posoldová, K. Příprava kosmetických výrobků proti lupům s využitím technického konopí ; Preparation of anti-dandruff cosmetic products using technical hemp. Vysoké učení technické v Brně. Fakulta chemická, 2020.
85. Žáčková, K. Využití technického konopí do kosmetiky proti akné; Use of technical hemp in acne preparations. Master Thesis, Brno University of Technology, Brno, Czechia, 2018.
86. Antezana, P.E.; Municoy, S.; Orive, G.; Desimone, M.F. Design of a New 3D Gelatin—Alginate Scaffold Loaded with Cannabis sativa Oil. *Polymers* **2022**, *14*, 4506, doi:10.3390/polym14214506.
87. Atalay, S.; Gęgotek, A.; Domingues, P.; Skrzydlewska, E. Protective effects of cannabidiol on the membrane proteins of skin keratinocytes exposed to hydrogen peroxide via participation in the proteostasis network. *Redox biology* **2021**, *46*, 102074, doi:10.1016/j.redox.2021.102074.
88. Zitek, T.; Bjelic, D.; Kotnik, P.; Golle, A.; Jurgec, S.; Potocnik, U.; Knez, Z.; Finsgar, M.; Krajnc, I.; Krajnc, I.; et al. Natural Hemp-Ginger Extract and Its Biological and Therapeutic Efficacy. *Molecules* **2022**, *27*, 7694, doi:10.3390/molecules27227694.
89. Ahmad, F.; Mushtaq, B.; Ahmad, S.; Rasheed, A.; Nawab, Y. A Novel Composite of Hemp Fiber and Alginate Hydrogel for Wound Dressings. *Journal of Polymers and the Environment* **2023**, *31*, 2294-2305, doi:10.1007/s10924-023-02756-7.
90. Antezana, P.E.; Municoy, S.; Pérez, C.J.; Desimone, M.F. Collagen Hydrogels Loaded with Silver Nanoparticles and Cannabis Sativa Oil. *Antibiotics (Basel, Switzerland)* **2021**, *10*, 1420, doi:10.3390/antibiotics10111420.
91. Chelminiak-Dudkiewicz, D.; Smolarkiewicz-Wyczachowski, A.; Mylkie, K.; Wujak, M.; Mlynarczyk, D.T.; Nowak, P.; Bocian, S.; Goslinski, T.; Ziegler-Borowska, M. Chitosan-based films with cannabis oil as a base material for wound dressing application. *Scientific reports* **2022**, *12*, 18658, doi:10.1038/s41598-022-23506-0.
92. Tiligada, Z. Study of newer active ingredients (cannabinoids and prebiotics/probiotics) and their incorporation into skin care products. Master Thesis, University of West Attica, Athens, Greece, 2023.
93. Chelminiak-Dudkiewicz, D.; Machacek, M.; Dlugaszewska, J.; Wujak, M.; Smolarkiewicz-Wyczachowski, A.; Bocian, S.; Mylkie, K.; Goslinski, T.; Marszall, M.P.; Ziegler-Borowska, M. Fabrication and characterization of new levan@CBD biocomposite sponges as potential materials in natural, non-toxic wound dressing applications. *International Journal of Biological Macromolecules* **2023**, *253*, 126933, doi:10.1016/j.ijbiomac.2023.126933.
